# EEG biomarkers of microstructural damage in normal-appearing white matter among patients with neuromyelitis optica spectrum disorder: A DTI-EEG combined study

**DOI:** 10.3389/fimmu.2026.1676066

**Published:** 2026-02-25

**Authors:** Lili Yang, Hui Qiu, Yun Qin, Zijun Li, Congyu Xu, Yan Xie, Kai Chen, Shuai Ma, Lingling Dong, Dezhong Yao, Rui Huang, Song Tan

**Affiliations:** 1 Department of Neurology, Sichuan Provincial People’s Hospital, University of Electronic Science and Technology of China, Chengdu, China; 2 Department of Neurology, Tongjiang County People’s Hospital, Bazhong, China; 3 Department of Radiology, Sichuan Provincial People’s Hospital, University of Electronic Science and Technology of China, Chengdu, China; 4 Key Laboratory for NeuroInformation of Ministry of Education, School of Life Science and Technology, University of Electronic Science and Technology of China, Chengdu, China; 5 Chengdu Techman Software Co., Ltd, Chengdu, China

**Keywords:** diffusion tensor imaging, functional connectivity, neuromyelitis optica, resting-state EEG, white matter microstructural damage

## Abstract

**Objective:**

This study aimed to explore the potential of EEG in monitoring the extent of white matter microstructural damage in patients with neuromyelitis optica spectrum disorder (pwNMOSD) for the first time.

**Methods:**

Thirty-two pwNMOSD and 20 healthy controls were recruited and all received DTI scan, while the pwNMOSD underwent also resting state EEG (rs-EEG). DTI indices were compared between two groups to identify impaired WM tracts in pwNMOSD. Correlations between the 240 rs-EEG indices (including spectral and functional connectivity indices in five frequency bands of six brain regions) and the fractional anisotropy (FA) of the impaired WM fiber tract were calculated to identify the rs-EEG biomarkers of WM microstructural damage. The relationships of the identified rs-EEG biomarkers with disease characteristics and cognitive function were further analyzed.

**Results:**

Seventeen of the 20 main WM tracts were found to have microstructural damage in pwNMOSD. The functional connectivity indices were significantly and positively correlated with the FA of WM tracts in pwNMOSD, especially the coherence and phase locking value strengths in the theta and gamma frequency bands (r=0.40-0.61). Different patterns between the rs-EEG indices in different frequency bands and the integrity of WM tracts were revealed. These identified rs-EEG indices were significantly related to patients’ disease characteristics (number of attacks (r=-0.44), expanded disability status scale score (r=-0.41--0.47), serum anti-glial fibrillary acidic protein level (r=-0.43)) and cognitive functions (symbol digit modality test score, r=0.46).

**Discussion:**

The functional connectivity of rs-EEG may serve as important biomarkersof WM microstructural damage in NMOSD.

## Background

Neuromyelitis optica spectrum disorder (NMOSD) is an inflammatory demyelinating central nervous system (CNS) disease (1, 2) with an estimated global pooled prevalence of 1.82/100000 people (3). In the Chinese population, the incidence has reached 3.31/100000 (4). The disorder is characterized by monophasic or recurrent inflammatory attacks of the optic nerve, spinal cord, brainstem, and cerebrum (1), causing vision loss, motor and sensory impairment, and other debilitating symptoms (1, 3, 5).

In addition to inflammatory outbreaks, increasing evidence suggests that patients with NMOSD (pwNMOSD) exhibit extensive white matter (WM) microstructural damage in the normal-appearing WM (NAWM) of the whole brain (6–9). This type of microstructural damage has also been shown to significantly affect the brain function of pwNMOSD, especially their cognitive function (8, 10, 11). Given the widespread presence of WM microstructural damage and its impact on brain function, long-term monitoring of the severity of WM microstructural damage in pwNMOSD is essential. Currently, the detection and assessment of WM microstructural damage in pwNMOSD largely depend on diffusion tensor imaging (DTI). However, DTI is time-consuming and has high requirements with respect to equipment and postprocessing technology. In China, only senior medical institutions could clinical perform DTI, while the detection of the integrity indices of WM tracts (e.g., fractional anisotropy (FA)) also requires additional postprocessing since the instrumental supporting visual processing procedures can only roughly observe the morphology of WM tracts, thereby limiting the routine evaluation of WM microstructural damage in clinical practice. Thus, it is essential to identify additional and convenient biomarkers of WM microstructural damage.

Resting-state electroencephalography (rs-EEG) recordings can be used to monitor spontaneous baseline neural activity, thus providing an overall reflection of functional impairments and adaptations in the brain (12–14). Previous studies combining DTI and rs-EEG have demonstrated that the rs-EEG index could reflect the integrity of WM through monitoring functional alternations in brain activity (15–19). Quantitative rs-EEG power spectrum analysis reflects the internal and inherent patterns of neural activity signals in the brain, whereas rs-EEG functional connectivity analysis reveals statistically significant synchronization between two or more electrode pairs, thereby reflecting the organization of the brain network (20). Research on mild traumatic brain injury (TBI) and stroke has revealed significant correlations between changes in the integrity indices of WM tracts (e.g., fractional anisotropy (FA)) and changes in the functional connectivity indices of rs-EEG (e.g., phase synchrony and coherence) (15–18). In addition to functional connectivity, studies in thalamic stroke patients and TBI patients reported correlations between slow rhythm power and the FA values of WM (19, 21). Our previous study revealed that pwNMOSD shows increased slow rhythm power in rs-EEG (22). Thus, since EEG has the advantages of convenience, limited time consumption, and cost effectiveness, it might be an appropriate technique for monitoring microstructural damage in the NAWM of pwNMOSD.

In the present study, we applied a multimodal framework that combines DTI and EEG to explore the functional alternation patterns of rs-EEG activity associated with WM microstructural damage in pwNMOSD and to identify rs-EEG biomarkers indicating its severity for the first time. This study deepens our understanding of the impact of WM microstructural damage on the brain activity of pwNMOSD and provide a more convenient and concise measure to monitor disease severity.

## Methods

### Participants

This study was approved by the ethics committee of Sichuan Provincial People’s Hospital, China, and written informed consent was obtained from each participant. The inclusion criteria for pwNMOSD were as follows: verified NMOSD diagnosis according to the 2015 International Panel for Neuromyelitis Optica Diagnosis criteria (1) and aged between 18 and 70 years. The exclusion criteria were as follows: 1) history of drug or alcohol abuse or other significant neurological diseases other than NMOSD; 2) receiving acute immunoregulatory treatment; 3) cerebral lesions; 4) contraindications for MRI scans or EEG recordings; and 5) taking any psychotropic drugs (such as sedative sleeping pills and antipsychotic drugs) in the past two weeks.

The demographic data and disease features of the patients were collected, including sex, age, education level, disease duration, number of attacks, presence of serum antibodies associated with CNS demyelinating disease (including autoantibodies to aquaporin 4 (AQP4-Ab), anti-myelin oligodendrocyte glycoprotein antibody (MOG-Ab), anti-glial fibrillary acidic protein antibody (GFAP-Ab)), degree of disability, clinical phenotype, and current preventive therapy. The degree of disability was evaluated by two neurologists according to the expanded disability status scale (EDSS) (23). Furthermore, all pwNMOSD received cognitive function evaluations, including the paced auditory serial addition test (PASAT) score (assessing auditory attention) and the symbol digit modality test (SDMT) score (assessing executive function).

The serum samples from pwNMOSD were centrifuged at 1,250 × g and stored at -80 °C. The serum level of GFAP was measured via ELISA (NBP3-11815, UNIV). The assays were performed according to the manufacturer’s protocols and by the same technique.

Demographically matched HCs were recruited from the population of individuals who attended the hospital for annual health check-ups, with no major clinical or psychiatric conditions and no contraindications for MRI scans. Their demographic data and cognitive functions (PASAT and SDMT scores) were also collected.

A total of 32 pwNMOSD and 20 HCs were enrolled in this study.

### Magnetic resonance image acquisition

All the participants underwent MRI scans, which were performed with a 3T MRI scanner (GE DISCOVERY MR750) via a 32-channel head coil. High-resolution 3-dimensional T1-weighted images, T2-weighted fluid-attenuated inversion recovery images, and diffusion-weighted images were acquired. The details of the sequences are included in the **Supplementary Material**.

### Image processing of DTI

Data processing was performed in MATLAB R2018b (MathWorks, Inc., USA). The Pipeline for Analyzing the Brain Diffusion Image (PANDA) (24) package was used for data preprocessing and for the evaluation of WM tract integrity.

We processed the images as follows: (1) converted the DTI from DICOM format to the 4D NIfTI format; (2) removed the nonbrain tissue; (3) corrected the image distortion caused by the eddy current and head movement; (4) coregistered the DTI data to the high-resolution 3D-T1 images; subsequently, the coregistered images were normalized to the Montreal Neurological Institute (MNI) 152 space; and (5) obtained the mean FA, mean diffusion (MD), axial diffusivity (AD), and radial diffusivity (RD) of each WM fiber tract based on the JHU white matter tractography atlas. The JHU white matter tractography atlas provides 20 important WM fiber tracts, including the bilateral anterior thalamic radiation (ATR), bilateral corticospinal tract (CST), bilateral cingulum cingulate gyrus (CGC), bilateral cingulum-hippocampus (CGH), forceps major (Fmaj), forceps minor (Fmin), bilateral inferior fronto-occipital fasciculus (IFO), bilateral inferior longitudinal fasciculus (ILF), bilateral superior longitudinal fasciculus (SLF), bilateral uncinate fasciculus (UNC), and bilateral superior longitudinal fasciculus-temporal part (SLFt) (6, 25).

### Resting-state EEG recording and data preprocessing

All pwNMOSD underwent five minutes of eyes-closed task-free rs-EEG recording, which was performed via a standardized protocol via the Biosemi EEG recording system. The rs-EEG data were acquired from 32 electrodes in accordance with the International 10–20 System. The participants were required to keep their eyes closed and remain stationary during the recording.

EEG data processing was performed in EEGLAB (26). The raw EEG data were visually inspected, and a 180-s window of high-quality data for each participant was selected. The EEG data were resampled to 512 Hz, high-pass filtered (1 Hz), and low-pass filtered (40 Hz). The data were rereferenced via the REST method (27). Independent component analysis was subsequently used to remove blinking and noise artifacts.

### EEG power spectrum analysis and functional connectivity analysis

The rs-EEG data were divided into several 5-s segments, and time–frequency analysis was performed on each segment via fast Fourier transform (FFT). The absolute power was obtained for each electrode in the different frequency bands. The frequency bands were divided into delta (1–4 Hz), theta (4–8 Hz), alpha (8–12 Hz), beta (12–30 Hz), and gamma (30–40 Hz) bands. Relative power was obtained by normalizing the power in each frequency band with the overall power in the range of 1–40 Hz within each channel. Moreover, several widely studied power ratio indices, including alpha/theta, alpha/delta, delta/theta, theta/beta, and alpha/beta, were also included in this study. One patient with poor-quality rs-EEG data was excluded. Ultimately, the subsequent analysis included the rs-EEG data from 31 pwNMOSD.

Functional connectivity assesses functional communication between brain areas by estimating the level of synchronization of the EEG signals. The freely available toolbox HERMES (28) was used to estimate functional connectivity in five frequency bands. Common indices of functional connectivity, that is, coherence (COH), imaginary coherence (iCOH), phase locking value (PLV), phase lag index (PLI), and weighted PLI (wPLI), were included. The functional connectivity per electrode was estimated by averaging the values for each possible electrode pair per electrode (for example, the value of electrode Fz is the average of the functional connectivity value between Fz and the other 31 electrodes).

To represent the EEG features of different brain regions, we extracted the abovementioned 40 EEG features (three power spectrum indices and five functional connectivity indices in five frequency bands) from 6 key electrode channels, i.e., Fz (frontal region), C3 (left brain), C4 (right brain), Cz (central region), Pz (parietal region), and Oz (occipital region). The channels were selected mainly on the basis of previous literature (16), in which the Oz channel was added to represent the occipital region. Finally, for each NMOSD patient, 240 rs-EEG indices were extracted for statistical analysis.

### Statistical analysis

Fisher’s exact test was first used to compare the sex ratios between the NMOSD and HC groups. The Shapiro–Wilk test was used to assess the normality of the quantitative data; all of the variables were normally distributed except the PASAT scores in the NMOSD group. Hence, independent-sample t tests were used to compare age, education, and SDMT scores between groups, and the Mann–Whitney U test was used to compare PASAT scores between groups. These statistical analyses were carried out using GraphPad Prism (version 8; San Diego, CA).

The DTI and EEG indices were analyzed using MATLAB R2019a. Specifically, the DTI indices of all 20 WM fiber tracts were compared between the NMOSD and HC groups via independent samples t tests if the data followed a normal distribution; otherwise, Wilcoxon rank-sum tests were used. Pearson’s correlation analysis (for normally distributed variables) or Spearman’s correlation analysis (for nonnormally distributed variables) was conducted to assess the relationships between the 240 EEG indices and the mean FA/MD/RD values of the WM fiber tracts with microstructural damage (with age as a covariate).

We further analyzed the relationships between the identified EEG biomarkers for WM microstructural damage and disease characteristics as well as cognitive functions in pwNMOSD. Two-sample t tests or Wilcoxon rank-sum tests were conducted to compare the recognized EEG indices between AQP4-Ab-positive and AQP4-Ab-negative patients. Analysis of variance (ANOVA) or the Kruskal–Wallis test was used to test the differences in recognized EEG indices among patients with different clinical phenotypes as well as among patients undergoing different preventive treatments. Pearson’s correlation analysis or Spearman’s correlation analysis was used to assess the correlations between the EEG indices and the number of clinical attacks, disease duration, EDSS score, and serum GFAP level (with age as a covariate), as well as the PASAT and SDMT scores (with age and education years as covariates), separately. False discovery rate (FDR) corrections (29) were applied to all the above statistical results. The statistical results were considered significant when P < 0.05 after false discovery rate (FDR) correction.

## Results

### Participant characteristics

**Table 1** summarizes the participant characteristics. All the participants were right-handed. There were no significant differences in age, sex, or education level between the NMOSD group and the HC group (P > 0.05). Compared with HCs, pwNMOSD presented significantly worse cognitive function based on the SDMT (P = 0.03) and PASAT scores (P < 0.001).

**Table 1:** Demographics, clinical characteristics, and cognitive function evaluation in patients with NMOSD and HCs.

Characteristics	NMOSD (n=31)	HC (n=20)	*P* value
Age (years), mean [SD] (range)	41.5 [15.4] (18–69)	45 [11.7] (22-65)	NS
Gender (F/M), N (%)	23 (74.2%)	17 (85%)	NS
Education (Year), mean [SD] (range)	9.8 [5.1] (0-16)	11.9 [3.9] (4-16)	NS
AQP4-Ab positive, N(%)	26 (83.9%)	–	
Serum GFAP values (pg/ml), mean [SD] (range)	111.2 [79.0] (12.9-283.8), n=25		
Number of attacks, mean [SD] (range)	4.1 [3.7] (1-13)	–	
Disease duration (year), mean [SD] (range)	4.5 [4.3] (0.5-20)	–	
EDSS, mean [SD] (range)	3.2 [2.2] (0-7.5)	–	
Clinical phenotype, N (%)			
ON	3 (9.6%)	–	
TM	11 (35.4%)	–	
ON+TM	17 (54.8%)	–	
Therapy, N(%)			
MMF/AZA	22 (70.9%)	–	
RTX	7 (22.5%)	–	
Other	2 (6.4%)		
Cognition tests			
PASAT, mean [SD] (range)	39.2 [8.4] (24-50), n=24	50.2 [6.4] (38-59), n=20	**<0.01**
SDMT, mean [SD] (range)	53.5 [15.2] (28-78), n=24	62.6 [12.2] (40-79), n=20	**0.03**

NMOSD, neuromyelitis optica spectrum disorder; HC, healthy control; F, female; M, male; AQP4-Ab, aquaporin-4-antibody; GFAP, glial fibrillary acidic protein; EDSS, expanded disability status scale; ON, optic neuritis; TM, transverse myelitis; MMF, mycophenolate mofetil; AZA, azathioprine; RTX, rituximab; PASAT, paced auditory serial addition test; SDMT, symbol digit modality test; NS, no statistical significance. The comparisons with statistical significance between the two groups are marked with bold *P* values.

The disease characteristics of the pwNMOSD are also displayed in **Table 1**. Additionally, in the five AQP4-Ab-negative patients, 2 were MOG-Ab positive, and 3 were negative for all three antibodies; in the two patients who used other therapies, one patient received a periodic subcutaneous injection of satralizumab, and the other received low-dose prednisolone.

### Comparisons of white matter tract integrity between pwNMOSD and HCs

**Figure 1:**
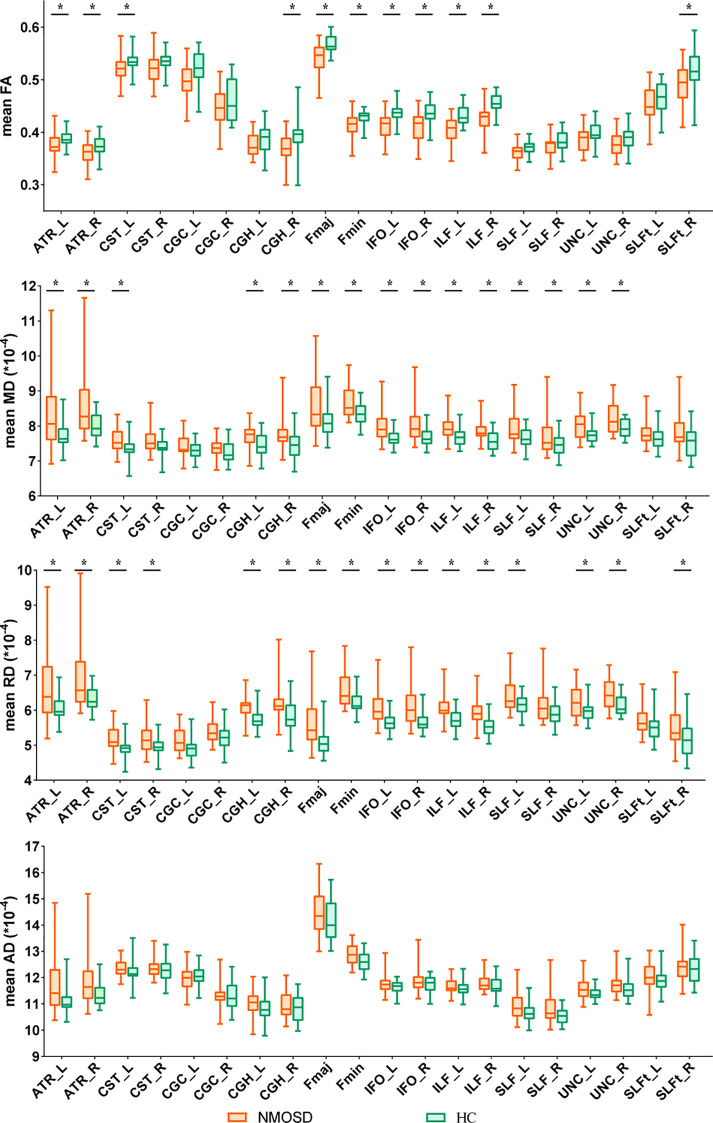
Group differences in entire white matter fiber tracts of diffusion tensor imaging (DTI) metrics, such as fractional anisotropy (FA), mean diffusion (MD), radial diffusivity (RD), and axial diffusivity (AD), were observed. Statistically significant differences are marked as * (P < 0.05 after false discovery rate correction, two-sample t tests or the Wilcoxon rank-sum test). ATR, anterior thalamic radiation; CST, corticospinal tract; CGC, cingulum (cingulate.gyrus); CGH, cingulum. (hippocampus); Fmaj, forceps major; Fmin, forceps minor; IFO, inferior fronto-occipital fasciculus; ILF, inferior longitudinal fasciculus; SLF, superior longitudinal fasciculus; UNC, uncinate fasciculus; SLFt, superior longitudinal fasciculus (temporal part); L, left; R, right; NMOSD, neuromyelitis optica spectrum disorder; HC, healthy control.

To identify damaged WM tracts in pwNMOSD, we investigated the differences in the DTI indices of WM tracts between pwNMOSD and HCs (**Figure 1**). In terms of the FA values of the 20 main WM fiber tracts, compared with HCs, pwNMOSD had significantly lower mean FA values in 11 WM tracts (t = -4.08~-2.35). With respect to the mean MD of the WM fiber tract, pwNMOSD presented significant increases in 15 WM tracts (t = 2.17-3.43). With respect to the mean RD of the WM fiber tract, pwNMOSD presented significant increases in 16 WM tracts (t = 2.38–3.96). However, after FDR correction, the differences in the mean AD values of the WM fiber tracts between the NMOSD group and HCs were no longer significant.

These results revealed widespread DTI index changes in 17 of the 20 main WM tracts among pwNMOSD (with decreased FA or increased MD/RD). Since none of the participants included in this study had any brain lesions, the abnormalities in the above white matter integrity indices in the pwNMOSD could reflect extensive WM microstructural damage to the NAWM.

**Figure 2:**
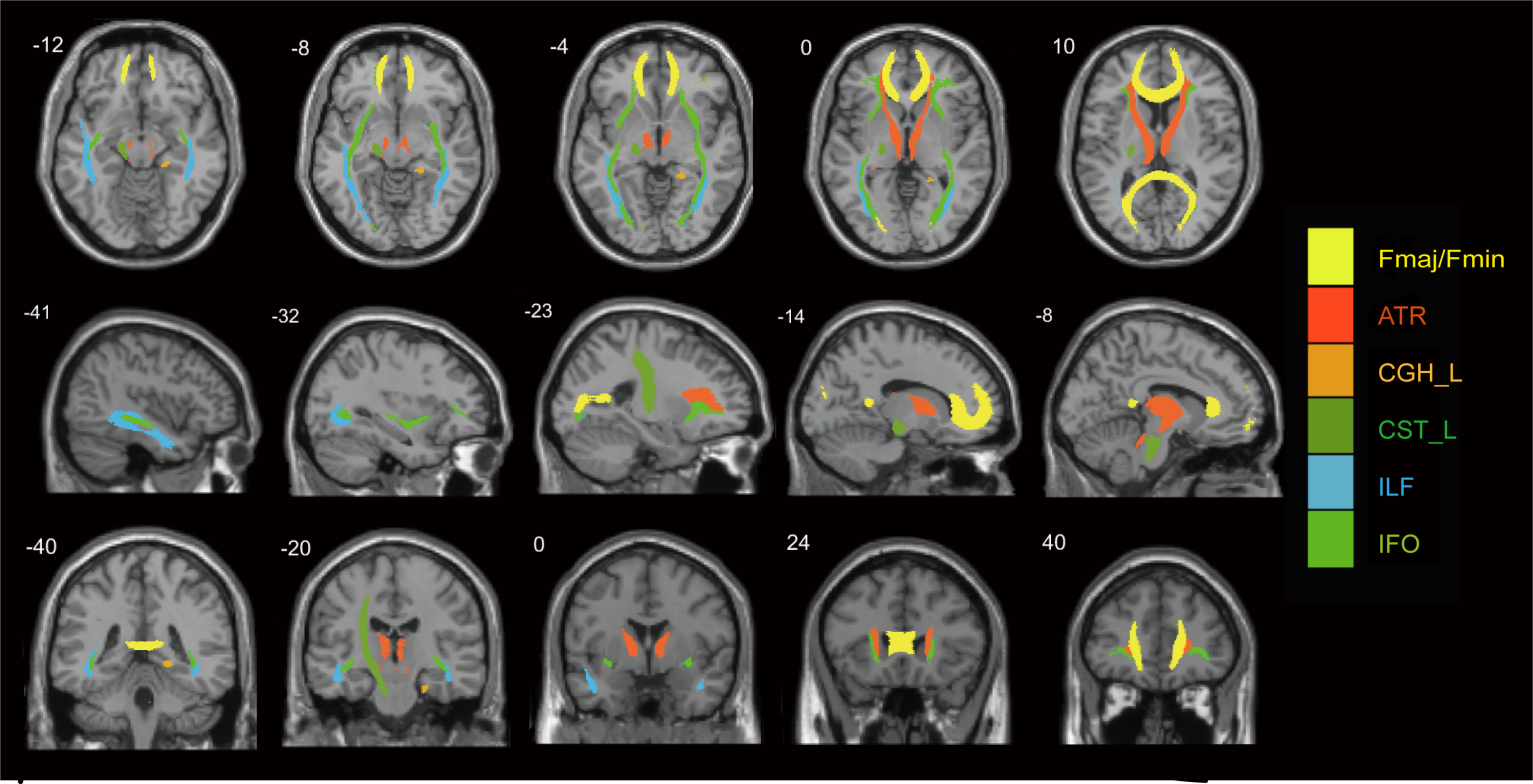
The spatial distributions of the 10 important white matter fiber tracts with significant microstructural damage in patients with NMOSD, including the bilateral ATR, CST_L, CGH_L, Fmaj, Fmin, bilateral IFO and bilateral ILF. The upper left number is the number of layers on the MNI template, and the different colors represent different fiber tracts. ATR, anterior thalamic radiation; CST, corticospinal tract; CGH, cingulum (hippocampus); Fmaj, forceps major; Fmin, forceps minor; IFO, inferior fronto-occipital fasciculus; ILF, inferior longitudinal fasciculus; L, left.

To be more representative, the 10 WM fiber tracts that showed significant differences in FA, MD, and RD were included for subsequent joint analysis of DTI and EEG. These 10 tracts spanned all four WM tract types, that is, projection fibers (ATR_L/R, CST_L), association fibers (ILF_L/R, IFO_L/R), commissural fibers (Fmaj, Fmin), and limbic system fibers (CGH_R), according to the standard of the JHU white matter tractography atlas in FSL software (their spatial distribution is displayed in **Figure 2**).

### Rs-EEG indices correlated with microstructural damage to white matter tract integrity in pwNMOSD

We performed extensive analysis of the 240 rs-EEG indices to identify potential biomarkers of microstructural damage to WM fiber tracts among pwNMOSD, including the absolute power spectrum, relative power spectrum, power spectrum ratio, and functional connectivity strength (COH, iCOH, PLV, PLI, wPLI) in five frequency bands of six different brain regions.

**Figure 3:**
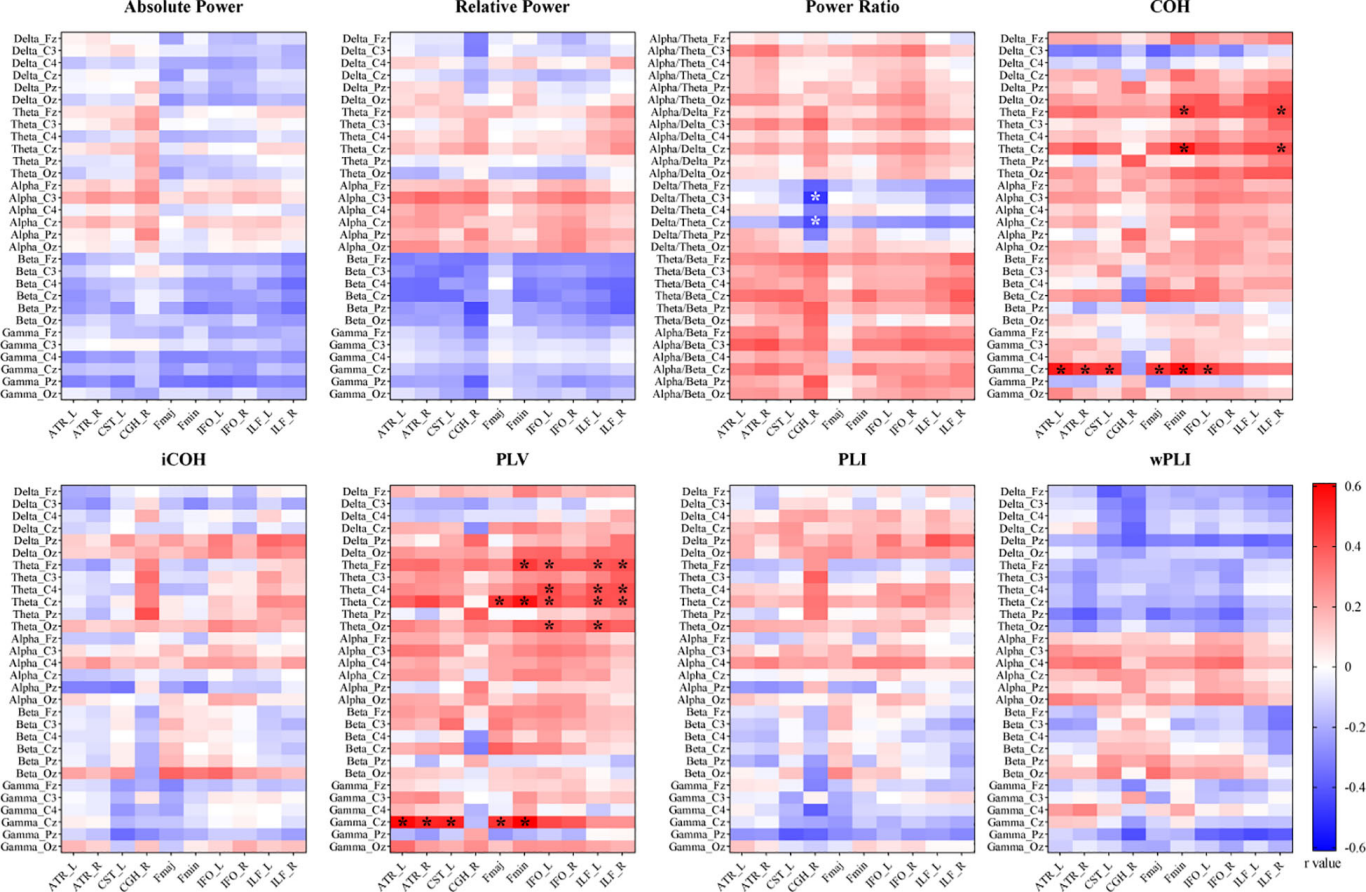
Heatmaps of the r values in the correlation analysis between the EEG metrics and the fractional anisotropy values of the impaired white matter tracts in patients with NMOSD. The correlations with statistical significance are marked with * (P<0.05 after false discovery rate correction). COH, coherence; iCOH, imaginary coherence; PLV, phase locking value; PLI, phase lag index; wPLI, weighted phase lag index; ATR, anterior thalamic radiation; CST, corticospinal tract; CGH, cingulum (hippocampus); Fmaj, forceps major; Fmin, forceps minor; IFO, inferior fronto-occipital fasciculus; ILF, inferior longitudinal fasciculus; L, left; R, right.

Since FA is the most widely used parameter of the DTI index and is sensitive to overall microstructural integrity (30, 31), the FA values of the 10 damaged WM tracts were firstly included in the subsequent DTI-EEG combined analysis as biological indicators of their integrity. **Figure 3** shows heatmaps of the r values for all the association analyses. For the rs-EEG power spectrum, only the absolute power ratio of the delta/theta of the C3 and Cz channels was significantly negatively correlated with the FA value of CGH_R (C3 r=-0.48, Cz r=-0.43). The detailed results are displayed in **Supplementary Tables 1-3.**

Relative to the power spectrum, the functional connectivity indices were significantly and positively correlated with the FA values of the impaired WM fiber tracts, especially the COH and PLV strengths in the theta/gamma frequency bands. Specifically, the theta-COH strengths (indicating the COH whole-brain mean strengths in the theta frequency band) of both the Fz and Cz channels were significantly and positively correlated with the FA values of the Fmin and ILF_R tracts (r = 0.43-0.55). Moreover, the gamma-COH strengths of the Cz channel were significantly and positively correlated with the FA values of multiple WM tracts, including ATR_L (r=0.55), ATR_R (r=0.48), CST_L (r=0.49), Fmaj (r=0.48), Fmin (r=0.57), and IFO_L (r=0.47). Similar to COH, the theta/gamma-PLV strengths had similar findings during the correlation analysis. Specifically, the theta-PLV strengths of Fz/C4/Cz/Oz were significantly correlated with the FA values of Fmaj, Fmin, IFO_L, and the bilateral ILF (r = 0.40–0.56), and the gamma-PLV strength of the Cz channel was significantly correlated with the FA values of multiple WM tracts, including ATR_L (r=0.59), ATR_R (r=0.52), CST_L (r=0.53), Fmaj (r=0.49), and Fmin (r=0.61). The detailed statistical results are displayed in **Supplementary Table 4-8**.

Also, we conducted the correlation analysis between the 240 rs-EEG indices and the mean MD/RD values of the impaired WM tracts. The gamma-PLV strength of the Cz channel was also found to be significantly correlated with the MD and RD values of multiple WM tracts, including ATR_L (r=-0.53 for MD, r=-0.54 for RD), Fmin (r=0.55 for RD), IFO_L (r=-0.54 for MD, r=-0.57 for RD), and IFO_R (r=-0.60 for MD, r=-0.53 for RD). The theta/beta power ratio of the Cz channel was found to be correlated with the mean RD values of ATR_L (r=-0.48). The heatmaps of r values and detailed statistical results could be found in the **Supplementary Materials 2** and **3**.

### Correlations between the identified rs-EEG indices and the clinical characteristics of pwNMOSD

We further analyzed the associations between disease characteristics and the rs-EEG indices that were found to be biomarkers for WM microstructural damage in pwNMOSD to further explore the relationships between these markers and the development of NMOSD.

First, we analyzed whether there were differences in these rs-EEG indices between the subgroups of pwNMOSD who were AQP4 antibody positive or negative, between patients with different disease subtypes, and between patients with different treatment strategies. The results revealed no significant differences in the rs-EEG indices among these subgroups. The detailed statistical results are displayed in **Supplementary Table 9**.

**Figure 4:**
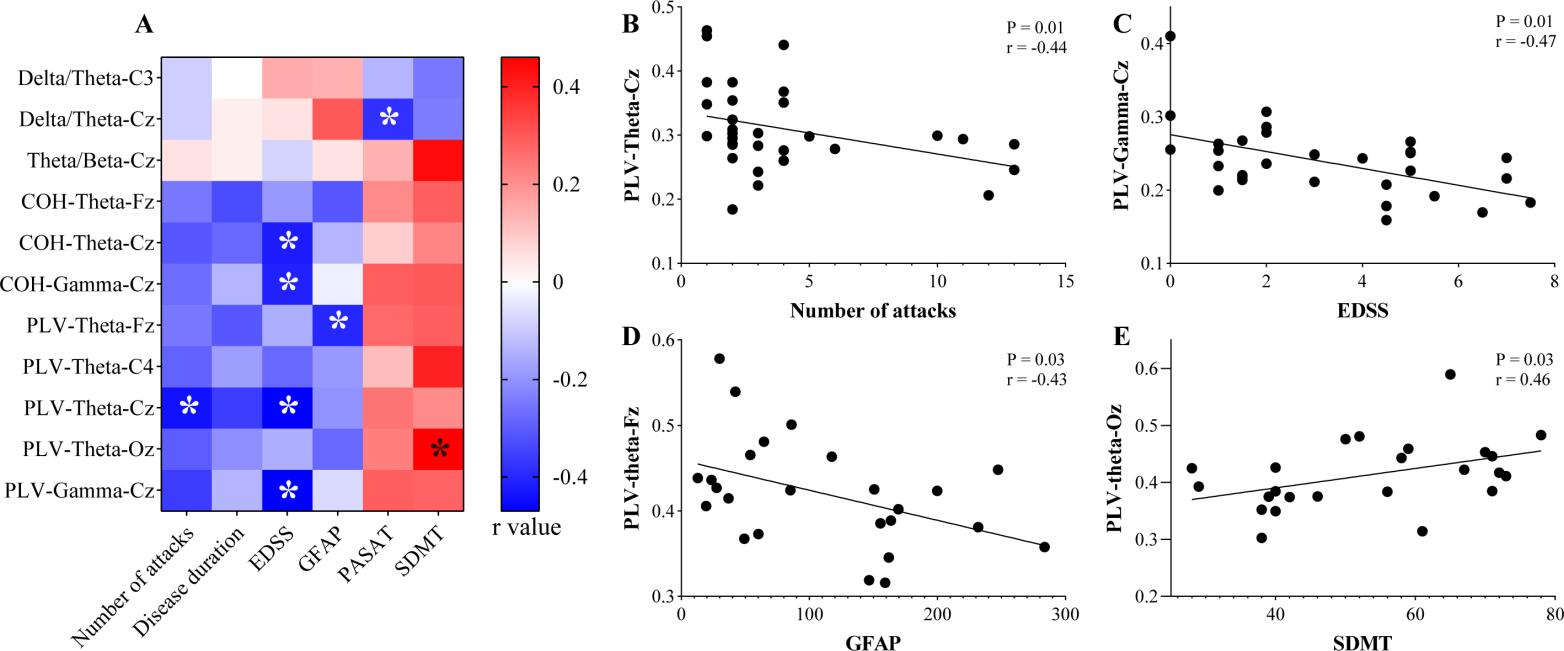
Correlations between the recognized EEG indices and clinical characteristics/cognitive functions. **(A)** Heatmaps of the r values in the correlation analysis between the EEG indices and the clinical characteristics/cognitive functions. The correlations with statistical significance are marked with *. **(B-E)** The scatter diagrams display the correlations between the recognized EEG indices and the clinical characteristics/cognitive functions, showing only the correlations with the maximum correlation coefficient (the absolute value of r). COH, coherence; PLV, phase locking value; EDSS, expanded disability status scale; GFAP, glial fibrillary acidic protein; PASAT, paced auditory serial addition test; SDMT, symbol digit modalities test.

Second, we analyzed the correlations between the identified rs-EEG indicators and other quantitative clinical features (with age as a covariate), including the number of attacks, disease duration, EDSS score, and serum GFAP concentration. **Figure 4A** shows these statistics in the form of a heatmap (r values of the correlation analysis). The results revealed that the theta-PLV strength of the Cz channel was negatively correlated with the number of disease attacks (r=-0.44), and both the theta/gamma-PLV strength and the theta/gamma-COH strength of the Cz channel were negatively correlated with the EDSS score (r=-0.41~-0.47). The theta-PLV strength of the Fz channel was negatively correlated with the serum GFAP level (r=-0.43). The scatter plots in **Figures 4B–E** show the relationships with the maximum correlation coefficient (the absolute value of r) between the rs-EEG indices and clinical characteristics. The detailed statistical results are displayed in **Supplementary Table 10**.

### Correlations between the EEG biomarkers and cognitive function in pwNMOSD

Since previous studies have suggested that cognitive impairments among pwNMOSD are correlated with WM microstructural damage, we further explored whether the identified rs-EEG biomarkers could indicate the cognitive function of pwNMOSD. Age and education level were set as covariates during the correlation analysis. Two of the identified EEG biomarkers were found to be significantly and positively correlated with executive function (SDMT scores), that is the theta/beta power ratio of the Cz channel (r=0.44) and the theta-PLV strength of the Oz channe (r=0.46, **Figure 4E**). None were significantly correlated with auditory attention function (PASAT score). The statistical results are presented in the form of a heatmap in **Figures 3A**, and the detailed statistics are displayed in **Supplementary Table 11**.

## Discussion

Given the widespread presence of WM microstructural damage and its impact on brain function, long-term monitoring of the severity of WM microstructural damage in pwNMOSD is essential. This study aimed to explore the potential of EEG as an easily accessible and reliable method for monitoring the extent of WM microstructural damage in pwNMOSD. By combining DTI and EEG, we found that extensive WM microstructural damage was present among pwNMOSD and that this microstructural damage affected mainly the functional connectivity indices as measured by rs-EEG. The PLV and COH strengths in the theta and gamma bands were strongly correlated with the integrity of WM tracts with occult microstructural damage. Moreover, different patterns were found in the associations between WM fiber integrity and EEG indices in the theta and gamma bands. The subsequent analysis further indicated that the identified rs-EEG indices were related to patients’ disease characteristics (number of attacks, EDSS score, and serum GFAP level) and cognitive functions (SDMT score).

WM in the human brain has a complex anatomy and includes 20 main tracts; these tracts can be reliably segmented via DTI (25). In this study, we evaluated the integrity of 20 main WM tracts in pwNMOSD and found that, compared with HCs, there was widespread damage to 17 WM tracts among pwNMOSD. Furthermore, our results also indicated that the lower FA was driven by greater RD, rather than AD, in pwNMOSD. Both FA and MD are sensitive to increases in cellularity, water content, and overall microstructural architecture, whereas AD and RD indicate the extent of axon integrity and myelin integrity, respectively (32). Our results suggested that WM damage in pwNMOSD was closely related to demyelination rather than to axonal degeneration, which was consistent with previous DTI studies (6, 33–35). The pattern of WM tract damage identified in this study was highly consistent with previous DTI studies in pwNMOSD (6–10), providing support for the universality of the identified EEG biomarkers of WM microstructural damage.

The correlation analysis of DTI and EEG data in this study revealed that impairments in WM tracts mainly impaired the functional connectivity strength of resting brain activity. EEG functional connectivity enables the investigation of phase synchrony between brain regions, is associated with neural communication and information transfer (36), and has been shown to reflect the organization of brain networks (20). Previous combined DTI and functional magnetic resonance imaging (fMRI) studies have revealed the impact of WM microstructural integrity on functional connectivity within or between brain networks in healthy adults and patients with multiple sclerosis (MS) (37, 38). Abnormalities in the brain networks of pwNMOSD have also been revealed in previous fMRI studies (12, 39, 40), while our study suggested that WM microstructural damage might be one of the underlying causes.

The five functional connectivity indicators examined in this study can be divided into two major categories, i.e., volume conduction-corrected phase-based measures (PLI, wPLI, and iCOH) and amplitude or more mixed connectivity-based measures (COH, PLV). Phase and amplitude-based functional connectivity measures could capture different aspects of different patho- or neurophysiological processes. Our study revealed that COH and the PLV could better reflect WM microstructural damage. This is consistent with the opinion proposed by Engel and colleagues that amplitude-based connectivity has a close relationship with the structural network and is relatively robust against state changes, whereas phased-based connectivity appeared to be less related to the structural network and showed a stronger state dependence (41). However, volume conduction-corrected phase-based measures are expected to be robust against artifacts and volume conduction and have greater reproducibility than uncorrected measures (42). The functional connectivity index used in this study was the whole-brain average of the site, which may reduce the effect of volume conduction. Nevertheless, studies with larger samples are needed to determine the stability and reproducibility of these indices.

Among the EEG indices in five different frequency bands, the potential indicators of WM microstructural damage focused on the functional connectivity indices (COH/PLV) in the theta and gamma frequency bands. Interestingly, we found that different association patterns existed between the EEG indices in different frequency bands and the integrity of WM fibers. Specifically, the identified theta-PLV indices were widely distributed (including the frontal, central, and occipital regions) and closely related to the integrity of the association fibers (IFO, ILF) and the commissural fibers (Fmaj, Fmin), whereas the identified gamma-PLV indices were centrally distributed in the central region and significantly associated with the integrity of the projection fibers (ATR, CST). Moreover, none of the EEG indices were found to be correlated with limbic system fibers (CGHs), probably because the scalp EEG we used has a methodological limitation with respect to identifying activity alterations from deep regions of the brain. The identified EEG indices of COH patients showed similar patterns to those of PLV patients. The different association patterns of WM fiber integrity and the EEG indices in different frequency bands observed herein could help us not only identify the individualized injury patterns of WM fibers according to the EEG indices of pwNMOSD but also gain a deeper understanding of the pathophysiological effects of WM fiber microstructural damage.

The correlations between the integrity of WM tracts and the functional connectivity in the theta band that we found might be partially explained by the correlations between theta activity and default mode network (DMN) activity, which has been suggested by resting-state fMRI studies (43). The DMN is an important resting-state functional network, and its functional connectivity has been shown to significantly decrease in pwNMOSD (39, 40). The cingulate tracts, the corpus callosum and the commissural fibers are suggested to be the main white matter areas that form structural connections within the functional DMN (44). The longitudinal fasciculus connecting the anterior-posterior brain regions is partially located in the distribution of the DMN. Previous DTI-EEG studies in thalamic stroke patients also revealed that the bilateral longitudinal fasciculus, cingulum, and corpus callosum were the main areas that were correlated with theta band power values (19). Our study suggested that the functional connectivity indices of the theta frequency band in pwNMOSD might indicate the structure and functional status of the DMN, which could be validated by future studies combining EEG and fMRI measures.

The functional connectivity in the gamma band was found to be correlated with the integrity of projection fibers, such as those in the CST. Inhibitory interneurons and pyramidal cells interact to produce EEG oscillations in the gamma band (45). The gamma band is known to have a significant effect on movement and walking; moreover, the gamma frequency of transcranial alternating current stimulation (tACS) is commonly used to improve motor learning (46). Our study indicates that gamma frequency band functional connectivity in the central region might be used to monitor the extent of microstructural damage to projection fibers in pwNMOSD and might also provide an intervention strategy for neurorehabilitation.

By analyzing the correlation between the clinical characteristics and the EEG indicators of WM microstructural damage, we found that the EEG indicators were not different in pwNMOSD with different disease types, AQP4 statuses, or different treatment strategies; moreover, they were not affected by disease duration, which suggested that WM microstructural damage in pwNMOSD does not accumulate over the course of disease. This result was consistent with a previous DTI study (7). More attacks and more severe disability were associated with lower levels of EEG functional connectivity indicators, which might be explained by damage to WM microstructures induced by Wallerian degeneration and retrograde axonal degeneration after acute myelitis or neuritis optica. This was the first study to explore the relationships between EEG indicators and the level of serum GFAP, which is significantly increased in pwNMOSD and is regarded as a biomarker of disease activity and disability (47, 48). We found that the higher the serum GFAP level was, the lower the functional connectivity in the theta frequency band in the frontal region was, demonstrating the correlations between the identified EEG index and the pathophysiological changes in NMOSD patients.

Previous DTI studies have offered structural evidence of the impact of WM microstructural damage on cognitive function in pwNMOSD (6, 8, 10, 33), especially executive function, as evaluated by the SDMT score (9). Our study revealed that the PLV in the theta band of the occipital region, which was correlated with the integrity of WM tracts, was positively correlated with executive function (SDMT scores) in pwNMOSD. The EEG synchrony index (PLV) has been found to mediate the relationship between WM microstructure and cognitive performance in older adults (49). Our study revealed a similar role of PLV strength in pwNMOSD, indicating functional evidence of the effects of WM microstructural damage on cognitive function and providing deeper insight into the mechanism of cognitive impairment in pwNMOSD.

This study had several limitations. Although this study revealed the functional biomarkers (rs-EEG indices) of microstructural damage in the NAWM among pwNMOSD for the first time, this was a cross-sectional study in a single center with a relatively small sample size. Thus, we failed to explore the causal relationship between EEG indices’ alterations and WM microstructural damage; meanwhile, although age was controlled as a covariate in the statistical analysis, the potential influence of other mediating factors was not completely excluded. EEG data were acquired from 32 electrodes rather than 64 or 128 electrodes. The EEG source localization method was not used to perform a more accurate structural correspondence analysis between the EEG signals and WM fiber tracts. However, since this study focused on the relationship between the integrity of the WM fiber tracts and overall EEG activity, exact structural matching was not necessary. Moreover, EEG recording with 32 electrodes is more operable in future routine clinical practice. Owing to the experimental conditions, the serum GFAP levels were measured via ELISA rather than via more sensitive single-molecule array assays. Since the purpose of this study was to find EEG indicators that could indicate the severity of white matter microstructural damage in NMOSD patients, the EEG data of HC was not included, which limited the judgment of whether the EEG indicators were disease-specific alternation or not. Other EEG indices (e.g., advanced functional connectivity network properties) should also be explored in future studies to evaluate their value in characterizing white matter microstructural damage in pwNMOSD.

As the first exploratory study, this study demonstrated the value of EEG in monitoring WM microstructural damage in pwNMOSD, however, to achieve clinical application of EEG, future research needs to determine the optimal thresholds of these EEG metrics for predicting disease progression in larger prospective cohorts (including HC and patients with other central nervous system demyelinating diseases). Simultaneously, longitudinal studies should validate the correlation between these metrics’ temporal changes and the progression of WM damage. Additionally, future studies urgently require validation of these findings in multicenter cohorts across different ethnicities and regions to confirm their cross-population applicability, which is also a prerequisite for their clinical implementation.

## Conclusion

The results of our study revealed that the functional connectivity indicators of rs-EEG are important biological indicators that can reflect pathological progression and functional status alterations related to WM microstructural damage, especially the COH and PLV strengths in the theta and gamma frequency bands. Moreover, different patterns associated with WM fiber integrity and EEG indices in different frequency bands were identified in this study, which could help predict individualized injury patterns in WM fibers according to EEG indices in patients. Furthermore, the PLV strengths of key regions were strongly correlated with disease severity, serum biomarkers and cognitive functions, and may be used as important biomarkers for monitoring brain function and disease severity in patients. Our study suggests the potential for EEG to be used during routine clinical processes as a surrogate for neuroimaging in the disease monitoring of pwNMOSD, especially in the assessment of WM microstructural damage.

## Data Availability

The raw data supporting the conclusions of this article will be made available by the authors, without undue reservation.

## Supplementary material

SUPPLEMENTARY FILE 1 Correlation analysis between the resting-state EEG power spectrum indices(absolute power, relative power, and absolute power ratio) and the mean FA values of impaired WM tracts in patients with NMOSD.

SUPPLEMENTARY FILE 2 The heatmap of r values and detailed statistical results of the correlation analysis between rs-EEG indices and the mean MD values of impaired WM tracts.

SUPPLEMENTARY FILE 3 The heatmap of r values and detailed statistical results of the correlation analysis between rs-EEG indices and the mean RD values of impaired WM tracts.

## References

[1] Wingerchuk DM Banwell B Bennett JL Cabre P Carroll W Chitnis T . International consensus diagnostic criteria for neuromyelitis optica spectrum disorders. Neurology. (2015) 85:177–89. doi: 10.1212/WNL.0000000000001729, PMID: 26092914 PMC4515040

[2] Weinshenker BG Wingerchuk DM . Neuromyelitis spectrum disorders. Mayo Clin Proc. (2017) 92:663–79. doi: 10.1016/j.mayocp.2016.12.014, PMID: 28385199

[3] Etemadifar M Nasr Z Khalili B Taherioun M Vosoughi R . Epidemiology of neuromyelitis optica in the world: a systematic review and meta-analysis. Multiple Sclerosis Int. (2015) 2015:174720. doi: 10.1155/2015/174720, PMID: 25973275 PMC4417948

[4] Hor JY Lim TT Chia YK Ching YM Cheah CF Tan K . Prevalence of neuromyelitis optica spectrum disorder in the multi-ethnic Penang Island, Malaysia, and a review of worldwide prevalence. Multiple Sclerosis Relat Disord. (2018) 19:20–4. doi: 10.1016/j.msard.2017.10.015, PMID: 29100047

[5] Kessler RA Mealy MA Levy M . Treatment of neuromyelitis optica spectrum disorder: acute, preventive, and symptomatic. Curr Treat Options Neurol. (2016) 18:2. doi: 10.1007/s11940-015-0387-9, PMID: 26705758 PMC4807395

[6] Chen X Roberts N Zheng Q Peng Y Han Y Luo Q . Comparison of diffusion tensor imaging (DTI) tissue characterization parameters in white matter tracts of patients with multiple sclerosis (MS) and neuromyelitis optica spectrum disorder (NMOSD). Eur Radiol. (2024) 34:5263–75. doi: 10.1007/s00330-023-10550-1, PMID: 38175221

[7] Chen X Roberts N Zheng Q Peng Y Han Y Luo Q . Progressive brain microstructural damage in patients with multiple sclerosis but not in patients with neuromyelitis optica spectrum disorder: A cross-sectional and follow-up tract-based spatial statistics study. Multiple Sclerosis Relat Disord. (2021) 55:103178. doi: 10.1016/j.msard.2021.103178, PMID: 34384989

[8] Kim SH Park EY Park B Hyun JW Park NY Joung A . Multimodal magnetic resonance imaging in relation to cognitive impairment in neuromyelitis optica spectrum disorder. Sci Rep. (2017) 7:9180. doi: 10.1038/s41598-017-08889-9, PMID: 28835657 PMC5569012

[9] Cacciaguerra L Rocca MA Storelli L Radaelli M Filippi M . Mapping white matter damage distribution in neuromyelitis optica spectrum disorders with a multimodal MRI approach. Multiple Sclerosis (Houndmills Basingstoke England). (2021) 27:841–54. doi: 10.1177/1352458520941493, PMID: 32672089

[10] Yan Z Wang X Zhu Q Shi Z Chen X Han Y . Alterations in white matter fiber tracts characterized by automated fiber-tract quantification and their correlations with cognitive impairment in neuromyelitis optica spectrum disorder patients. Front Neurosci. (2022) 16:904309. doi: 10.3389/fnins.2022.904309, PMID: 35844220 PMC9283762

[11] He D Wu Q Chen X Zhao D Gong Q Zhou H . Cognitive impairment and whole brain diffusion in patients with neuromyelitis optica after acute relapse. Brain Cognit. (2011) 77:80–8. doi: 10.1016/j.bandc.2011.05.007, PMID: 21723024

[12] Liu Y Xiong H Li X Zhang D Yang C Yu J . Abnormal baseline brain activity in neuromyelitis optica patients without brain lesion detected by resting-state functional magnetic resonance imaging. Neuropsychiatr Dis Treat. (2020) 16:71–9. doi: 10.2147/NDT.S232924, PMID: 32021200 PMC6955618

[13] Wen X Wu X Liu J Li K Yao L . Abnormal baseline brain activity in non-depressed Parkinson’s disease and depressed Parkinson’s disease: a resting-state functional magnetic resonance imaging study. PloS One. (2013) 8:e63691. doi: 10.1371/journal.pone.0063691, PMID: 23717467 PMC3661727

[14] Khadka S Meda SA Stevens MC Glahn DC Calhoun VD Sweeney JA . Is aberrant functional connectivity a psychosis endophenotype? A resting state functional magnetic resonance imaging study. Biol Psychiatry. (2013) 74:458–66. doi: 10.1016/j.biopsych.2013.04.024, PMID: 23746539 PMC3752322

[15] Wilde EA Goodrich-Hunsaker NJ Ware AL Taylor BA Biekman BD Hunter JV . Diffusion tensor imaging indicators of white matter injury are correlated with a multimodal electroencephalography-based biomarker in slow recovering, concussed collegiate athletes. J Neurotrauma. (2020) 37:2093–101. doi: 10.1089/neu.2018.6365, PMID: 31931657

[16] Wang C Costanzo ME Rapp PE Darmon D Nathan DE Bashirelahi K . Disrupted gamma synchrony after mild traumatic brain injury and its correlation with white matter abnormality. Front Neurol. (2017) 8:571. doi: 10.3389/fneur.2017.00571, PMID: 29163337 PMC5670344

[17] Sponheim SR McGuire KA Kang SS Davenport ND Aviyente S Bernat EM . Evidence of disrupted functional connectivity in the brain after combat-related blast injury. NeuroImage. (2011) 54 Suppl 1:S21–9. doi: 10.1016/j.neuroimage.2010.09.007, PMID: 20851190

[18] Guggisberg AG Nicolo P Cohen LG Schnider A Buch ER . Longitudinal structural and functional differences between proportional and poor motor recovery after stroke. Neurorehabilitation Neural Repair. (2017) 31:1029–41. doi: 10.1177/1545968317740634, PMID: 29130824 PMC6368856

[19] Duru AD Duru DG Yumerhodzha S Bebek N . Analysis of correlation between white matter changes and functional responses in thalamic stroke: a DTI & EEG study. Brain Imaging Behav. (2016) 10:424–36. doi: 10.1007/s11682-015-9397-1, PMID: 25957181

[20] Deco G Jirsa VK McIntosh AR . Emerging concepts for the dynamical organization of resting-state activity in the brain. Nat Rev Neurosci. (2011) 12:43–56. doi: 10.1038/nrn2961, PMID: 21170073

[21] Sanchez E El-Khatib H Arbour C Bedetti C Blais H Marcotte K . Brain white matter damage and its association with neuronal synchrony during sleep. Brain: J Neurol. (2019) 142:674–87. doi: 10.1093/brain/awy348, PMID: 30698667 PMC6391600

[22] Yang L Xu C Qin Y Chen K Xie Y Zhou X . Exploring resting-state EEG oscillations in patients with Neuromyelitis Optica Spectrum Disorder. Brain Res Bull. (2024) 208:110900. doi: 10.1016/j.brainresbull.2024.110900, PMID: 38364986

[23] Kurtzke JF . Rating neurologic impairment in multiple sclerosis: an expanded disability status scale (EDSS). Neurology. (1983) 33:1444–52. doi: 10.1212/WNL.33.11.1444, PMID: 6685237

[24] Cui Z Zhong S Xu P He Y Gong G . PANDA: a pipeline toolbox for analyzing brain diffusion images. Front Hum Neurosci. (2013) 7:42. doi: 10.3389/fnhum.2013.00042, PMID: 23439846 PMC3578208

[25] Wakana S Caprihan A Panzenboeck MM Fallon JH Perry M Gollub RL . Reproducibility of quantitative tractography methods applied to cerebral white matter. NeuroImage. (2007) 36:630–44. doi: 10.1016/j.neuroimage.2007.02.049, PMID: 17481925 PMC2350213

[26] Delorme A Makeig S . EEGLAB: an open source toolbox for analysis of single-trial EEG dynamics including independent component analysis. J Neurosci Methods. (2004) 134:9–21. doi: 10.1016/j.jneumeth.2003.10.009, PMID: 15102499

[27] Yao D . A method to standardize a reference of scalp EEG recordings to a point at infinity. Physiol Meas. (2001) 22:693–711. doi: 10.1088/0967-3334/22/4/305, PMID: 11761077

[28] Niso G Bruña R Pereda E Gutiérrez R Bajo R Maestú F . HERMES: towards an integrated toolbox to characterize functional and effective brain connectivity. Neuroinformatics. (2013) 11:405–34. doi: 10.1007/s12021-013-9186-1, PMID: 23812847

[29] Boca SM Leek JT . A direct approach to estimating false discovery rates conditional on covariates. PeerJ. (2018) 6:e6035. doi: 10.7717/peerj.6035, PMID: 30581661 PMC6292380

[30] Inglese M Bester M . Diffusion imaging in multiple sclerosis: research and clinical implications. NMR Biomed. (2010) 23:865–72. doi: 10.1002/nbm.1515, PMID: 20882528 PMC3071990

[31] Assaf Y Pasternak O . Diffusion tensor imaging (DTI)-based white matter mapping in brain research: a review. J Mol Neurosci: MN. (2008) 34:51–61. doi: 10.1007/s12031-007-0029-0, PMID: 18157658

[32] Tu TW Williams RA Lescher JD Jikaria N Turtzo LC Frank JA . Radiological-pathological correlation of diffusion tensor and magnetization transfer imaging in a closed head traumatic brain injury model. Ann Neurol. (2016) 79:907–20. doi: 10.1002/ana.24641, PMID: 27230970 PMC4887193

[33] Kim SH Kwak K Hyun JW Joung A Lee SH Choi YH . Diffusion tensor imaging of normal-appearing white matter in patients with neuromyelitis optica spectrum disorder and multiple sclerosis. Eur J Neurol. (2017) 24:966–73. doi: 10.1111/ene.13321, PMID: 28643955

[34] Rueda Lopes FC Doring T Martins C Cabral FC Malfetano FR Pereira VC . The role of demyelination in neuromyelitis optica damage: diffusion-tensor MR imaging study. Radiology. (2012) 263:235–42. doi: 10.1148/radiol.12111241, PMID: 22438446

[35] Pardini M Bonzano L Bergamino M Bommarito G Feraco P Murugavel A . Cingulum bundle alterations underlie subjective fatigue in multiple sclerosis. Multiple Sclerosis (Houndmills Basingstoke England). (2015) 21:442–7. doi: 10.1177/1352458514546791, PMID: 25145692

[36] Fell J Axmacher N . The role of phase synchronization in memory processes. Nat Rev Neurosci. (2011) 12:105–18. doi: 10.1038/nrn2979, PMID: 21248789

[37] Brown CA Hakun JG Zhu Z Johnson NF Gold BT . White matter microstructure contributes to age-related declines in task-induced deactivation of the default mode network. Front Aging Neurosci. (2015) 7:194. doi: 10.3389/fnagi.2015.00194, PMID: 26500549 PMC4598480

[38] Teipel SJ Bokde AL Meindl T Amaro E Jr. Soldner J Reiser MF . White matter microstructure underlying default mode network connectivity in the human brain. NeuroImage. (2010) 49:2021–32. doi: 10.1016/j.neuroimage.2009.10.067, PMID: 19878723

[39] Yang L Qin Y Chen K Xu C Peng M Tan S . The role of basal ganglia network in neural plasticity in neuromyelitis optica spectrum disorder with myelitis. Multiple Sclerosis Relat Disord. (2022) 68:104170. doi: 10.1016/j.msard.2022.104170, PMID: 36113277

[40] Rocca MA Savoldi F Valsasina P Radaelli M Preziosa P Comi G . Cross-modal plasticity among sensory networks in neuromyelitis optica spectrum disorders. Multiple Sclerosis (Houndmills Basingstoke England). (2019) 25:968–79. doi: 10.1177/1352458518778008, PMID: 29771186

[41] Engel AK Gerloff C Hilgetag CC Nolte G . Intrinsic coupling modes: multiscale interactions in ongoing brain activity. Neuron. (2013) 80:867–86. doi: 10.1016/j.neuron.2013.09.038, PMID: 24267648

[42] Duan W Chen X Wang YJ Zhao W Yuan H Lei X . Reproducibility of power spectrum, functional connectivity and network construction in resting-state EEG. J Neurosci Methods. (2021) 348:108985. doi: 10.1016/j.jneumeth.2020.108985, PMID: 33164816

[43] Scheeringa R Bastiaansen MC Petersson KM Oostenveld R Norris DG Hagoort P . Frontal theta EEG activity correlates negatively with the default mode network in resting state. Int J Psychophysiol: Off J Int Organ Psychophysiol. (2008) 67:242–51. doi: 10.1016/j.ijpsycho.2007.05.017, PMID: 17707538

[44] Horn A Ostwald D Reisert M Blankenburg F . The structural-functional connectome and the default mode network of the human brain. NeuroImage. (2014) 102 Pt 1:142–51. doi: 10.1016/j.neuroimage.2013.09.069, PMID: 24099851

[45] Buzsáki G Wang XJ . Mechanisms of gamma oscillations. Annu Rev Neurosci. (2012) 35:203–25. doi: 10.1146/annurev-neuro-062111-150444, PMID: 22443509 PMC4049541

[46] Feng T Zhang L Wu Y Tang L Chen X Li Y . Exploring the therapeutic effects and mechanisms of transcranial alternating current stimulation on improving walking ability in stroke patients via modulating cerebellar gamma frequency band-a narrative review. Cerebellum (London England). (2024) 23:1593–603. doi: 10.1007/s12311-023-01632-3, PMID: 37962773 PMC11269344

[47] Watanabe M Nakamura Y Michalak Z Isobe N Barro C Leppert D . Serum GFAP and neurofilament light as biomarkers of disease activity and disability in NMOSD. Neurology. (2019) 93:e1299–e311. doi: 10.1212/WNL.0000000000008160, PMID: 31471502

[48] Aktas O Smith MA Rees WA Bennett JL She D Katz E . Serum glial fibrillary acidic protein: A neuromyelitis optica spectrum disorder biomarker. Ann Neurol. (2021) 89:895–910. doi: 10.1002/ana.26067, PMID: 33724534 PMC8252046

[49] Hinault T Kraut M Bakker A Dagher A Courtney SM . Disrupted neural synchrony mediates the relationship between white matter integrity and cognitive performance in older adults. Cereb Cortex (New York NY: 1991). (2020) 30:5570–82. doi: 10.1093/cercor/bhaa141, PMID: 32483609

