# Correction: EEG biomarkers of microstructural damage in normal-appearing white matter among patients with neuromyelitis optica spectrum disorder: a DTI-EEG combined study

**DOI:** 10.3389/fimmu.2026.1834718

**Published:** 2026-04-13

**Authors:** Frontiers Production Office

**Affiliations:** Frontiers Media SA, Lausanne, Switzerland

**Keywords:** diffusion tensor imaging, functional connectivity, neuromyelitis optica, resting-state EEG, white matter microstructural damage

In the abstract, subsection **Discussion** was erroneously omitted. This has been corrected to read:

“**Discussion:** The functional connectivity of rs-EEG may serve as important biomarkers of WM microstructural damage in NMOSD.”

The original version of this article has been updated.

