# Supplementary material for: EEG biomarkers of microstructural damage in normal-appearing white matter among patients with neuromyelitis optica spectrum disorder: A DTI-EEG combined study
Source: Front Immunol. 2026 Feb 25;17:1676066. doi: 10.3389/fimmu.2026.1676066 (PMC12975549; doi:10.3389/fimmu.2026.1676066)
Supplement: SUPPLEMENTARY FILE 1 — Correlation analysis between the resting-state EEG power spectrum indices(absolute power, relative power, and absolute power ratio) and the mean FA values of impaired WM tracts in patients with NMOSD. [file Supplementaryfile1.docx]

**Supplementary material**

**Methods**

**Magnetic Resonance Image Acquisition**

All the participants underwent MRI scans, which were performed with a 3T MRI scanner (GE DISCOVERY MR750) via a 32-channel head coil.

High-resolution T1-weighted images were acquired using a 3-dimensional T1-weighted fast spoiled gradient echo sequence (echo time (TE) = 1.972 ms, repetition time (TR) = 6.0 ms, inversion time (TI) = 450 ms, flip angle (FA) = 9°, matrix = 256 × 256, field of view (FOV) = 25.6 cm × 25.6 cm, slice thickness = 1 mm, no gap, 156 slices). T2-weighted fluid-attenuated inversion recovery images were also acquired to exclude participants with significant brain lesions (TR = 8.4 msec, FA = 111°, matrix = 256 × 256, FOV = 24 cm × 24 cm, slice thickness/gap = 5 mm/1.5 mm). Additionally, diffusion-weighted images were obtained using single-shot echo planar imaging sequences. One shell at b-value 1000 s/mm^2^ along 30 non-collinear directions and 3 b = 0 volumes were acquired. Parameters for the sequence comprised plane orientation=axial, TR = 8500 ms, TE = 63 ms, matrix = 128 × 128, FOV = 256mm × 256mm, thickness = 2 mm without a gap.

**Supplementary table S1.** **The correlation analysis between the resting-state EEG absolute power and the mean FA values of impaired WM tracts in patients with NMOSD (P value and r value).** Pearson correlation analysis or Spearman correlation analysis were used according to the normality distributions of the data, with age as covariant. P<0.05 with false discovery rate corrected was considered significant, marked with **bold font**.

|  | ATR_L | | ATR_R | | CST_L | | CGH_R | | Fmaj | | Fmin | | IFO_L | | IFO_R | | ILF_L | | ILF_R | |
| --- | --- | --- | --- | --- | --- | --- | --- | --- | --- | --- | --- | --- | --- | --- | --- | --- | --- | --- | --- | --- |
|  | P | r | P | r | P | r | P | r | P | r | P | r | P | r | P | r | P | r | P | r |
| Delta_Fz | 0.89 | 0.03 | 0.73 | 0.06 | 0.96 | -0.01 | 0.94 | -0.01 | 0.24 | -0.22 | 0.92 | -0.02 | 0.39 | -0.16 | 0.36 | -0.17 | 0.66 | -0.08 | 0.64 | -0.09 |
| Delta_C3 | 0.96 | 0.01 | 0.83 | 0.04 | 0.64 | 0.09 | 0.97 | 0.01 | 0.81 | -0.05 | 0.81 | -0.05 | 0.45 | -0.14 | 0.50 | -0.13 | 0.54 | -0.12 | 0.31 | -0.19 |
| Delta_C4 | 0.41 | -0.15 | 0.63 | -0.09 | 0.54 | -0.12 | 0.75 | -0.06 | 0.27 | -0.20 | 0.28 | -0.20 | 0.21 | -0.23 | 0.25 | -0.21 | 0.50 | -0.13 | 0.34 | -0.18 |
| Delta_Cz | 0.88 | -0.03 | 0.93 | 0.02 | 0.96 | -0.01 | 0.96 | -0.01 | 0.17 | -0.25 | 0.80 | -0.05 | 0.35 | -0.17 | 0.37 | -0.17 | 0.75 | -0.06 | 0.73 | -0.07 |
| Delta_Pz | 0.93 | -0.02 | 0.98 | -0.01 | 1.00 | 0.00 | 0.45 | 0.14 | 0.44 | -0.14 | 0.55 | -0.11 | 0.28 | -0.20 | 0.39 | -0.16 | 0.58 | -0.10 | 0.62 | -0.09 |
| Delta_Oz | 0.53 | -0.12 | 0.76 | -0.06 | 0.62 | -0.09 | 0.78 | 0.05 | 0.15 | -0.26 | 0.36 | -0.17 | 0.22 | -0.23 | 0.24 | -0.22 | 0.41 | -0.15 | 0.36 | -0.17 |
| Theta_Fz | 0.76 | 0.06 | 0.67 | 0.08 | 0.65 | 0.08 | 0.25 | 0.21 | 0.59 | -0.10 | 0.86 | 0.03 | 1.00 | 0.00 | 0.97 | 0.01 | 0.58 | 0.10 | 0.58 | 0.10 |
| Theta_C3 | 0.97 | 0.01 | 0.81 | 0.04 | 0.52 | 0.12 | 0.19 | 0.24 | 0.94 | -0.01 | 0.98 | 0.00 | 0.80 | -0.05 | 0.84 | -0.04 | 0.84 | 0.04 | 0.94 | -0.01 |
| Theta_C4 | 0.46 | -0.14 | 0.61 | -0.10 | 0.76 | -0.06 | 0.53 | 0.12 | 0.30 | -0.19 | 0.35 | -0.17 | 0.41 | -0.15 | 0.44 | -0.14 | 0.85 | -0.04 | 0.62 | -0.09 |
| Theta_Cz | 0.78 | 0.05 | 0.64 | 0.09 | 0.50 | 0.13 | 0.28 | 0.20 | 0.76 | -0.06 | 0.71 | 0.07 | 0.97 | -0.01 | 1.00 | 0.00 | 0.59 | 0.10 | 0.65 | 0.09 |
| Theta_Pz | 0.70 | -0.07 | 0.69 | -0.07 | 0.80 | -0.05 | 0.24 | 0.22 | 0.43 | -0.15 | 0.45 | -0.14 | 0.52 | -0.12 | 0.63 | -0.09 | 0.99 | 0.00 | 0.94 | -0.01 |
| Theta_Oz | 0.47 | -0.14 | 0.76 | -0.06 | 0.74 | -0.06 | 0.37 | 0.17 | 0.23 | -0.22 | 0.51 | -0.12 | 0.41 | -0.15 | 0.50 | -0.12 | 0.59 | -0.10 | 0.75 | -0.06 |
| Alpha_Fz | 0.66 | 0.08 | 0.47 | 0.13 | 0.76 | 0.06 | 0.21 | 0.23 | 0.68 | -0.08 | 0.79 | 0.05 | 0.70 | 0.07 | 0.58 | 0.10 | 0.65 | 0.09 | 0.89 | 0.02 |
| Alpha_C3 | 0.32 | 0.18 | 0.18 | 0.25 | 0.26 | 0.21 | 0.13 | 0.28 | 0.69 | 0.07 | 0.44 | 0.15 | 0.55 | 0.11 | 0.36 | 0.17 | 0.47 | 0.14 | 0.76 | 0.06 |
| Alpha_C4 | 0.87 | -0.03 | 0.79 | 0.05 | 0.92 | -0.02 | 0.52 | 0.12 | 0.54 | -0.11 | 0.71 | -0.07 | 0.80 | -0.05 | 0.96 | -0.01 | 0.92 | -0.02 | 0.60 | -0.10 |
| Alpha_Cz | 0.47 | 0.13 | 0.31 | 0.19 | 0.52 | 0.12 | 0.31 | 0.19 | 0.99 | 0.00 | 0.50 | 0.13 | 0.60 | 0.10 | 0.53 | 0.12 | 0.47 | 0.14 | 0.70 | 0.07 |
| Alpha_Pz | 0.84 | 0.04 | 0.77 | 0.06 | 0.95 | -0.01 | 0.13 | 0.28 | 0.87 | -0.03 | 0.68 | -0.08 | 0.87 | 0.03 | 0.59 | 0.10 | 0.69 | 0.08 | 0.78 | 0.05 |
| Alpha_Oz | 0.89 | 0.02 | 0.81 | 0.05 | 0.85 | -0.04 | 0.48 | 0.13 | 0.78 | -0.05 | 0.75 | -0.06 | 0.92 | 0.02 | 0.83 | 0.04 | 0.92 | 0.02 | 0.80 | -0.05 |
| Beta_Fz | 0.20 | -0.23 | 0.41 | -0.15 | 0.48 | -0.13 | 0.89 | -0.03 | 0.45 | -0.14 | 0.23 | -0.22 | 0.22 | -0.22 | 0.23 | -0.22 | 0.22 | -0.23 | 0.09 | -0.31 |
| Beta_C3 | 0.50 | -0.13 | 0.72 | -0.07 | 0.99 | 0.00 | 0.73 | 0.07 | 0.86 | 0.03 | 0.57 | -0.11 | 0.42 | -0.15 | 0.53 | -0.12 | 0.42 | -0.15 | 0.16 | -0.26 |
| Beta_C4 | 0.22 | -0.23 | 0.40 | -0.16 | 0.44 | -0.14 | 0.68 | -0.08 | 0.44 | -0.14 | 0.20 | -0.24 | 0.19 | -0.24 | 0.38 | -0.16 | 0.14 | -0.27 | 0.05 | -0.35 |
| Beta_Cz | 0.15 | -0.26 | 0.29 | -0.20 | 0.53 | -0.12 | 0.89 | -0.03 | 0.72 | -0.07 | 0.25 | -0.21 | 0.22 | -0.23 | 0.20 | -0.23 | 0.28 | -0.20 | 0.12 | -0.28 |
| Beta_Pz | 0.19 | -0.24 | 0.32 | -0.19 | 0.18 | -0.25 | 0.86 | -0.03 | 0.27 | -0.21 | 0.07 | -0.33 | 0.11 | -0.29 | 0.20 | -0.24 | 0.14 | -0.27 | 0.06 | -0.34 |
| Beta_Oz | 0.42 | -0.15 | 0.65 | -0.09 | 0.42 | -0.15 | 0.56 | -0.11 | 0.53 | -0.12 | 0.42 | -0.15 | 0.37 | -0.17 | 0.63 | -0.09 | 0.20 | -0.24 | 0.18 | -0.25 |
| Gamma_Fz | 0.69 | -0.07 | 0.80 | -0.05 | 0.42 | -0.15 | 0.38 | -0.16 | 0.29 | -0.20 | 0.68 | -0.08 | 0.24 | -0.22 | 0.25 | -0.21 | 0.28 | -0.20 | 0.36 | -0.17 |
| Gamma_C3 | 0.86 | -0.03 | 0.99 | 0.00 | 0.92 | 0.02 | 0.91 | 0.02 | 0.67 | -0.08 | 0.77 | -0.05 | 0.50 | -0.13 | 0.64 | -0.09 | 0.48 | -0.13 | 0.34 | -0.18 |
| Gamma_C4 | 0.13 | -0.28 | 0.22 | -0.23 | 0.18 | -0.25 | 0.46 | -0.14 | 0.11 | -0.29 | 0.12 | -0.29 | 0.16 | -0.26 | 0.16 | -0.26 | 0.22 | -0.23 | 0.14 | -0.27 |
| Gamma_Cz | 0.38 | -0.16 | 0.48 | -0.13 | 0.45 | -0.14 | 0.51 | -0.12 | 0.17 | -0.25 | 0.44 | -0.14 | 0.24 | -0.22 | 0.17 | -0.25 | 0.47 | -0.14 | 0.36 | -0.17 |
| Gamma_Pz | 0.10 | -0.30 | 0.16 | -0.26 | 0.08 | -0.32 | 0.50 | -0.13 | 0.08 | -0.32 | 0.06 | -0.34 | 0.05 | -0.36 | 0.07 | -0.33 | 0.13 | -0.28 | 0.11 | -0.29 |
| Gamma_Oz | 0.83 | -0.04 | 0.72 | -0.07 | 0.33 | -0.18 | 0.27 | -0.21 | 0.67 | -0.08 | 0.67 | -0.08 | 0.50 | -0.13 | 0.78 | -0.05 | 0.36 | -0.17 | 0.34 | -0.18 |

**Supplementary table S2.** **The correlation analysis between the resting-state EEG relative power and the mean FA values of impaired WM tracts in patients with NMOSD (P value and r value).** Pearson correlation analysis or Spearman correlation analysis were used according to the normality distributions of the data, with age as covariant. P<0.05 with false discovery rate corrected was considered significant, marked with **bold font**.

|  | ATR_L | | ATR_R | | CST_L | | CGH_R | | Fmaj | | Fmin | | IFO_L | | IFO_R | | ILF_L | | ILF_R | |
| --- | --- | --- | --- | --- | --- | --- | --- | --- | --- | --- | --- | --- | --- | --- | --- | --- | --- | --- | --- | --- |
|  | P | r | P | r | P | r | P | r | P | r | P | r | P | r | P | r | P | r | P | r |
| Delta_Fz | 0.88 | -0.03 | 0.75 | -0.06 | 0.77 | -0.06 | 0.10 | -0.30 | 0.80 | -0.05 | 0.97 | 0.01 | 0.51 | -0.12 | 0.31 | -0.19 | 0.66 | -0.08 | 0.99 | 0.00 |
| Delta_C3 | 0.92 | -0.02 | 0.62 | -0.09 | 0.68 | -0.08 | 0.09 | -0.31 | 0.95 | -0.01 | 0.67 | -0.08 | 0.66 | -0.08 | 0.49 | -0.13 | 0.53 | -0.12 | 0.79 | -0.05 |
| Delta_C4 | 0.55 | 0.11 | 0.64 | 0.09 | 0.89 | 0.03 | 0.44 | -0.14 | 0.72 | 0.07 | 0.49 | 0.13 | 0.83 | 0.04 | 0.95 | 0.01 | 0.68 | 0.08 | 0.26 | 0.21 |
| Delta_Cz | 0.90 | -0.02 | 0.74 | -0.06 | 0.55 | -0.11 | 0.28 | -0.20 | 0.58 | -0.10 | 0.55 | -0.11 | 0.32 | -0.19 | 0.35 | -0.17 | 0.45 | -0.14 | 0.86 | -0.03 |
| Delta_Pz | 0.62 | 0.09 | 0.69 | 0.07 | 0.56 | 0.11 | 0.34 | -0.18 | 0.82 | 0.04 | 0.54 | 0.12 | 0.77 | -0.05 | 0.64 | -0.09 | 0.74 | -0.06 | 0.98 | 0.00 |
| Delta_Oz | 0.66 | 0.08 | 0.46 | 0.14 | 0.55 | 0.11 | 0.84 | -0.04 | 0.99 | 0.00 | 0.38 | 0.16 | 0.82 | -0.04 | 0.79 | -0.05 | 0.96 | 0.01 | 0.60 | 0.10 |
| Theta_Fz | 0.50 | 0.13 | 0.63 | 0.09 | 0.44 | 0.14 | 0.38 | 0.16 | 0.36 | 0.17 | 0.66 | 0.08 | 0.74 | 0.06 | 0.71 | 0.07 | 0.34 | 0.18 | 0.14 | 0.27 |
| Theta_C3 | 1.00 | 0.00 | 0.82 | -0.04 | 0.71 | 0.07 | 0.30 | 0.19 | 0.94 | -0.01 | 0.93 | -0.02 | 0.98 | 0.00 | 0.82 | -0.04 | 0.50 | 0.13 | 0.35 | 0.18 |
| Theta_C4 | 0.55 | 0.11 | 0.75 | 0.06 | 0.57 | 0.11 | 0.30 | 0.19 | 0.99 | 0.00 | 0.80 | 0.05 | 0.70 | 0.07 | 0.76 | 0.06 | 0.30 | 0.19 | 0.21 | 0.23 |
| Theta_Cz | 0.42 | 0.15 | 0.57 | 0.11 | 0.31 | 0.19 | 0.18 | 0.25 | 0.70 | 0.07 | 0.50 | 0.13 | 0.67 | 0.08 | 0.62 | 0.09 | 0.32 | 0.18 | 0.14 | 0.27 |
| Theta_Pz | 0.89 | -0.03 | 0.75 | -0.06 | 0.85 | 0.03 | 0.88 | 0.03 | 0.92 | -0.02 | 0.99 | 0.00 | 0.75 | -0.06 | 0.59 | -0.10 | 0.83 | 0.04 | 0.79 | 0.05 |
| Theta_Oz | 0.33 | -0.18 | 0.32 | -0.19 | 0.64 | -0.09 | 0.89 | 0.02 | 0.43 | -0.15 | 0.39 | -0.16 | 0.26 | -0.21 | 0.25 | -0.21 | 0.68 | -0.08 | 0.96 | -0.01 |
| Alpha_Fz | 0.48 | 0.13 | 0.47 | 0.14 | 0.39 | 0.16 | 0.26 | 0.21 | 0.72 | 0.07 | 0.78 | 0.05 | 0.36 | 0.17 | 0.27 | 0.20 | 0.54 | 0.11 | 0.92 | 0.02 |
| Alpha_C3 | 0.09 | 0.31 | 0.05 | 0.36 | 0.09 | 0.31 | 0.06 | 0.34 | 0.41 | 0.15 | 0.22 | 0.23 | 0.11 | 0.29 | 0.07 | 0.33 | 0.14 | 0.27 | 0.29 | 0.20 |
| Alpha_C4 | 0.34 | 0.18 | 0.19 | 0.24 | 0.29 | 0.20 | 0.26 | 0.21 | 0.93 | 0.02 | 0.51 | 0.12 | 0.36 | 0.17 | 0.27 | 0.20 | 0.44 | 0.14 | 0.55 | 0.11 |
| Alpha_Cz | 0.24 | 0.22 | 0.20 | 0.24 | 0.28 | 0.20 | 0.52 | 0.12 | 0.57 | 0.11 | 0.44 | 0.14 | 0.25 | 0.21 | 0.20 | 0.24 | 0.48 | 0.13 | 0.64 | 0.09 |
| Alpha_Pz | 0.36 | 0.17 | 0.32 | 0.18 | 0.69 | 0.07 | 0.12 | 0.28 | 0.55 | 0.11 | 0.78 | 0.05 | 0.26 | 0.21 | 0.14 | 0.27 | 0.28 | 0.20 | 0.34 | 0.18 |
| Alpha_Oz | 0.14 | 0.27 | 0.15 | 0.27 | 0.39 | 0.16 | 0.27 | 0.21 | 0.39 | 0.16 | 0.43 | 0.15 | 0.21 | 0.23 | 0.13 | 0.28 | 0.35 | 0.17 | 0.50 | 0.13 |
| Beta_Fz | 0.09 | -0.31 | 0.13 | -0.28 | 0.08 | -0.32 | 0.07 | -0.33 | 0.36 | -0.17 | 0.10 | -0.30 | 0.11 | -0.29 | 0.13 | -0.28 | 0.13 | -0.28 | 0.10 | -0.30 |
| Beta_C3 | 0.14 | -0.27 | 0.08 | -0.32 | 0.07 | -0.34 | 0.14 | -0.27 | 0.59 | -0.10 | 0.17 | -0.25 | 0.21 | -0.23 | 0.23 | -0.22 | 0.14 | -0.27 | 0.11 | -0.29 |
| Beta_C4 | 0.06 | -0.34 | 0.05 | -0.35 | 0.22 | -0.23 | 0.16 | -0.26 | 0.99 | 0.00 | 0.09 | -0.31 | 0.15 | -0.27 | 0.13 | -0.28 | 0.06 | -0.35 | 0.05 | -0.36 |
| Beta_Cz | 0.06 | -0.34 | 0.06 | -0.35 | 0.05 | -0.35 | 0.21 | -0.23 | 0.47 | -0.14 | 0.07 | -0.33 | 0.12 | -0.29 | 0.17 | -0.25 | 0.11 | -0.29 | 0.04 | -0.37 |
| Beta_Pz | 0.17 | -0.25 | 0.23 | -0.22 | 0.20 | -0.23 | 0.02 | -0.43 | 0.44 | -0.14 | 0.26 | -0.21 | 0.19 | -0.24 | 0.13 | -0.28 | 0.07 | -0.33 | 0.03 | -0.38 |
| Beta_Oz | 0.23 | -0.22 | 0.21 | -0.23 | 0.25 | -0.21 | 0.03 | -0.38 | 0.91 | -0.02 | 0.25 | -0.21 | 0.29 | -0.19 | 0.24 | -0.22 | 0.14 | -0.27 | 0.17 | -0.25 |
| Gamma_Fz | 0.63 | -0.09 | 0.54 | -0.12 | 0.37 | -0.17 | 0.13 | -0.28 | 0.71 | -0.07 | 0.63 | -0.09 | 0.38 | -0.16 | 0.36 | -0.17 | 0.32 | -0.19 | 0.56 | -0.11 |
| Gamma_C3 | 0.74 | -0.06 | 0.55 | -0.11 | 0.49 | -0.13 | 0.29 | -0.20 | 0.82 | -0.04 | 0.68 | -0.08 | 0.76 | -0.06 | 0.72 | -0.07 | 0.59 | -0.10 | 0.85 | -0.04 |
| Gamma_C4 | 0.81 | -0.04 | 0.68 | -0.08 | 0.55 | -0.11 | 0.44 | -0.14 | 0.92 | 0.02 | 0.79 | -0.05 | 0.89 | -0.03 | 0.80 | -0.05 | 0.73 | -0.06 | 0.97 | -0.01 |
| Gamma_Cz | 0.51 | -0.12 | 0.37 | -0.17 | 0.32 | -0.19 | 0.23 | -0.22 | 0.59 | -0.10 | 0.43 | -0.15 | 0.48 | -0.13 | 0.40 | -0.16 | 0.45 | -0.14 | 0.60 | -0.10 |
| Gamma_Pz | 0.25 | -0.22 | 0.25 | -0.21 | 0.24 | -0.22 | 0.03 | -0.38 | 0.55 | -0.11 | 0.44 | -0.14 | 0.27 | -0.20 | 0.16 | -0.26 | 0.22 | -0.23 | 0.26 | -0.21 |
| Gamma_Oz | 0.70 | -0.07 | 0.52 | -0.12 | 0.28 | -0.20 | 0.15 | -0.27 | 0.95 | 0.01 | 0.52 | -0.12 | 0.46 | -0.14 | 0.58 | -0.10 | 0.33 | -0.18 | 0.49 | -0.13 |

**Supplementary table S3.** **The correlation analysis between the resting-state EEG absolute power ratio and the mean FA values of impaired WM tracts in patients with NMOSD (P value and r value).** Pearson correlation analysis or Spearman correlation analysis were used according to the normality distributions of the data, with age as covariant. P<0.05 with false discovery rate corrected was considered significant, marked with **bold font**.

|  | ATR_L | | ATR_R | | CST_L | | CGH_R | | Fmaj | | Fmin | | IFO_L | | IFO_R | | ILF_L | | ILF_R | |
| --- | --- | --- | --- | --- | --- | --- | --- | --- | --- | --- | --- | --- | --- | --- | --- | --- | --- | --- | --- | --- |
|  | P | r | P | r | P | r | P | r | P | r | P | r | P | r | P | r | P | r | P | r |
| Alpha/Theta_Fz | 0.82 | 0.04 | 0.67 | 0.08 | 0.91 | -0.02 | 0.76 | 0.06 | 0.89 | -0.03 | 0.89 | 0.03 | 0.62 | 0.09 | 0.50 | 0.12 | 0.98 | 0.00 | 0.66 | -0.08 |
| Alpha/Theta_C3 | 0.13 | 0.28 | 0.07 | 0.33 | 0.37 | 0.17 | 0.54 | 0.12 | 0.33 | 0.18 | 0.21 | 0.23 | 0.19 | 0.24 | 0.08 | 0.32 | 0.38 | 0.16 | 0.55 | 0.11 |
| Alpha/Theta_C4 | 0.50 | 0.13 | 0.31 | 0.19 | 0.81 | 0.04 | 0.89 | 0.03 | 0.86 | 0.03 | 0.55 | 0.11 | 0.52 | 0.12 | 0.38 | 0.16 | 0.93 | 0.02 | 0.89 | -0.03 |
| Alpha/Theta_Cz | 0.47 | 0.13 | 0.36 | 0.17 | 0.93 | 0.02 | 0.90 | 0.02 | 0.75 | 0.06 | 0.59 | 0.10 | 0.41 | 0.15 | 0.34 | 0.18 | 0.68 | 0.08 | 1.00 | 0.00 |
| Alpha/Theta_Pz | 0.48 | 0.13 | 0.40 | 0.16 | 0.85 | 0.03 | 0.40 | 0.16 | 0.44 | 0.14 | 0.81 | 0.05 | 0.35 | 0.17 | 0.20 | 0.24 | 0.58 | 0.10 | 0.64 | 0.09 |
| Alpha/Theta_Oz | 0.16 | 0.26 | 0.15 | 0.26 | 0.45 | 0.14 | 0.65 | 0.08 | 0.39 | 0.16 | 0.31 | 0.19 | 0.18 | 0.25 | 0.13 | 0.28 | 0.48 | 0.13 | 0.71 | 0.07 |
| Alpha/Delta_Fz | 0.68 | 0.08 | 0.55 | 0.11 | 0.71 | 0.07 | 0.14 | 0.27 | 0.63 | 0.09 | 0.72 | 0.07 | 0.34 | 0.18 | 0.24 | 0.22 | 0.43 | 0.15 | 0.67 | 0.08 |
| Alpha/Delta_C3 | 0.20 | 0.24 | 0.10 | 0.30 | 0.24 | 0.22 | 0.05 | 0.36 | 0.46 | 0.14 | 0.23 | 0.22 | 0.18 | 0.25 | 0.09 | 0.31 | 0.16 | 0.26 | 0.26 | 0.21 |
| Alpha/Delta_C4 | 0.71 | 0.07 | 0.50 | 0.13 | 0.76 | 0.06 | 0.29 | 0.20 | 0.98 | -0.01 | 0.79 | 0.05 | 0.60 | 0.10 | 0.47 | 0.14 | 0.74 | 0.06 | 0.99 | 0.00 |
| Alpha/Delta_Cz | 0.30 | 0.19 | 0.22 | 0.23 | 0.39 | 0.16 | 0.18 | 0.25 | 0.40 | 0.16 | 0.28 | 0.20 | 0.16 | 0.26 | 0.12 | 0.28 | 0.23 | 0.22 | 0.44 | 0.14 |
| Alpha/Delta_Pz | 0.77 | 0.05 | 0.72 | 0.07 | 0.95 | -0.01 | 0.21 | 0.23 | 0.79 | 0.05 | 0.92 | -0.02 | 0.40 | 0.16 | 0.25 | 0.21 | 0.43 | 0.15 | 0.53 | 0.12 |
| Alpha/Delta_Oz | 0.58 | 0.10 | 0.62 | 0.09 | 0.94 | 0.01 | 0.50 | 0.13 | 0.67 | 0.08 | 0.86 | 0.03 | 0.37 | 0.17 | 0.32 | 0.19 | 0.53 | 0.12 | 0.79 | 0.05 |
| Delta/Theta_Fz | 0.70 | -0.07 | 0.71 | -0.07 | 0.41 | -0.15 | 0.04 | -0.38 | 0.43 | -0.15 | 0.68 | -0.08 | 0.37 | -0.17 | 0.32 | -0.18 | 0.16 | -0.26 | 0.15 | -0.26 |
| Delta/Theta_C3 | 0.99 | 0.00 | 0.83 | -0.04 | 0.47 | -0.13 | **0.01** | **-0.48** | 0.99 | 0.00 | 0.79 | -0.05 | 0.67 | -0.08 | 0.67 | -0.08 | 0.23 | -0.22 | 0.27 | -0.21 |
| Delta/Theta_C4 | 0.66 | 0.08 | 0.66 | 0.08 | 0.87 | -0.03 | 0.09 | -0.31 | 0.69 | 0.07 | 0.61 | 0.09 | 0.92 | 0.02 | 0.90 | 0.02 | 0.65 | -0.09 | 0.83 | -0.04 |
| Delta/Theta_Cz | 0.40 | -0.16 | 0.39 | -0.16 | 0.13 | -0.28 | **0.01** | **-0.43** | 0.48 | -0.13 | 0.24 | -0.22 | 0.17 | -0.25 | 0.16 | -0.26 | 0.10 | -0.30 | 0.13 | -0.28 |
| Delta/Theta_Pz | 0.33 | 0.18 | 0.48 | 0.13 | 0.70 | 0.07 | 0.10 | -0.30 | 0.72 | 0.07 | 0.43 | 0.15 | 0.86 | -0.03 | 0.96 | -0.01 | 0.67 | -0.08 | 0.98 | -0.01 |
| Delta/Theta_Oz | 0.29 | 0.19 | 0.36 | 0.17 | 0.63 | 0.09 | 0.57 | -0.11 | 0.56 | 0.11 | 0.38 | 0.16 | 0.63 | 0.09 | 0.65 | 0.08 | 0.69 | 0.07 | 0.84 | 0.04 |
| Theta/Beta_Fz | 0.15 | 0.27 | 0.13 | 0.28 | 0.08 | 0.32 | 0.05 | 0.36 | 0.22 | 0.23 | 0.14 | 0.27 | 0.24 | 0.22 | 0.21 | 0.23 | 0.20 | 0.24 | 0.05 | 0.36 |
| Theta/Beta_C3 | 0.32 | 0.18 | 0.24 | 0.22 | 0.17 | 0.25 | 0.08 | 0.32 | 0.56 | 0.11 | 0.34 | 0.18 | 0.37 | 0.17 | 0.37 | 0.17 | 0.23 | 0.22 | 0.15 | 0.26 |
| Theta/Beta_C4 | 0.19 | 0.24 | 0.23 | 0.22 | 0.33 | 0.18 | 0.15 | 0.27 | 0.88 | 0.03 | 0.30 | 0.19 | 0.32 | 0.18 | 0.33 | 0.18 | 0.10 | 0.31 | 0.07 | 0.33 |
| Theta/Beta_Cz | 0.07 | 0.33 | 0.05 | 0.35 | 0.05 | 0.36 | 0.14 | 0.27 | 0.33 | 0.18 | 0.05 | 0.35 | 0.19 | 0.24 | 0.19 | 0.24 | 0.11 | 0.29 | 0.03 | 0.39 |
| Theta/Beta_Pz | 0.33 | 0.18 | 0.22 | 0.23 | 0.13 | 0.28 | 0.04 | 0.38 | 0.52 | 0.12 | 0.25 | 0.21 | 0.36 | 0.17 | 0.36 | 0.17 | 0.19 | 0.24 | 0.08 | 0.32 |
| Theta/Beta_Oz | 0.63 | 0.09 | 0.34 | 0.18 | 0.30 | 0.19 | 0.04 | 0.36 | 0.81 | -0.05 | 0.56 | 0.11 | 0.91 | -0.02 | 0.74 | 0.06 | 0.94 | 0.01 | 0.54 | 0.11 |
| Alpha/Beta_Fz | 0.13 | 0.28 | 0.12 | 0.29 | 0.35 | 0.17 | 0.08 | 0.32 | 0.82 | 0.04 | 0.21 | 0.23 | 0.16 | 0.26 | 0.10 | 0.30 | 0.13 | 0.28 | 0.16 | 0.26 |
| Alpha/Beta_C3 | 0.03 | 0.38 | 0.02 | 0.42 | 0.11 | 0.29 | 0.07 | 0.33 | 0.61 | 0.09 | 0.09 | 0.31 | 0.09 | 0.31 | 0.05 | 0.36 | 0.06 | 0.34 | 0.06 | 0.34 |
| Alpha/Beta_C4 | 0.38 | 0.16 | 0.23 | 0.22 | 0.34 | 0.18 | 0.23 | 0.22 | 0.58 | -0.10 | 0.48 | 0.13 | 0.49 | 0.13 | 0.47 | 0.14 | 0.37 | 0.17 | 0.28 | 0.20 |
| Alpha/Beta_Cz | 0.10 | 0.30 | 0.05 | 0.36 | 0.09 | 0.31 | 0.28 | 0.20 | 0.54 | 0.12 | 0.09 | 0.31 | 0.18 | 0.25 | 0.17 | 0.25 | 0.26 | 0.21 | 0.11 | 0.29 |
| Alpha/Beta_Pz | 0.27 | 0.21 | 0.30 | 0.19 | 0.44 | 0.14 | 0.03 | 0.40 | 0.75 | 0.06 | 0.56 | 0.11 | 0.22 | 0.23 | 0.12 | 0.29 | 0.14 | 0.27 | 0.11 | 0.29 |
| Alpha/Beta_Oz | 0.18 | 0.24 | 0.18 | 0.25 | 0.34 | 0.18 | 0.07 | 0.33 | 0.97 | 0.01 | 0.36 | 0.17 | 0.27 | 0.20 | 0.19 | 0.24 | 0.24 | 0.22 | 0.32 | 0.19 |

**Supplementary table S4.** **The correlation analysis between the resting-state EEG COH strengths and the mean FA values of impaired WM tracts in patients with NMOSD (P value and r value).** Pearson correlation analysis or Spearman correlation analysis were used according to the normality distributions of the data, with age as covariant. P<0.05 with false discovery rate corrected was considered significant, marked with **bold font**.

|  | ATR_L | | ATR_R | | CST_L | | CGH_R | | Fmaj | | Fmin | | IFO_L | | IFO_R | | ILF_L | | ILF_R | |
| --- | --- | --- | --- | --- | --- | --- | --- | --- | --- | --- | --- | --- | --- | --- | --- | --- | --- | --- | --- | --- |
|  | P | r | P | r | P | r | P | r | P | r | P | r | P | r | P | r | P | r | P | r |
| Delta_Fz | 0.29 | 0.20 | 0.29 | 0.20 | 0.39 | 0.16 | 0.76 | 0.06 | 0.44 | 0.14 | 0.05 | 0.36 | 0.15 | 0.26 | 0.32 | 0.18 | 0.22 | 0.23 | 0.09 | 0.31 |
| Delta_C3 | 0.09 | -0.31 | 0.07 | -0.33 | 0.10 | -0.30 | 0.41 | -0.15 | 0.04 | -0.38 | 0.16 | -0.26 | 0.30 | -0.19 | 0.11 | -0.29 | 0.52 | -0.12 | 0.66 | -0.08 |
| Delta_C4 | 0.57 | -0.11 | 0.66 | -0.08 | 0.43 | -0.15 | 0.85 | -0.03 | 0.37 | -0.17 | 0.79 | -0.05 | 0.89 | 0.02 | 0.74 | -0.06 | 0.94 | 0.01 | 0.68 | 0.08 |
| Delta_Cz | 0.36 | 0.17 | 0.28 | 0.20 | 0.35 | 0.17 | 0.47 | -0.14 | 0.35 | 0.17 | 0.05 | 0.35 | 0.24 | 0.22 | 0.54 | 0.11 | 0.29 | 0.20 | 0.24 | 0.22 |
| Delta_Pz | 0.48 | 0.13 | 0.70 | 0.07 | 0.36 | 0.17 | 0.08 | 0.32 | 0.74 | 0.06 | 0.24 | 0.22 | 0.23 | 0.22 | 0.39 | 0.16 | 0.19 | 0.24 | 0.04 | 0.37 |
| Delta_Oz | 0.22 | 0.23 | 0.29 | 0.20 | 0.36 | 0.17 | 0.28 | 0.20 | 0.40 | 0.16 | 0.03 | 0.38 | 0.03 | 0.40 | 0.17 | 0.25 | 0.02 | 0.42 | 0.02 | 0.43 |
| Theta_Fz | 0.07 | 0.33 | 0.06 | 0.35 | 0.17 | 0.25 | 0.17 | 0.25 | 0.19 | 0.24 | **0.01** | **0.47** | 0.03 | 0.40 | 0.04 | 0.38 | 0.03 | 0.39 | **0.01** | **0.45** |
| Theta_C3 | 0.78 | 0.05 | 0.60 | 0.10 | 0.57 | 0.10 | 0.97 | -0.01 | 0.93 | 0.02 | 0.53 | 0.12 | 0.37 | 0.17 | 0.71 | 0.07 | 0.19 | 0.24 | 0.04 | 0.36 |
| Theta_C4 | 0.45 | 0.14 | 0.36 | 0.17 | 0.68 | 0.08 | 0.75 | 0.06 | 0.47 | 0.13 | 0.27 | 0.21 | 0.12 | 0.29 | 0.28 | 0.20 | 0.12 | 0.28 | 0.10 | 0.30 |
| Theta_Cz | 0.06 | 0.34 | 0.02 | 0.42 | 0.07 | 0.33 | 0.91 | 0.02 | 0.05 | 0.35 | **0.00** | **0.55** | 0.02 | 0.43 | 0.05 | 0.36 | 0.02 | 0.43 | **0.02** | **0.43** |
| Theta_Pz | 0.91 | 0.02 | 0.65 | -0.09 | 0.76 | 0.06 | 0.03 | 0.39 | 0.70 | 0.07 | 0.84 | 0.04 | 0.37 | 0.17 | 0.45 | 0.14 | 0.26 | 0.21 | 0.09 | 0.31 |
| Theta_Oz | 0.17 | 0.25 | 0.24 | 0.22 | 0.51 | 0.12 | 0.28 | 0.20 | 0.22 | 0.23 | 0.05 | 0.36 | 0.03 | 0.39 | 0.09 | 0.31 | 0.03 | 0.39 | 0.03 | 0.39 |
| Alpha_Fz | 0.40 | 0.16 | 0.27 | 0.21 | 0.72 | 0.07 | 0.22 | 0.23 | 0.44 | 0.14 | 0.24 | 0.22 | 0.16 | 0.26 | 0.14 | 0.27 | 0.38 | 0.16 | 0.36 | 0.17 |
| Alpha_C3 | 0.18 | 0.25 | 0.19 | 0.24 | 0.29 | 0.20 | 0.72 | 0.07 | 0.28 | 0.20 | 0.17 | 0.25 | 0.08 | 0.32 | 0.12 | 0.29 | 0.14 | 0.27 | 0.19 | 0.24 |
| Alpha_C4 | 0.60 | 0.10 | 0.44 | 0.14 | 0.98 | 0.00 | 0.89 | 0.03 | 0.56 | 0.11 | 0.43 | 0.15 | 0.22 | 0.23 | 0.35 | 0.17 | 0.37 | 0.17 | 0.50 | 0.13 |
| Alpha_Cz | 0.35 | 0.17 | 0.25 | 0.21 | 0.63 | 0.09 | 0.76 | -0.06 | 0.52 | 0.12 | 0.14 | 0.27 | 0.13 | 0.28 | 0.29 | 0.20 | 0.22 | 0.23 | 0.42 | 0.15 |
| Alpha_Pz | 0.98 | 0.00 | 0.72 | -0.07 | 0.92 | 0.02 | 0.05 | 0.35 | 0.49 | 0.13 | 1.00 | 0.00 | 0.27 | 0.20 | 0.28 | 0.20 | 0.37 | 0.17 | 0.25 | 0.21 |
| Alpha_Oz | 0.30 | 0.19 | 0.33 | 0.18 | 0.75 | 0.06 | 0.19 | 0.24 | 0.56 | 0.11 | 0.24 | 0.22 | 0.16 | 0.26 | 0.18 | 0.25 | 0.41 | 0.15 | 0.51 | 0.12 |
| Beta_Fz | 0.36 | 0.17 | 0.43 | 0.15 | 0.53 | 0.12 | 0.74 | 0.06 | 0.22 | 0.23 | 0.29 | 0.20 | 0.16 | 0.26 | 0.20 | 0.23 | 0.38 | 0.16 | 0.29 | 0.20 |
| Beta_C3 | 0.62 | 0.09 | 0.72 | 0.07 | 0.20 | 0.24 | 0.63 | -0.09 | 0.44 | 0.15 | 0.37 | 0.17 | 0.31 | 0.19 | 0.53 | 0.12 | 0.46 | 0.14 | 0.42 | 0.15 |
| Beta_C4 | 0.92 | 0.02 | 0.88 | -0.03 | 0.75 | 0.06 | 0.19 | -0.24 | 0.19 | 0.24 | 0.55 | 0.11 | 0.09 | 0.31 | 0.40 | 0.16 | 0.23 | 0.22 | 0.23 | 0.22 |
| Beta_Cz | 0.24 | 0.22 | 0.14 | 0.27 | 0.16 | 0.26 | 0.09 | -0.31 | 0.03 | 0.38 | 0.06 | 0.34 | 0.08 | 0.32 | 0.22 | 0.23 | 0.45 | 0.14 | 0.50 | 0.13 |
| Beta_Pz | 0.80 | -0.05 | 0.26 | -0.21 | 0.61 | -0.10 | 0.42 | 0.15 | 0.46 | -0.14 | 0.50 | -0.13 | 0.72 | -0.07 | 0.60 | -0.10 | 0.95 | -0.01 | 0.73 | -0.06 |
| Beta_Oz | 0.49 | 0.13 | 0.63 | 0.09 | 0.87 | 0.03 | 0.94 | -0.02 | 0.61 | 0.10 | 0.54 | 0.11 | 0.33 | 0.18 | 0.51 | 0.12 | 0.69 | 0.07 | 0.96 | -0.01 |
| Gamma_Fz | 0.78 | 0.05 | 0.85 | 0.04 | 0.61 | -0.10 | 0.93 | -0.02 | 0.74 | 0.06 | 0.62 | 0.09 | 0.65 | 0.08 | 0.51 | 0.12 | 0.99 | 0.00 | 0.81 | 0.04 |
| Gamma_C3 | 0.35 | 0.17 | 0.32 | 0.18 | 0.59 | 0.10 | 0.75 | -0.06 | 0.57 | -0.11 | 0.34 | 0.18 | 0.57 | 0.11 | 0.56 | 0.11 | 0.63 | 0.09 | 0.60 | 0.10 |
| Gamma_C4 | 0.30 | 0.19 | 0.61 | 0.10 | 0.52 | 0.12 | 0.29 | -0.20 | 0.85 | 0.04 | 0.31 | 0.19 | 0.53 | 0.12 | 0.62 | 0.09 | 0.92 | -0.02 | 0.96 | 0.01 |
| Gamma_Cz | **0.00** | **0.55** | **0.01** | **0.48** | **0.00** | **0.49** | 0.24 | -0.22 | **0.01** | **0.48** | **0.00** | **0.57** | **0.01** | **0.47** | 0.04 | 0.38 | 0.09 | 0.31 | 0.10 | 0.30 |
| Gamma_Pz | 0.36 | -0.17 | 0.33 | -0.18 | 0.71 | -0.07 | 0.43 | 0.15 | 0.19 | -0.24 | 0.55 | -0.11 | 0.63 | -0.09 | 0.52 | -0.12 | 0.82 | -0.04 | 0.99 | 0.00 |
| Gamma_Oz | 0.13 | 0.28 | 0.43 | 0.15 | 0.63 | 0.09 | 0.19 | 0.24 | 0.80 | 0.05 | 0.28 | 0.20 | 0.32 | 0.18 | 0.32 | 0.18 | 0.45 | 0.14 | 0.54 | 0.11 |

**Supplementary table S5.** **The correlation analysis between the resting-state EEG iCOH strengths and the mean FA values of impaired WM tracts in patients with NMOSD (P value and r value).** Pearson correlation analysis or Spearman correlation analysis were used according to the normality distributions of the data, with age as covariant. P<0.05 with false discovery rate corrected was considered significant, marked with **bold font**.

|  | ATR_L | | ATR_R | | CST_L | | CGH_R | | Fmaj | | Fmin | | IFO_L | | IFO_R | | ILF_L | | ILF_R | |
| --- | --- | --- | --- | --- | --- | --- | --- | --- | --- | --- | --- | --- | --- | --- | --- | --- | --- | --- | --- | --- |
|  | P | r | P | r | P | r | P | r | P | r | P | r | P | r | P | r | P | r | P | r |
| Delta_Fz | 0.29 | -0.20 | 0.78 | -0.19 | 0.99 | -0.05 | 0.66 | 0.00 | 0.61 | -0.08 | 0.98 | -0.09 | 0.32 | 0.01 | 0.82 | -0.18 | 0.95 | 0.04 | 0.32 | 0.01 |
| Delta_C3 | 0.14 | -0.22 | 0.69 | -0.27 | 0.69 | -0.08 | 0.43 | 0.08 | 0.13 | -0.15 | 0.42 | -0.28 | 0.27 | -0.15 | 0.59 | -0.21 | 0.25 | -0.10 | 0.41 | -0.21 |
| Delta_C4 | 0.44 | -0.08 | 0.97 | -0.14 | 0.30 | -0.01 | 0.74 | 0.19 | 0.87 | -0.06 | 0.91 | -0.03 | 0.75 | 0.02 | 0.55 | -0.06 | 0.96 | 0.11 | 0.68 | -0.01 |
| Delta_Cz | 0.67 | -0.11 | 0.99 | -0.08 | 0.58 | 0.00 | 0.59 | -0.10 | 0.92 | -0.10 | 0.74 | -0.02 | 0.30 | -0.06 | 0.87 | -0.19 | 0.58 | -0.03 | 0.15 | -0.10 |
| Delta_Pz | 0.75 | 0.12 | 0.19 | 0.06 | 0.41 | 0.24 | 0.22 | 0.15 | 0.43 | 0.23 | 0.09 | 0.15 | 0.36 | 0.31 | 0.05 | 0.17 | 0.07 | 0.36 | 0.04 | 0.33 |
| Delta_Oz | 0.58 | 0.11 | 0.43 | 0.10 | 0.27 | 0.15 | 0.45 | 0.20 | 0.26 | 0.14 | 0.12 | 0.21 | 0.33 | 0.29 | 0.12 | 0.18 | 0.15 | 0.29 | 0.05 | 0.26 |
| Theta_Fz | 0.20 | -0.17 | 0.71 | -0.24 | 0.12 | -0.07 | 0.56 | 0.29 | 0.36 | -0.11 | 0.77 | -0.17 | 0.70 | -0.06 | 0.62 | -0.07 | 0.52 | 0.09 | 0.18 | 0.12 |
| Theta_C3 | 0.60 | -0.05 | 0.94 | -0.10 | 0.05 | -0.01 | 0.66 | 0.35 | 0.63 | -0.08 | 0.61 | -0.09 | 0.79 | 0.09 | 0.22 | 0.05 | 0.51 | 0.23 | 0.89 | 0.12 |
| Theta_C4 | 0.62 | -0.06 | 0.52 | -0.09 | 0.10 | -0.12 | 0.35 | 0.30 | 0.39 | -0.17 | 0.82 | -0.16 | 0.78 | 0.04 | 0.34 | 0.05 | 0.64 | 0.18 | 0.61 | 0.09 |
| Theta_Cz | 0.49 | -0.03 | 0.82 | -0.13 | 0.10 | 0.04 | 0.80 | 0.30 | 0.88 | 0.05 | 0.48 | -0.03 | 0.72 | 0.13 | 0.13 | 0.07 | 0.14 | 0.28 | 0.25 | 0.27 |
| Theta_Pz | 0.35 | -0.08 | 0.88 | -0.17 | 0.02 | -0.03 | 0.81 | 0.42 | 0.85 | 0.05 | 0.54 | -0.04 | 0.68 | 0.12 | 0.29 | 0.08 | 0.22 | 0.19 | 0.59 | 0.22 |
| Theta_Oz | 0.59 | 0.17 | 0.44 | 0.10 | 0.34 | 0.14 | 0.48 | 0.18 | 0.43 | 0.13 | 0.13 | 0.15 | 0.21 | 0.28 | 0.28 | 0.23 | 0.47 | 0.20 | 0.34 | 0.13 |
| Alpha_Fz | 0.48 | -0.13 | 0.41 | -0.13 | 0.96 | -0.15 | 0.82 | 0.01 | 0.39 | -0.04 | 0.78 | -0.16 | 0.82 | 0.05 | 0.80 | 0.04 | 0.98 | -0.05 | 0.35 | 0.00 |
| Alpha_C3 | 0.89 | 0.08 | 0.74 | -0.03 | 0.86 | 0.06 | 0.42 | 0.03 | 0.80 | 0.15 | 0.36 | 0.05 | 0.52 | 0.17 | 0.92 | 0.12 | 0.81 | 0.02 | 0.21 | 0.05 |
| Alpha_C4 | 0.17 | 0.17 | 0.46 | 0.25 | 0.35 | 0.14 | 0.63 | 0.17 | 0.23 | 0.09 | 0.22 | 0.22 | 0.24 | 0.23 | 0.50 | 0.22 | 0.22 | 0.12 | 0.26 | 0.23 |
| Alpha_Cz | 0.45 | -0.15 | 0.54 | -0.14 | 0.88 | -0.11 | 0.65 | -0.03 | 0.63 | -0.09 | 0.95 | -0.09 | 0.61 | 0.01 | 0.72 | -0.10 | 0.99 | -0.07 | 0.99 | 0.00 |
| Alpha_Pz | 0.10 | -0.30 | 0.07 | -0.30 | 0.73 | -0.33 | 0.23 | 0.06 | 0.09 | -0.22 | 0.52 | -0.31 | 0.47 | -0.12 | 0.45 | -0.13 | 0.81 | -0.14 | 0.54 | -0.04 |
| Alpha_Oz | 0.82 | 0.10 | 0.99 | 0.04 | 0.77 | 0.00 | 0.58 | -0.05 | 0.87 | 0.10 | 0.29 | 0.03 | 0.34 | 0.20 | 0.85 | 0.18 | 0.81 | 0.04 | 0.20 | 0.04 |
| Beta_Fz | 0.48 | -0.05 | 0.41 | -0.09 | 0.96 | 0.04 | 0.82 | -0.21 | 0.39 | 0.08 | 0.78 | 0.06 | 0.82 | 0.03 | 0.80 | -0.01 | 0.98 | -0.14 | 0.35 | -0.09 |
| Beta_C3 | 0.89 | -0.04 | 0.74 | -0.07 | 0.86 | 0.05 | 0.42 | -0.16 | 0.80 | 0.17 | 0.36 | 0.06 | 0.52 | 0.07 | 0.92 | -0.01 | 0.81 | -0.13 | 0.21 | -0.18 |
| Beta_C4 | 0.17 | -0.07 | 0.46 | -0.07 | 0.35 | 0.00 | 0.63 | -0.13 | 0.23 | 0.08 | 0.22 | 0.07 | 0.24 | 0.03 | 0.50 | -0.02 | 0.22 | -0.11 | 0.26 | -0.10 |
| Beta_Cz | 0.45 | -0.04 | 0.54 | -0.08 | 0.88 | 0.04 | 0.65 | -0.19 | 0.63 | 0.15 | 0.95 | 0.00 | 0.61 | 0.05 | 0.72 | 0.00 | 0.99 | -0.10 | 0.99 | -0.15 |
| Beta_Pz | 0.10 | -0.03 | 0.07 | -0.11 | 0.73 | 0.04 | 0.23 | -0.19 | 0.09 | 0.17 | 0.52 | 0.05 | 0.47 | 0.12 | 0.45 | 0.01 | 0.81 | -0.07 | 0.54 | -0.09 |
| Beta_Oz | 0.82 | 0.22 | 0.99 | 0.16 | 0.77 | 0.26 | 0.58 | -0.21 | 0.87 | 0.37 | 0.29 | 0.32 | 0.34 | 0.36 | 0.85 | 0.23 | 0.81 | 0.19 | 0.20 | 0.15 |
| Gamma_Fz | 0.63 | -0.07 | 0.20 | -0.09 | 0.31 | -0.24 | 0.11 | -0.19 | 0.47 | -0.29 | 0.40 | -0.14 | 0.63 | -0.16 | 0.38 | -0.09 | 0.44 | -0.16 | 0.92 | -0.14 |
| Gamma_C3 | 0.81 | -0.01 | 0.34 | -0.05 | 0.74 | -0.18 | 0.27 | 0.06 | 0.58 | -0.20 | 0.80 | -0.10 | 0.85 | 0.05 | 0.74 | 0.03 | 0.95 | 0.06 | 0.65 | 0.01 |
| Gamma_C4 | 0.80 | -0.03 | 0.17 | -0.05 | 0.36 | -0.25 | 0.23 | -0.17 | 0.76 | -0.22 | 1.00 | -0.06 | 0.97 | 0.00 | 0.99 | 0.01 | 0.83 | 0.00 | 0.41 | -0.04 |
| Gamma_Cz | 0.91 | 0.04 | 0.33 | 0.02 | 0.45 | -0.18 | 0.45 | -0.14 | 0.68 | -0.14 | 0.96 | -0.08 | 0.88 | -0.01 | 0.98 | 0.03 | 0.68 | 0.00 | 0.87 | -0.08 |
| Gamma_Pz | 0.42 | -0.10 | 0.06 | -0.15 | 0.13 | -0.35 | 0.23 | -0.28 | 0.31 | -0.22 | 0.53 | -0.19 | 0.52 | -0.12 | 0.37 | -0.12 | 0.21 | -0.17 | 0.23 | -0.23 |
| Gamma_Oz | 0.55 | 0.17 | 0.47 | 0.11 | 0.45 | -0.13 | 0.49 | 0.14 | 0.77 | -0.13 | 0.61 | 0.05 | 0.27 | 0.10 | 0.51 | 0.20 | 0.43 | 0.12 | 0.35 | 0.15 |

**Supplementary table S6.** **The correlation analysis between the resting-state EEG PLV strengths and the mean FA values of impaired WM tracts in patients with NMOSD (P value and r value).** Pearson correlation analysis or Spearman correlation analysis were used according to the normality distributions of the data, with age as covariant. P<0.05 with false discovery rate corrected was considered significant, marked with **bold font**.

|  | ATR_L | | ATR_R | | CST_L | | CGH_R | | Fmaj | | Fmin | | IFO_L | | IFO_R | | ILF_L | | ILF_R | |
| --- | --- | --- | --- | --- | --- | --- | --- | --- | --- | --- | --- | --- | --- | --- | --- | --- | --- | --- | --- | --- |
|  | P | r | P | r | P | r | P | r | P | r | P | r | P | r | P | r | P | r | P | r |
| Delta_Fz | 0.36 | 0.17 | 0.61 | 0.09 | 0.34 | 0.18 | 0.46 | 0.14 | 0.52 | 0.12 | 0.10 | 0.30 | 0.21 | 0.23 | 0.42 | 0.15 | 0.31 | 0.19 | 0.33 | 0.18 |
| Delta_C3 | 0.40 | -0.16 | 0.32 | -0.19 | 0.30 | -0.19 | 0.22 | -0.23 | 0.56 | -0.11 | 0.53 | -0.12 | 0.84 | -0.04 | 0.41 | -0.15 | 0.97 | -0.01 | 0.98 | 0.00 |
| Delta_C4 | 0.51 | -0.12 | 0.41 | -0.15 | 0.48 | -0.13 | 0.69 | -0.07 | 0.69 | -0.07 | 0.72 | -0.07 | 0.75 | 0.06 | 0.74 | -0.06 | 0.65 | 0.09 | 0.27 | 0.20 |
| Delta_Cz | 0.30 | 0.19 | 0.34 | 0.18 | 0.44 | 0.14 | 0.14 | -0.27 | 0.20 | 0.24 | 0.10 | 0.30 | 0.18 | 0.25 | 0.41 | 0.15 | 0.32 | 0.19 | 0.41 | 0.15 |
| Delta_Pz | 0.61 | 0.09 | 0.91 | 0.02 | 0.29 | 0.19 | 0.05 | 0.36 | 0.93 | -0.02 | 0.40 | 0.16 | 0.32 | 0.19 | 0.48 | 0.13 | 0.29 | 0.20 | 0.07 | 0.33 |
| Delta_Oz | 0.18 | 0.25 | 0.27 | 0.21 | 0.26 | 0.21 | 0.19 | 0.24 | 0.47 | 0.13 | 0.05 | 0.36 | 0.04 | 0.37 | 0.17 | 0.26 | 0.06 | 0.34 | 0.07 | 0.33 |
| Theta_Fz | 0.06 | 0.34 | 0.06 | 0.35 | 0.15 | 0.27 | 0.07 | 0.33 | 0.13 | 0.28 | **0.01** | **0.47** | **0.03** | **0.40** | 0.03 | 0.40 | **0.02** | **0.40** | **0.02** | **0.42** |
| Theta_C3 | 0.27 | 0.21 | 0.14 | 0.27 | 0.17 | 0.25 | 0.81 | 0.05 | 0.42 | 0.15 | 0.16 | 0.26 | 0.13 | 0.28 | 0.31 | 0.19 | 0.11 | 0.29 | 0.04 | 0.38 |
| Theta_C4 | 0.12 | 0.28 | 0.16 | 0.26 | 0.22 | 0.23 | 0.44 | 0.14 | 0.19 | 0.24 | 0.07 | 0.33 | **0.01** | **0.44** | 0.08 | 0.32 | **0.02** | **0.41** | **0.02** | **0.42** |
| Theta_Cz | 0.04 | 0.37 | 0.01 | 0.44 | 0.06 | 0.34 | 0.91 | 0.02 | **0.01** | **0.48** | **0.00** | **0.56** | **0.02** | **0.43** | 0.04 | 0.38 | **0.02** | **0.42** | **0.02** | **0.41** |
| Theta_Pz | 0.87 | -0.03 | 0.46 | -0.14 | 0.84 | 0.04 | 0.03 | 0.39 | 0.97 | -0.01 | 0.95 | -0.01 | 0.54 | 0.12 | 0.62 | 0.09 | 0.37 | 0.17 | 0.23 | 0.22 |
| Theta_Oz | 0.10 | 0.30 | 0.17 | 0.25 | 0.34 | 0.18 | 0.12 | 0.29 | 0.15 | 0.27 | 0.03 | 0.39 | **0.02** | **0.41** | 0.05 | 0.35 | **0.02** | **0.41** | 0.04 | 0.37 |
| Alpha_Fz | 0.29 | 0.20 | 0.29 | 0.20 | 0.52 | 0.12 | 0.29 | 0.20 | 0.46 | 0.14 | 0.26 | 0.21 | 0.17 | 0.25 | 0.19 | 0.24 | 0.38 | 0.16 | 0.67 | 0.08 |
| Alpha_C3 | 0.08 | 0.32 | 0.09 | 0.31 | 0.20 | 0.24 | 0.85 | 0.04 | 0.24 | 0.22 | 0.13 | 0.28 | 0.08 | 0.32 | 0.11 | 0.30 | 0.17 | 0.25 | 0.37 | 0.17 |
| Alpha_C4 | 0.32 | 0.19 | 0.24 | 0.22 | 0.61 | 0.10 | 0.52 | 0.12 | 0.25 | 0.21 | 0.25 | 0.21 | 0.16 | 0.26 | 0.22 | 0.23 | 0.30 | 0.19 | 0.54 | 0.12 |
| Alpha_Cz | 0.20 | 0.24 | 0.19 | 0.24 | 0.47 | 0.14 | 0.74 | -0.06 | 0.25 | 0.21 | 0.11 | 0.29 | 0.11 | 0.29 | 0.26 | 0.21 | 0.21 | 0.23 | 0.63 | 0.09 |
| Alpha_Pz | 0.71 | -0.07 | 0.61 | -0.09 | 0.99 | 0.00 | 0.11 | 0.30 | 0.80 | 0.05 | 0.82 | -0.04 | 0.56 | 0.11 | 0.58 | 0.10 | 0.69 | 0.08 | 0.64 | 0.09 |
| Alpha_Oz | 0.25 | 0.21 | 0.29 | 0.20 | 0.57 | 0.11 | 0.18 | 0.25 | 0.59 | 0.10 | 0.31 | 0.19 | 0.20 | 0.24 | 0.19 | 0.24 | 0.50 | 0.12 | 0.80 | 0.05 |
| Beta_Fz | 0.20 | 0.24 | 0.26 | 0.21 | 0.15 | 0.26 | 0.71 | 0.07 | 0.12 | 0.28 | 0.15 | 0.27 | 0.13 | 0.28 | 0.18 | 0.24 | 0.38 | 0.16 | 0.41 | 0.15 |
| Beta_C3 | 0.29 | 0.20 | 0.38 | 0.16 | 0.06 | 0.34 | 0.83 | -0.04 | 0.09 | 0.31 | 0.24 | 0.22 | 0.20 | 0.24 | 0.29 | 0.20 | 0.39 | 0.16 | 0.46 | 0.14 |
| Beta_C4 | 0.85 | -0.03 | 0.69 | -0.07 | 0.80 | 0.05 | 0.14 | -0.27 | 0.22 | 0.23 | 0.67 | 0.08 | 0.23 | 0.22 | 0.72 | 0.07 | 0.48 | 0.13 | 0.60 | 0.10 |
| Beta_Cz | 0.32 | 0.18 | 0.23 | 0.22 | 0.15 | 0.26 | 0.09 | -0.31 | 0.03 | 0.38 | 0.11 | 0.29 | 0.11 | 0.29 | 0.25 | 0.21 | 0.65 | 0.09 | 0.77 | 0.06 |
| Beta_Pz | 0.93 | -0.02 | 0.37 | -0.17 | 0.93 | -0.02 | 0.31 | 0.19 | 0.69 | -0.08 | 0.68 | -0.08 | 0.84 | -0.04 | 0.81 | -0.05 | 0.80 | -0.05 | 0.40 | -0.16 |
| Beta_Oz | 0.46 | 0.14 | 0.53 | 0.12 | 0.58 | 0.10 | 0.75 | -0.06 | 0.55 | 0.11 | 0.45 | 0.14 | 0.40 | 0.16 | 0.56 | 0.11 | 0.96 | 0.01 | 0.68 | -0.08 |
| Gamma_Fz | 0.59 | 0.10 | 0.77 | 0.06 | 0.81 | -0.04 | 0.89 | -0.03 | 0.58 | 0.10 | 0.56 | 0.11 | 0.59 | 0.10 | 0.45 | 0.14 | 0.85 | 0.03 | 0.69 | 0.07 |
| Gamma_C3 | 0.15 | 0.26 | 0.12 | 0.28 | 0.38 | 0.16 | 0.99 | 0.00 | 0.88 | -0.03 | 0.18 | 0.25 | 0.43 | 0.15 | 0.32 | 0.18 | 0.55 | 0.11 | 0.47 | 0.14 |
| Gamma_C4 | 0.34 | 0.18 | 0.55 | 0.11 | 0.64 | 0.09 | 0.33 | -0.18 | 0.82 | -0.04 | 0.29 | 0.20 | 0.92 | -0.02 | 0.85 | 0.03 | 0.48 | -0.13 | 0.83 | -0.04 |
| Gamma_Cz | **0.00** | **0.59** | **0.00** | **0.52** | **0.00** | **0.53** | 0.40 | -0.16 | **0.01** | **0.49** | **0.00** | **0.61** | 0.01 | 0.44 | 0.03 | 0.40 | 0.15 | 0.26 | 0.16 | 0.26 |
| Gamma_Pz | 0.37 | -0.17 | 0.19 | -0.24 | 0.53 | -0.12 | 0.26 | 0.21 | 0.18 | -0.25 | 0.34 | -0.18 | 0.53 | -0.12 | 0.32 | -0.18 | 0.95 | 0.01 | 0.93 | 0.02 |
| Gamma_Oz | 0.05 | 0.35 | 0.22 | 0.23 | 0.31 | 0.19 | 0.14 | 0.27 | 0.45 | 0.14 | 0.15 | 0.26 | 0.18 | 0.25 | 0.16 | 0.26 | 0.26 | 0.21 | 0.34 | 0.18 |

**Supplementary table S7.** **The correlation analysis between the resting-state EEG PLI strengths and the mean FA values of impaired WM tracts in patients with NMOSD (P value and r value).** Pearson correlation analysis or Spearman correlation analysis were used according to the normality distributions of the data, with age as covariant. P<0.05 with false discovery rate corrected was considered significant, marked with **bold font**.

|  | ATR_L | | ATR_R | | CST_L | | CGH_R | | Fmaj | | Fmin | | IFO_L | | IFO_R | | ILF_L | | ILF_R | |
| --- | --- | --- | --- | --- | --- | --- | --- | --- | --- | --- | --- | --- | --- | --- | --- | --- | --- | --- | --- | --- |
|  | P | r | P | r | P | r | P | r | P | r | P | r | P | r | P | r | P | r | P | r |
| Delta_Fz | 0.73 | -0.06 | 0.41 | -0.15 | 0.96 | -0.01 | 0.93 | 0.02 | 0.75 | 0.06 | 0.79 | -0.05 | 0.68 | 0.08 | 0.79 | -0.05 | 0.51 | 0.12 | 0.61 | 0.09 |
| Delta_C3 | 0.79 | -0.05 | 0.36 | -0.17 | 0.65 | 0.08 | 0.62 | 0.09 | 0.97 | 0.01 | 0.53 | -0.12 | 0.94 | 0.01 | 0.73 | -0.07 | 0.99 | 0.00 | 0.71 | -0.07 |
| Delta_C4 | 0.73 | 0.07 | 0.89 | 0.03 | 0.27 | 0.21 | 0.21 | 0.23 | 0.47 | 0.14 | 0.42 | 0.15 | 0.25 | 0.21 | 0.67 | 0.08 | 0.30 | 0.19 | 0.63 | 0.09 |
| Delta_Cz | 0.43 | 0.15 | 0.31 | 0.19 | 0.18 | 0.25 | 0.68 | 0.08 | 0.59 | 0.10 | 0.38 | 0.16 | 0.44 | 0.15 | 0.63 | 0.09 | 0.37 | 0.17 | 0.52 | 0.12 |
| Delta_Pz | 0.33 | 0.18 | 0.65 | 0.09 | 0.19 | 0.24 | 0.38 | 0.16 | 0.22 | 0.23 | 0.36 | 0.17 | 0.08 | 0.32 | 0.36 | 0.17 | 0.02 | 0.41 | 0.06 | 0.34 |
| Delta_Oz | 0.33 | 0.18 | 0.82 | 0.04 | 0.32 | 0.19 | 0.17 | 0.25 | 0.14 | 0.27 | 0.33 | 0.18 | 0.16 | 0.26 | 0.19 | 0.24 | 0.18 | 0.25 | 0.34 | 0.18 |
| Theta_Fz | 0.35 | -0.17 | 0.38 | -0.16 | 0.51 | -0.12 | 0.17 | 0.25 | 0.39 | -0.16 | 0.38 | -0.16 | 0.48 | -0.13 | 0.78 | -0.05 | 0.58 | -0.10 | 0.46 | -0.14 |
| Theta_C3 | 0.64 | -0.09 | 0.53 | -0.12 | 0.92 | -0.02 | 0.03 | 0.38 | 0.56 | -0.11 | 0.65 | -0.08 | 0.77 | 0.05 | 0.97 | -0.01 | 0.51 | 0.12 | 0.97 | -0.01 |
| Theta_C4 | 0.52 | 0.12 | 0.58 | 0.10 | 0.70 | 0.07 | 0.09 | 0.31 | 0.89 | 0.03 | 0.91 | 0.02 | 0.20 | 0.24 | 0.26 | 0.21 | 0.15 | 0.27 | 0.40 | 0.16 |
| Theta_Cz | 0.27 | 0.20 | 0.46 | 0.14 | 0.37 | 0.17 | 0.07 | 0.33 | 0.29 | 0.20 | 0.36 | 0.17 | 0.22 | 0.23 | 0.10 | 0.30 | 0.19 | 0.24 | 0.42 | 0.15 |
| Theta_Pz | 0.71 | -0.07 | 0.51 | -0.12 | 0.98 | 0.00 | 0.08 | 0.32 | 0.94 | 0.01 | 0.95 | -0.01 | 0.51 | 0.12 | 0.64 | 0.09 | 0.42 | 0.15 | 0.59 | 0.10 |
| Theta_Oz | 0.25 | 0.21 | 0.32 | 0.19 | 0.33 | 0.18 | 0.37 | 0.17 | 0.54 | 0.11 | 0.40 | 0.16 | 0.42 | 0.15 | 0.25 | 0.21 | 0.79 | 0.05 | 0.94 | 0.01 |
| Alpha_Fz | 0.66 | -0.08 | 0.39 | -0.16 | 0.55 | -0.11 | 0.61 | 0.10 | 0.80 | 0.05 | 0.39 | -0.16 | 0.66 | 0.08 | 0.91 | 0.02 | 0.99 | 0.00 | 0.76 | -0.06 |
| Alpha_C3 | 0.48 | 0.13 | 0.86 | 0.03 | 0.31 | 0.19 | 0.53 | 0.12 | 0.33 | 0.18 | 0.72 | 0.07 | 0.32 | 0.19 | 0.40 | 0.16 | 0.97 | 0.01 | 0.97 | 0.01 |
| Alpha_C4 | 0.17 | 0.25 | 0.10 | 0.30 | 0.23 | 0.22 | 0.21 | 0.23 | 0.33 | 0.18 | 0.11 | 0.30 | 0.10 | 0.30 | 0.11 | 0.29 | 0.35 | 0.17 | 0.24 | 0.22 |
| Alpha_Cz | 0.91 | -0.02 | 0.88 | -0.03 | 0.88 | 0.03 | 0.94 | -0.01 | 0.79 | 0.05 | 0.78 | 0.05 | 0.42 | 0.15 | 0.89 | 0.03 | 0.89 | 0.03 | 0.89 | 0.03 |
| Alpha_Pz | 0.18 | -0.24 | 0.14 | -0.27 | 0.20 | -0.24 | 0.39 | 0.16 | 0.28 | -0.20 | 0.21 | -0.23 | 0.94 | -0.01 | 0.68 | -0.08 | 0.89 | -0.02 | 0.63 | -0.09 |
| Alpha_Oz | 0.46 | 0.14 | 0.67 | 0.08 | 0.89 | 0.02 | 0.92 | 0.02 | 0.56 | 0.11 | 0.89 | 0.03 | 0.33 | 0.18 | 0.46 | 0.14 | 0.83 | 0.04 | 0.84 | -0.04 |
| Beta_Fz | 0.75 | -0.06 | 0.66 | -0.08 | 0.62 | 0.09 | 0.36 | -0.17 | 0.36 | 0.17 | 0.90 | 0.02 | 0.72 | 0.07 | 0.90 | 0.02 | 0.75 | -0.06 | 0.49 | -0.13 |
| Beta_C3 | 0.52 | -0.12 | 0.44 | -0.14 | 0.95 | -0.01 | 0.35 | -0.17 | 0.36 | 0.17 | 0.92 | -0.02 | 0.96 | 0.01 | 0.74 | -0.06 | 0.36 | -0.17 | 0.19 | -0.24 |
| Beta_C4 | 0.41 | -0.15 | 0.37 | -0.17 | 0.96 | -0.01 | 0.48 | -0.13 | 0.85 | 0.03 | 0.96 | 0.01 | 0.85 | -0.04 | 0.50 | -0.13 | 0.58 | -0.10 | 0.50 | -0.12 |
| Beta_Cz | 0.63 | -0.09 | 0.52 | -0.12 | 0.63 | 0.09 | 0.55 | -0.11 | 0.44 | 0.14 | 0.93 | -0.02 | 0.71 | 0.07 | 0.88 | -0.03 | 0.76 | -0.06 | 0.35 | -0.17 |
| Beta_Pz | 0.37 | -0.17 | 0.20 | -0.23 | 0.85 | -0.04 | 0.19 | -0.24 | 0.52 | 0.12 | 0.40 | -0.16 | 0.73 | 0.06 | 0.78 | -0.05 | 0.74 | -0.06 | 0.25 | -0.21 |
| Beta_Oz | 0.87 | 0.03 | 0.88 | -0.03 | 0.47 | 0.14 | 0.13 | -0.28 | 0.12 | 0.28 | 0.51 | 0.12 | 0.47 | 0.14 | 0.90 | 0.02 | 0.92 | -0.02 | 0.44 | -0.14 |
| Gamma_Fz | 0.76 | 0.06 | 0.79 | 0.05 | 0.88 | -0.03 | 0.10 | -0.30 | 0.47 | -0.14 | 0.72 | 0.07 | 0.59 | -0.10 | 0.64 | -0.09 | 0.35 | -0.17 | 0.39 | -0.16 |
| Gamma_C3 | 0.83 | -0.04 | 0.86 | -0.03 | 0.23 | -0.22 | 0.89 | 0.03 | 0.29 | -0.20 | 0.56 | -0.11 | 0.98 | 0.00 | 0.86 | 0.03 | 0.71 | 0.07 | 0.63 | 0.09 |
| Gamma_C4 | 0.88 | 0.03 | 0.67 | -0.08 | 0.35 | -0.17 | 0.03 | -0.39 | 0.25 | -0.21 | 0.60 | -0.10 | 0.63 | -0.09 | 0.73 | -0.06 | 0.72 | -0.07 | 0.67 | -0.08 |
| Gamma_Cz | 0.75 | -0.06 | 0.93 | -0.02 | 0.27 | -0.20 | 0.24 | -0.22 | 0.18 | -0.25 | 0.51 | -0.12 | 0.30 | -0.19 | 0.53 | -0.12 | 0.36 | -0.17 | 0.26 | -0.21 |
| Gamma_Pz | 0.16 | -0.26 | 0.17 | -0.25 | 0.02 | -0.41 | 0.04 | -0.38 | 0.12 | -0.28 | 0.17 | -0.25 | 0.35 | -0.17 | 0.18 | -0.25 | 0.19 | -0.24 | 0.12 | -0.29 |
| Gamma_Oz | 0.46 | 0.14 | 0.74 | 0.06 | 0.72 | -0.07 | 0.52 | -0.12 | 0.75 | -0.06 | 0.55 | 0.11 | 0.77 | 0.06 | 0.64 | 0.09 | 0.96 | 0.01 | 0.95 | 0.01 |

**Supplementary table S8.** **The correlation analysis between the resting-state EEG wPLI strengths and the mean FA values of impaired WM tracts in patients with NMOSD (P value and r value).** Pearson correlation analysis or Spearman correlation analysis were used according to the normality distributions of the data, with age as covariant. P<0.05 with false discovery rate corrected was considered significant, marked with **bold font**.

|  | ATR_L | | ATR_R | | CST_L | | CGH_R | | Fmaj | | Fmin | | IFO_L | | IFO_R | | ILF_L | | ILF_R | |
| --- | --- | --- | --- | --- | --- | --- | --- | --- | --- | --- | --- | --- | --- | --- | --- | --- | --- | --- | --- | --- |
|  | P | r | P | r | P | r | P | r | P | r | P | r | P | r | P | r | P | r | P | r |
| Delta_Fz | 0.54 | -0.11 | 0.45 | -0.14 | 0.03 | -0.39 | 0.09 | -0.31 | 0.39 | -0.16 | 0.27 | -0.20 | 0.32 | -0.18 | 0.31 | -0.19 | 0.21 | -0.23 | 0.09 | -0.31 |
| Delta_C3 | 0.72 | -0.07 | 0.67 | -0.08 | 0.13 | -0.28 | 0.04 | -0.34 | 0.43 | -0.15 | 0.36 | -0.17 | 0.27 | -0.20 | 0.46 | -0.14 | 0.25 | -0.21 | 0.21 | -0.23 |
| Delta_C4 | 0.85 | -0.04 | 0.86 | -0.03 | 0.17 | -0.25 | 0.07 | -0.33 | 0.48 | -0.13 | 0.68 | -0.08 | 0.54 | -0.11 | 0.68 | -0.08 | 0.44 | -0.14 | 0.30 | -0.19 |
| Delta_Cz | 0.81 | 0.04 | 0.55 | 0.11 | 0.24 | -0.22 | 0.04 | -0.37 | 0.50 | -0.13 | 0.73 | -0.06 | 0.42 | -0.15 | 0.75 | -0.06 | 0.67 | -0.08 | 0.60 | -0.10 |
| Delta_Pz | 0.36 | -0.17 | 0.32 | -0.18 | 0.11 | -0.30 | 0.04 | -0.38 | 0.10 | -0.30 | 0.12 | -0.29 | 0.06 | -0.35 | 0.09 | -0.31 | 0.04 | -0.37 | 0.07 | -0.33 |
| Delta_Oz | 0.87 | -0.03 | 0.68 | -0.08 | 0.27 | -0.20 | 0.20 | -0.23 | 0.52 | -0.12 | 0.57 | -0.10 | 0.64 | -0.09 | 0.68 | -0.08 | 0.27 | -0.20 | 0.21 | -0.23 |
| Theta_Fz | 0.45 | -0.14 | 0.24 | -0.22 | 0.39 | -0.16 | 0.50 | -0.13 | 0.48 | -0.13 | 0.32 | -0.18 | 0.27 | -0.20 | 0.19 | -0.24 | 0.61 | -0.10 | 0.67 | -0.08 |
| Theta_C3 | 0.32 | -0.19 | 0.16 | -0.26 | 0.39 | -0.16 | 0.44 | -0.14 | 0.46 | -0.14 | 0.36 | -0.17 | 0.44 | -0.14 | 0.18 | -0.25 | 0.76 | -0.06 | 0.81 | -0.05 |
| Theta_C4 | 0.45 | -0.14 | 0.19 | -0.24 | 0.58 | -0.10 | 0.72 | -0.07 | 0.36 | -0.17 | 0.39 | -0.16 | 0.50 | -0.12 | 0.30 | -0.19 | 0.91 | -0.02 | 0.96 | -0.01 |
| Theta_Cz | 0.41 | -0.15 | 0.18 | -0.24 | 0.44 | -0.14 | 0.33 | -0.18 | 0.31 | -0.19 | 0.37 | -0.17 | 0.25 | -0.21 | 0.14 | -0.27 | 0.53 | -0.12 | 0.57 | -0.11 |
| Theta_Pz | 0.10 | -0.30 | 0.03 | -0.39 | 0.16 | -0.26 | 0.33 | -0.18 | 0.05 | -0.35 | 0.17 | -0.26 | 0.12 | -0.28 | 0.05 | -0.36 | 0.33 | -0.18 | 0.43 | -0.15 |
| Theta_Oz | 0.29 | -0.20 | 0.13 | -0.28 | 0.39 | -0.16 | 0.85 | -0.04 | 0.37 | -0.17 | 0.33 | -0.18 | 0.21 | -0.23 | 0.16 | -0.26 | 0.43 | -0.15 | 0.64 | -0.09 |
| Alpha_Fz | 0.53 | 0.12 | 0.68 | 0.08 | 0.70 | 0.07 | 0.51 | 0.12 | 0.56 | 0.11 | 0.83 | 0.04 | 0.28 | 0.20 | 0.33 | 0.18 | 0.51 | 0.12 | 0.84 | 0.04 |
| Alpha_C3 | 0.17 | 0.26 | 0.25 | 0.21 | 0.26 | 0.21 | 0.43 | 0.15 | 0.40 | 0.16 | 0.39 | 0.16 | 0.22 | 0.23 | 0.19 | 0.24 | 0.52 | 0.12 | 0.62 | 0.09 |
| Alpha_C4 | 0.09 | 0.31 | 0.06 | 0.34 | 0.07 | 0.33 | 0.60 | 0.10 | 0.16 | 0.26 | 0.16 | 0.26 | 0.07 | 0.33 | 0.06 | 0.35 | 0.36 | 0.17 | 0.38 | 0.16 |
| Alpha_Cz | 0.43 | 0.15 | 0.38 | 0.16 | 0.17 | 0.25 | 0.20 | 0.23 | 0.45 | 0.14 | 0.48 | 0.13 | 0.29 | 0.20 | 0.39 | 0.16 | 0.72 | 0.07 | 0.86 | 0.03 |
| Alpha_Pz | 0.64 | 0.09 | 0.81 | 0.05 | 0.75 | 0.06 | 0.22 | 0.23 | 0.64 | 0.09 | 0.84 | 0.04 | 0.27 | 0.20 | 0.34 | 0.18 | 0.41 | 0.15 | 0.67 | 0.08 |
| Alpha_Oz | 0.10 | 0.30 | 0.16 | 0.26 | 0.25 | 0.21 | 0.47 | 0.13 | 0.20 | 0.24 | 0.31 | 0.19 | 0.10 | 0.30 | 0.11 | 0.30 | 0.30 | 0.19 | 0.51 | 0.12 |
| Beta_Fz | 0.49 | -0.13 | 0.36 | -0.17 | 0.49 | 0.13 | 0.86 | 0.03 | 0.68 | 0.08 | 0.68 | -0.08 | 0.57 | -0.11 | 0.63 | -0.09 | 0.27 | -0.21 | 0.08 | -0.32 |
| Beta_C3 | 0.20 | -0.24 | 0.23 | -0.22 | 0.91 | -0.02 | 0.34 | 0.18 | 0.99 | 0.00 | 0.28 | -0.20 | 0.44 | -0.14 | 0.59 | -0.10 | 0.25 | -0.21 | 0.07 | -0.33 |
| Beta_C4 | 0.52 | -0.12 | 0.53 | -0.12 | 0.54 | 0.11 | 0.50 | 0.12 | 0.59 | 0.10 | 0.77 | -0.05 | 0.69 | -0.08 | 0.73 | -0.06 | 0.46 | -0.14 | 0.19 | -0.24 |
| Beta_Cz | 0.88 | -0.03 | 0.74 | -0.06 | 0.38 | 0.16 | 0.38 | 0.16 | 0.45 | 0.14 | 0.95 | -0.01 | 1.00 | 0.00 | 0.86 | 0.03 | 0.59 | -0.10 | 0.20 | -0.24 |
| Beta_Pz | 0.56 | 0.11 | 0.96 | 0.01 | 0.18 | 0.25 | 0.29 | 0.20 | 0.16 | 0.26 | 0.48 | 0.13 | 0.38 | 0.16 | 0.40 | 0.16 | 0.73 | 0.06 | 0.58 | -0.10 |
| Beta_Oz | 0.39 | 0.16 | 0.39 | 0.16 | 0.11 | 0.29 | 0.89 | 0.02 | 0.06 | 0.34 | 0.23 | 0.22 | 0.22 | 0.23 | 0.26 | 0.21 | 0.54 | 0.11 | 0.95 | -0.01 |
| Gamma_Fz | 0.45 | -0.14 | 0.71 | -0.07 | 0.53 | -0.12 | 0.11 | -0.29 | 0.74 | -0.06 | 0.91 | 0.02 | 0.38 | -0.16 | 0.22 | -0.23 | 0.29 | -0.19 | 0.57 | -0.11 |
| Gamma_C3 | 0.88 | -0.03 | 0.90 | 0.02 | 0.83 | -0.04 | 0.25 | 0.22 | 0.24 | -0.22 | 0.86 | 0.03 | 0.42 | -0.15 | 0.51 | -0.12 | 0.59 | -0.10 | 0.95 | 0.01 |
| Gamma_C4 | 0.21 | 0.23 | 0.11 | 0.29 | 0.53 | 0.12 | 0.68 | -0.08 | 0.64 | 0.09 | 0.29 | 0.20 | 0.86 | 0.03 | 0.67 | 0.08 | 0.98 | 0.01 | 0.79 | -0.05 |
| Gamma_Cz | 0.47 | 0.13 | 0.50 | 0.12 | 0.83 | -0.04 | 0.19 | -0.24 | 0.60 | -0.10 | 0.79 | 0.05 | 0.51 | -0.12 | 0.47 | -0.13 | 0.39 | -0.16 | 0.42 | -0.15 |
| Gamma_Pz | 0.58 | -0.10 | 0.43 | -0.15 | 0.20 | -0.24 | 0.02 | -0.42 | 0.39 | -0.16 | 0.32 | -0.18 | 0.05 | -0.36 | 0.03 | -0.39 | 0.02 | -0.43 | 0.03 | -0.39 |
| Gamma_Oz | 0.53 | -0.12 | 0.81 | -0.05 | 0.63 | -0.09 | 0.28 | -0.20 | 0.26 | -0.21 | 0.71 | -0.07 | 0.51 | -0.12 | 0.25 | -0.21 | 0.67 | -0.08 | 0.33 | -0.18 |

**Supplementary table S9. The comparisons of the identified EEG indices among the NMOSD subgroups, according to patients with AQP4-antibody positive/negative, different disease phenotype and different preventive therapies.** The statistical method was Unpaired T test, Mann-Whitney test or Kruskal-Wallis test, and P<0.05 was considered significant, marked with **bold font**.

| EEG index | AQP4 antibody | | | Different disease phenotype | | | | Different preventive therapy | | | |
| --- | --- | --- | --- | --- | --- | --- | --- | --- | --- | --- | --- |
|  | AQP4-positive | AQP4-negative | P | ON | TM | ON+TM | P | MMF/AZA | RTX | Other | P |
| Delta/Theta-C3 | 2.0±2.1 | 3.1±2.0 | 0.29 | 4.2±0.2 | 1.7±2.5 | 2.1±1.9 | 0.11 | 2.5±2.0 | 1.7±2.0 | 0.03±3.4 | 0.36 |
| Delta/Theta-Cz | 2.0±2.0 | 3.6±1.8 | 0.09 | 4.0±1.5 | 1.8±2.4 | 2.2±1.9 | 0.21 | 2.5±2.2 | 1.6±1.7 | 1.4±2.6 | 0.27 |
| Theta/Beta-Cz | 5.8±3.7 | 4.6±2.6 | 0.62 | 2.6±4.0 | 6.9±4.2 | 5.3±2.6 | 0.24 | 5.2±3.1 | 7.4±4.7 | 3.7±1.0 | 0.34 |
| COH-theta-Fz | 0.30±0.05 | 0.30±0.10 | 0.75 | 0.29±0.05 | 0.33±0.07 | 0.29±0.06 | 0.47 | 0.29±0.06 | 0.33±0.07 | 0.31±0.02 | 0.30 |
| COH-theta-Cz | 0.20±0.06 | 0.19±0.03 | 0.76 | 0.19±0.02 | 0.21±0.08 | 0.18±0.04 | 0.63 | 0.19±0.06 | 0.2±0.07 | 0.15±0.06 | 0.57 |
| COH-Gamma-Cz | 0.14±0.03 | 0.15±0.09 | 0.42 | 0.14±0.02 | 0.14±0.06 | 0.13±0.03 | 0.92 | 0.14±0.05 | 0.14±0.03 | 0.11±0.05 | 0.73 |
| PLV-theta-Fz | 0.42±0.06 | 0.41±0.10 | 0.68 | 0.40±0.06 | 0.45±0.08 | 0.41±0.05 | 0.26 | 0.41±0.06 | 0.44±0.07 | 0.41±0.02 | 0.36 |
| PLV-theta-C4 | 0.29±0.04 | 0.33±0.07 | 0.13 | 0.26±0.03 | 0.29±0.05 | 0.28±0.03 | 0.43 | 0.30±0.05 | 0.31±0.05 | 0.26±0.05 | 0.47 |
| PLV-theta-Cz | 0.31±0.06 | 0.31±0.09 | 0.74 | 0.31±0.02 | 0.34±0.10 | 0.29±0.04 | 0.32 | 0.31±0.06 | 0.32±0.08 | 0.25±0.07 | 0.53 |
| PLV-theta-Oz | 0.41±0.05 | 0.42±0.10 | 0.48 | 0.38±0.07 | 0.45±0.06 | 0.40±0.05 | 0.14 | 0.41±0.06 | 0.42±0.06 | 0.40±0.03 | 0.70 |
| PLV-Gamma-Cz | 0.24±0.04 | 0.25±0.10 | 0.45 | 0.24±0.03 | 0.24±0.07 | 0.23±0.04 | 0.96 | 0.24±0.05 | 0.24±0.04 | 0.21±0.05 | 0.62 |

**Supplementary table S10. Correlation analysis between the identified EEG indices and disease characteristics** **(the number of attacks, disease duration, the EDSS score, and the serum GFAP level) in patients with NMOSD (P value and r value results).** Pearson correlation analysis or Spearman correlation analysis were used according to the normality distributions of the data, with age as covariates. P<0.05 was considered significant, marked with **bold font**.

| Relative power | Correlations between EEG index and the number of attacks | | Correlations between EEG index and the disease duration (year) | | Correlations between EEG index and the EDSS score | | Correlations between EEG index and the serum GFAP level | |
| --- | --- | --- | --- | --- | --- | --- | --- | --- |
|  | P value | r value | P value | r value | P value | r value | P value | r value |
| Delta/Theta-C3 | 0.65 | -0.09 | 1.00 | 0.00 | 0.44 | 0.15 | 0.53 | 0.13 |
| Delta/Theta-Cz | 0.62 | -0.09 | 0.87 | 0.03 | 0.78 | 0.05 | 0.14 | 0.30 |
| Theta/Beta-Cz | 0.79 | 0.05 | 0.86 | 0.03 | 0.67 | -0.08 | 0.83 | 0.05 |
| COH-theta-Fz | 0.17 | -0.25 | 0.07 | -0.33 | 0.31 | -0.19 | 0.14 | -0.30 |
| COH-theta-Cz | 0.08 | -0.31 | 0.12 | -0.28 | **0.02** | **-0.42** | 0.25 | -0.24 |
| COH-Gamma-Cz | 0.14 | -0.27 | 0.45 | -0.14 | **0.02** | **-0.41** | 0.46 | -0.16 |
| PLV-theta-Fz | 0.17 | -0.25 | 0.09 | -0.31 | 0.44 | -0.15 | **0.03** | **-0.43** |
| PLV-theta-C4 | 0.11 | -0.29 | 0.33 | -0.18 | 0.12 | -0.28 | 0.50 | -0.14 |
| PLV-theta-Cz | **0.01** | **-0.44** | 0.05 | -0.36 | **0.01** | **-0.47** | 0.21 | -0.26 |
| PLV-theta-Oz | 0.11 | -0.30 | 0.26 | -0.21 | 0.43 | -0.15 | 0.08 | -0.36 |
| PLV-Gamma-Cz | 0.05 | -0.36 | 0.44 | -0.14 | **0.01** | **-0.47** | 0.40 | -0.18 |

**Supplementary table S11. Correlation analysis between the identified EEG indices and cognitive function in patients with NMOSD (P value and r value results).** Pearson correlation analysis or Spearman correlation analysis were used according to the normality distributions of the data, with age and education level as covariates. P<0.05 was considered significant, marked with **bold font**.

| Relative power | Correlations between EEG index and the PASAT | | Correlations between EEG index and the SDMT | |
| --- | --- | --- | --- | --- |
|  | P value | r value | P value | r value |
| Delta/Theta-C3 | 0.51 | -0.14 | 0.24 | -0.25 |
| Delta/Theta-Cz | 0.06 | -0.38 | 0.26 | -0.24 |
| Theta/Beta-Cz | 0.50 | 0.14 | **0.03** | **0.44** |
| COH-theta-Fz | 0.32 | 0.21 | 0.17 | 0.29 |
| COH-theta-Cz | 0.68 | 0.09 | 0.30 | 0.22 |
| COH-Gamma-Cz | 0.16 | 0.29 | 0.15 | 0.30 |
| PLV-theta-Fz | 0.19 | 0.27 | 0.16 | 0.29 |
| PLV-theta-C4 | 0.56 | 0.12 | 0.05 | 0.40 |
| PLV-theta-Cz | 0.24 | 0.25 | 0.33 | 0.21 |
| PLV-theta-Oz | 0.28 | 0.23 | **0.03** | **0.46** |
| PLV-Gamma-Cz | 0.17 | 0.29 | 0.19 | 0.28 |
