# Supplementary material for: EEG biomarkers of microstructural damage in normal-appearing white matter among patients with neuromyelitis optica spectrum disorder: A DTI-EEG combined study
Source: Front Immunol. 2026 Feb 25;17:1676066. doi: 10.3389/fimmu.2026.1676066 (PMC12975549; doi:10.3389/fimmu.2026.1676066)
Supplement: SUPPLEMENTARY FILE 3 — The heatmap of r values and detailed statistical results of the correlation analysis between rs-EEG indices and the mean RD values of impaired WM tracts. [file Supplementaryfile3.docx]

**Supplementary material 3**


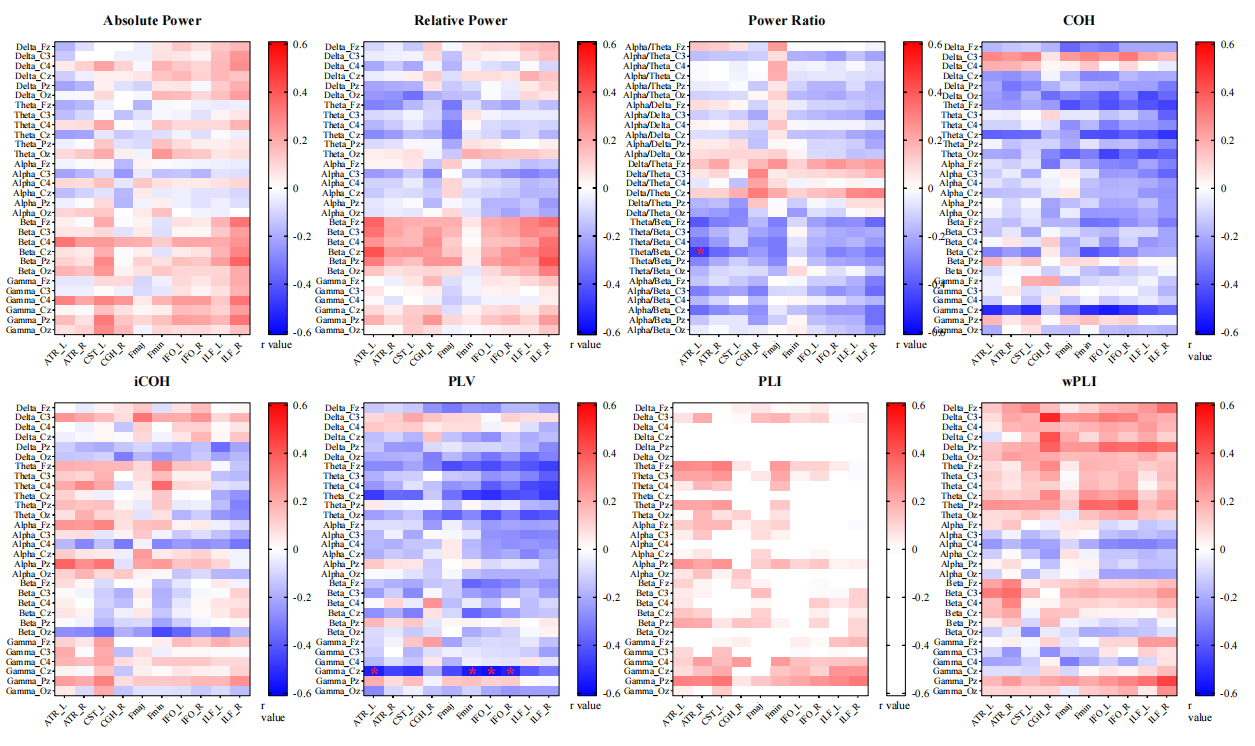


Figure S2. Heatmaps of the r values in the correlation analysis between the EEG metrics and the radial diffusivity (RD) values of the impaired white matter tracts in patients with NMOSD. The correlations with statistical significance are marked with * (P<0.05 after false discovery rate correction). COH, coherence; iCOH, imaginary coherence; PLV, phase locking value; PLI, phase lag index; wPLI, weighted phase lag index; ATR, anterior thalamic radiation; CST, corticospinal tract; CGH, cingulum (hippocampus); Fmaj, forceps major; Fmin, forceps minor; IFO, inferior fronto-occipital fasciculus; ILF, inferior longitudinal fasciculus; L, left; R, right.

After FDR correction, the theta/beta power ratio of the Cz channel was found to be correlated with the mean RD values of ATR_L (r=-0.48). Also, The gamma-PLV strength of the Cz channel was found to be significantly correlated with the RD values of multiple WM tracts, including ATR_L (r=-0.54), Fmin (r=0.55), IFO_L (r=-0.57), and IFO_R (r=-0.53). The detailed statistical data were displayed as follows (supplementary table S20-27).

**Supplementary table S20.** **The correlation analysis between the resting-state EEG absolute power and the mean RD values of impaired WM tracts in patients with NMOSD (P value and r value).** Pearson correlation analysis or Spearman correlation analysis were used according to the normality distributions of the data, with age as covariant. P<0.05 with false discovery rate corrected was considered significant, marked with **bold font**.

|  | ATR_L | | ATR_R | | CST_L | | CGH_R | | Fmaj | | Fmin | | IFO_L | | IFO_R | | ILF_L | | ILF_R | |
| --- | --- | --- | --- | --- | --- | --- | --- | --- | --- | --- | --- | --- | --- | --- | --- | --- | --- | --- | --- | --- |
|  | P | r | P | r | P | r | P | r | P | r | P | r | P | r | P | r | P | r | P | r |
| Delta_Fz | 0.34 | -0.18 | 0.66 | -0.08 | 0.99 | 0.00 | 0.98 | 0.00 | 0.82 | -0.04 | 0.73 | 0.06 | 0.50 | 0.13 | 0.72 | 0.07 | 0.49 | 0.13 | 0.35 | 0.17 |
| Delta_C3 | 0.49 | -0.13 | 0.99 | 0.00 | 0.98 | 0.00 | 0.82 | -0.04 | 0.84 | -0.04 | 0.71 | 0.07 | 0.74 | 0.06 | 0.88 | 0.03 | 0.40 | 0.16 | 0.18 | 0.25 |
| Delta_C4 | 0.79 | 0.05 | 0.55 | 0.11 | 0.24 | 0.22 | 0.54 | 0.11 | 0.91 | -0.02 | 0.32 | 0.18 | 0.48 | 0.13 | 0.58 | 0.10 | 0.36 | 0.17 | 0.14 | 0.27 |
| Delta_Cz | 0.54 | -0.12 | 0.83 | -0.04 | 0.84 | 0.04 | 0.82 | 0.04 | 0.74 | 0.06 | 0.46 | 0.14 | 0.36 | 0.17 | 0.57 | 0.11 | 0.37 | 0.17 | 0.45 | 0.14 |
| Delta_Pz | 0.25 | -0.22 | 0.33 | -0.18 | 0.82 | 0.04 | 0.70 | -0.07 | 0.56 | -0.11 | 0.77 | 0.06 | 0.74 | 0.06 | 0.92 | -0.02 | 0.52 | 0.12 | 0.29 | 0.19 |
| Delta_Oz | 0.91 | -0.02 | 0.92 | -0.02 | 0.62 | 0.09 | 0.99 | 0.00 | 0.92 | -0.02 | 0.35 | 0.17 | 0.39 | 0.16 | 0.62 | 0.09 | 0.41 | 0.15 | 0.22 | 0.23 |
| Theta_Fz | 0.28 | -0.20 | 0.36 | -0.17 | 0.82 | -0.04 | 0.98 | 0.00 | 0.34 | -0.18 | 0.93 | -0.02 | 0.95 | 0.01 | 0.83 | -0.04 | 0.80 | -0.05 | 0.84 | -0.04 |
| Theta_C3 | 0.40 | -0.16 | 0.61 | -0.10 | 0.98 | -0.01 | 0.43 | -0.15 | 0.72 | -0.07 | 0.90 | -0.02 | 0.88 | -0.03 | 0.71 | -0.07 | 0.86 | 0.03 | 0.74 | 0.06 |
| Theta_C4 | 0.70 | 0.07 | 0.65 | 0.08 | 0.26 | 0.21 | 0.96 | 0.01 | 0.87 | -0.03 | 0.46 | 0.14 | 0.61 | 0.09 | 0.64 | 0.09 | 0.62 | 0.09 | 0.34 | 0.18 |
| Theta_Cz | 0.21 | -0.23 | 0.24 | -0.22 | 0.74 | -0.06 | 0.50 | -0.13 | 0.61 | -0.10 | 0.66 | -0.08 | 0.89 | -0.03 | 0.67 | -0.08 | 0.94 | -0.01 | 0.83 | -0.04 |
| Theta_Pz | 0.82 | -0.04 | 0.96 | -0.01 | 0.49 | 0.13 | 0.73 | -0.07 | 0.63 | -0.09 | 0.42 | 0.15 | 0.68 | 0.08 | 0.83 | 0.04 | 0.83 | 0.04 | 0.73 | 0.06 |
| Theta_Oz | 0.64 | 0.09 | 0.49 | 0.13 | 0.28 | 0.20 | 0.81 | 0.04 | 0.86 | -0.03 | 0.18 | 0.25 | 0.43 | 0.15 | 0.49 | 0.13 | 0.76 | 0.06 | 0.68 | 0.08 |
| Alpha_Fz | 0.87 | -0.03 | 0.95 | -0.01 | 0.93 | 0.02 | 0.36 | -0.17 | 0.88 | 0.03 | 0.96 | -0.01 | 0.93 | -0.02 | 0.88 | -0.03 | 0.82 | -0.04 | 0.77 | -0.05 |
| Alpha_C3 | 0.24 | -0.22 | 0.35 | -0.17 | 0.62 | -0.09 | 0.17 | -0.25 | 0.78 | -0.05 | 0.42 | -0.15 | 0.47 | -0.14 | 0.35 | -0.18 | 0.72 | -0.07 | 0.65 | -0.08 |
| Alpha_C4 | 0.62 | 0.09 | 0.67 | 0.08 | 0.41 | 0.15 | 0.76 | -0.06 | 0.47 | 0.13 | 0.69 | 0.07 | 0.79 | 0.05 | 0.78 | 0.05 | 0.72 | 0.07 | 0.61 | 0.10 |
| Alpha_Cz | 0.38 | -0.16 | 0.55 | -0.11 | 0.70 | -0.07 | 0.37 | -0.17 | 0.77 | 0.05 | 0.77 | -0.05 | 0.81 | -0.04 | 0.69 | -0.07 | 0.72 | -0.07 | 0.60 | -0.10 |
| Alpha_Pz | 0.81 | -0.04 | 0.95 | -0.01 | 0.63 | 0.09 | 0.34 | -0.18 | 0.87 | -0.03 | 0.83 | 0.04 | 0.84 | -0.04 | 0.72 | -0.07 | 0.84 | -0.04 | 0.67 | -0.08 |
| Alpha_Oz | 0.58 | 0.10 | 0.52 | 0.12 | 0.48 | 0.13 | 0.99 | 0.00 | 0.79 | 0.05 | 0.43 | 0.15 | 0.99 | 0.00 | 0.86 | -0.03 | 0.82 | -0.04 | 1.00 | 0.00 |
| Beta_Fz | 0.83 | 0.04 | 0.94 | -0.01 | 0.29 | 0.20 | 0.95 | -0.01 | 0.86 | 0.03 | 0.73 | -0.07 | 0.72 | 0.07 | 0.80 | 0.05 | 0.34 | 0.18 | 0.07 | 0.33 |
| Beta_C3 | 0.75 | 0.06 | 0.74 | 0.06 | 0.62 | 0.09 | 0.71 | -0.07 | 0.80 | 0.05 | 0.66 | -0.08 | 0.91 | 0.02 | 0.90 | 0.02 | 0.48 | 0.13 | 0.21 | 0.23 |
| Beta_C4 | 0.07 | 0.33 | 0.18 | 0.24 | 0.19 | 0.24 | 0.28 | 0.20 | 0.30 | 0.19 | 0.52 | 0.12 | 0.27 | 0.21 | 0.27 | 0.21 | 0.28 | 0.20 | 0.16 | 0.26 |
| Beta_Cz | 0.69 | 0.07 | 0.81 | 0.05 | 0.31 | 0.19 | 0.95 | -0.01 | 0.84 | 0.04 | 0.71 | -0.07 | 0.79 | 0.05 | 0.70 | 0.07 | 0.33 | 0.18 | 0.11 | 0.29 |
| Beta_Pz | 0.57 | 0.11 | 0.71 | 0.07 | 0.13 | 0.28 | 0.94 | 0.01 | 0.55 | 0.11 | 0.71 | 0.07 | 0.41 | 0.15 | 0.50 | 0.13 | 0.22 | 0.23 | 0.04 | 0.37 |
| Beta_Oz | 0.32 | 0.18 | 0.42 | 0.15 | 0.18 | 0.25 | 0.87 | 0.03 | 0.69 | 0.07 | 0.50 | 0.13 | 0.58 | 0.10 | 0.58 | 0.10 | 0.59 | 0.10 | 0.28 | 0.20 |
| Gamma_Fz | 0.75 | -0.06 | 0.76 | -0.06 | 0.65 | 0.09 | 0.64 | 0.09 | 0.59 | -0.10 | 0.85 | -0.04 | 0.64 | 0.09 | 0.76 | 0.06 | 0.57 | 0.11 | 0.27 | 0.20 |
| Gamma_C3 | 0.82 | -0.04 | 0.83 | -0.04 | 0.96 | 0.01 | 0.84 | -0.04 | 0.62 | -0.09 | 0.74 | -0.06 | 0.70 | 0.07 | 0.88 | 0.03 | 0.72 | 0.07 | 0.34 | 0.18 |
| Gamma_C4 | 0.10 | 0.30 | 0.27 | 0.21 | 0.11 | 0.29 | 0.18 | 0.25 | 0.77 | 0.05 | 0.32 | 0.18 | 0.20 | 0.24 | 0.19 | 0.24 | 0.49 | 0.13 | 0.07 | 0.33 |
| Gamma_Cz | 0.93 | 0.02 | 0.98 | 0.01 | 0.42 | 0.15 | 0.71 | 0.07 | 0.93 | 0.02 | 0.85 | 0.04 | 0.38 | 0.16 | 0.41 | 0.15 | 0.56 | 0.11 | 0.21 | 0.23 |
| Gamma_Pz | 0.38 | 0.16 | 0.71 | 0.07 | 0.08 | 0.32 | 0.44 | 0.14 | 0.58 | 0.10 | 0.43 | 0.15 | 0.22 | 0.23 | 0.24 | 0.22 | 0.36 | 0.17 | 0.07 | 0.33 |
| Gamma_Oz | 0.72 | 0.07 | 0.55 | 0.11 | 0.13 | 0.28 | 0.35 | 0.17 | 0.87 | -0.03 | 0.61 | 0.10 | 0.69 | 0.08 | 0.62 | 0.09 | 0.75 | 0.06 | 0.44 | 0.15 |

**Supplementary table S21.** **The correlation analysis between the resting-state EEG relative power and the mean RD values of impaired WM tracts in patients with NMOSD (P value and r value).** Pearson correlation analysis or Spearman correlation analysis were used according to the normality distributions of the data, with age as covariant. P<0.05 with false discovery rate corrected was considered significant, marked with **bold font**.

|  | ATR_L | | ATR_R | | CST_L | | CGH_R | | Fmaj | | Fmin | | IFO_L | | IFO_R | | ILF_L | | ILF_R | |
| --- | --- | --- | --- | --- | --- | --- | --- | --- | --- | --- | --- | --- | --- | --- | --- | --- | --- | --- | --- | --- |
|  | P | r | P | r | P | r | P | r | P | r | P | r | P | r | P | r | P | r | P | r |
| Delta_Fz | 0.58 | -0.10 | 0.81 | -0.05 | 0.50 | -0.13 | 0.54 | 0.12 | 0.93 | -0.02 | 0.72 | 0.07 | 0.60 | 0.10 | 0.63 | 0.09 | 0.42 | 0.15 | 0.36 | 0.17 |
| Delta_C3 | 0.80 | -0.05 | 0.82 | -0.04 | 0.88 | -0.03 | 0.62 | 0.09 | 0.42 | -0.15 | 0.74 | 0.06 | 0.89 | 0.03 | 0.92 | 0.02 | 0.60 | 0.10 | 0.43 | 0.15 |
| Delta_C4 | 0.26 | -0.21 | 0.48 | -0.13 | 0.25 | -0.21 | 0.86 | -0.03 | 0.34 | -0.18 | 0.86 | -0.03 | 0.67 | -0.08 | 0.57 | -0.11 | 0.96 | -0.01 | 0.58 | -0.10 |
| Delta_Cz | 0.79 | -0.05 | 0.85 | -0.03 | 0.83 | 0.04 | 0.52 | 0.12 | 0.66 | -0.08 | 0.66 | 0.08 | 0.65 | 0.08 | 0.73 | 0.06 | 0.30 | 0.19 | 0.47 | 0.14 |
| Delta_Pz | 0.23 | -0.22 | 0.28 | -0.20 | 0.31 | -0.19 | 0.69 | 0.07 | 0.47 | -0.13 | 0.62 | -0.09 | 0.97 | -0.01 | 0.78 | -0.05 | 0.80 | 0.05 | 0.55 | 0.11 |
| Delta_Oz | 0.17 | -0.25 | 0.16 | -0.26 | 0.06 | -0.34 | 0.30 | -0.19 | 0.38 | -0.16 | 0.41 | -0.15 | 0.95 | -0.01 | 0.73 | -0.06 | 0.63 | 0.09 | 0.74 | 0.06 |
| Theta_Fz | 0.10 | -0.30 | 0.13 | -0.28 | 0.37 | -0.17 | 0.54 | -0.11 | 0.07 | -0.33 | 0.67 | -0.08 | 0.35 | -0.18 | 0.22 | -0.22 | 0.54 | -0.11 | 0.40 | -0.16 |
| Theta_C3 | 0.60 | -0.10 | 0.66 | -0.08 | 0.85 | -0.03 | 0.59 | -0.10 | 0.17 | -0.25 | 0.71 | 0.07 | 0.88 | -0.03 | 0.77 | -0.05 | 0.81 | -0.05 | 0.80 | -0.05 |
| Theta_C4 | 0.35 | -0.18 | 0.50 | -0.13 | 0.76 | -0.06 | 0.48 | -0.13 | 0.08 | -0.32 | 0.96 | 0.01 | 0.62 | -0.09 | 0.56 | -0.11 | 0.58 | -0.10 | 0.57 | -0.11 |
| Theta_Cz | 0.13 | -0.28 | 0.18 | -0.24 | 0.38 | -0.16 | 0.30 | -0.19 | 0.07 | -0.33 | 0.74 | -0.06 | 0.59 | -0.10 | 0.36 | -0.17 | 0.58 | -0.10 | 0.32 | -0.18 |
| Theta_Pz | 0.62 | -0.09 | 0.72 | -0.07 | 0.92 | -0.02 | 0.93 | -0.02 | 0.26 | -0.21 | 0.70 | 0.07 | 0.81 | 0.05 | 0.95 | 0.01 | 0.99 | 0.00 | 0.82 | 0.04 |
| Theta_Oz | 0.84 | 0.04 | 0.80 | 0.05 | 0.71 | 0.07 | 0.87 | -0.03 | 0.50 | -0.13 | 0.32 | 0.19 | 0.39 | 0.16 | 0.46 | 0.14 | 0.48 | 0.13 | 0.59 | 0.10 |
| Alpha_Fz | 0.89 | 0.03 | 0.87 | 0.03 | 0.75 | 0.06 | 0.45 | -0.14 | 0.51 | 0.12 | 0.90 | -0.02 | 0.60 | -0.10 | 0.59 | -0.10 | 0.40 | -0.16 | 0.44 | -0.14 |
| Alpha_C3 | 0.20 | -0.24 | 0.27 | -0.21 | 0.48 | -0.13 | 0.13 | -0.28 | 0.99 | 0.00 | 0.38 | -0.16 | 0.25 | -0.21 | 0.15 | -0.27 | 0.10 | -0.30 | 0.16 | -0.26 |
| Alpha_C4 | 0.57 | -0.11 | 0.55 | -0.11 | 0.79 | -0.05 | 0.43 | -0.15 | 0.60 | 0.10 | 0.79 | -0.05 | 0.69 | -0.08 | 0.56 | -0.11 | 0.36 | -0.17 | 0.43 | -0.15 |
| Alpha_Cz | 0.48 | -0.13 | 0.66 | -0.08 | 0.63 | -0.09 | 0.53 | -0.12 | 0.60 | 0.10 | 0.65 | -0.09 | 0.54 | -0.11 | 0.47 | -0.13 | 0.33 | -0.18 | 0.36 | -0.17 |
| Alpha_Pz | 0.74 | -0.06 | 0.85 | -0.04 | 0.96 | -0.01 | 0.37 | -0.17 | 0.93 | -0.02 | 0.81 | -0.05 | 0.34 | -0.18 | 0.33 | -0.18 | 0.45 | -0.14 | 0.19 | -0.24 |
| Alpha_Oz | 0.69 | -0.07 | 0.78 | -0.05 | 0.75 | -0.06 | 0.95 | 0.01 | 0.64 | 0.09 | 0.71 | -0.07 | 0.35 | -0.17 | 0.23 | -0.22 | 0.40 | -0.16 | 0.25 | -0.21 |
| Beta_Fz | 0.04 | 0.37 | 0.26 | 0.21 | 0.30 | 0.19 | 0.31 | 0.19 | 0.32 | 0.19 | 0.74 | 0.06 | 0.29 | 0.20 | 0.20 | 0.23 | 0.10 | 0.30 | 0.06 | 0.35 |
| Beta_C3 | 0.06 | 0.34 | 0.23 | 0.22 | 0.36 | 0.17 | 0.19 | 0.24 | 0.37 | 0.17 | 0.91 | 0.02 | 0.34 | 0.18 | 0.23 | 0.22 | 0.11 | 0.29 | 0.14 | 0.27 |
| Beta_C4 | 0.16 | 0.26 | 0.34 | 0.18 | 0.35 | 0.17 | 0.14 | 0.27 | 0.52 | 0.12 | 0.94 | 0.01 | 0.44 | 0.15 | 0.38 | 0.16 | 0.23 | 0.22 | 0.06 | 0.34 |
| Beta_Cz | 0.02 | 0.41 | 0.17 | 0.25 | 0.16 | 0.26 | 0.19 | 0.24 | 0.44 | 0.14 | 0.81 | 0.05 | 0.30 | 0.19 | 0.15 | 0.26 | 0.10 | 0.30 | 0.05 | 0.36 |
| Beta_Pz | 0.09 | 0.31 | 0.30 | 0.19 | 0.43 | 0.15 | 0.17 | 0.25 | 0.17 | 0.25 | 0.85 | 0.04 | 0.22 | 0.23 | 0.13 | 0.27 | 0.19 | 0.24 | 0.02 | 0.42 |
| Beta_Oz | 0.45 | 0.14 | 0.71 | 0.07 | 0.62 | 0.09 | 0.58 | 0.10 | 0.66 | 0.08 | 0.75 | -0.06 | 0.63 | 0.09 | 0.38 | 0.16 | 0.30 | 0.19 | 0.09 | 0.31 |
| Gamma_Fz | 0.99 | 0.00 | 0.82 | -0.04 | 0.76 | 0.06 | 0.37 | 0.17 | 0.65 | -0.08 | 0.87 | -0.03 | 0.76 | 0.06 | 0.80 | 0.05 | 0.64 | 0.09 | 0.47 | 0.14 |
| Gamma_C3 | 0.74 | 0.06 | 0.99 | 0.00 | 0.93 | 0.02 | 0.81 | 0.04 | 0.54 | -0.11 | 0.89 | -0.03 | 0.86 | 0.03 | 0.84 | 0.04 | 0.99 | 0.00 | 0.78 | 0.05 |
| Gamma_C4 | 0.94 | 0.01 | 0.72 | -0.07 | 0.99 | 0.00 | 0.83 | -0.04 | 0.47 | -0.13 | 0.66 | -0.08 | 0.86 | -0.03 | 0.88 | -0.03 | 0.84 | -0.04 | 0.82 | 0.04 |
| Gamma_Cz | 0.64 | 0.09 | 0.82 | 0.04 | 0.52 | 0.12 | 0.46 | 0.14 | 0.69 | -0.08 | 0.86 | 0.03 | 0.67 | 0.08 | 0.61 | 0.10 | 0.78 | 0.05 | 0.45 | 0.14 |
| Gamma_Pz | 0.32 | 0.19 | 0.58 | 0.10 | 0.50 | 0.13 | 0.18 | 0.25 | 0.58 | 0.10 | 0.76 | 0.06 | 0.44 | 0.14 | 0.35 | 0.17 | 0.57 | 0.11 | 0.19 | 0.24 |
| Gamma_Oz | 0.94 | 0.01 | 0.94 | 0.01 | 0.52 | 0.12 | 0.65 | 0.08 | 0.88 | -0.03 | 0.95 | -0.01 | 0.85 | 0.03 | 0.78 | 0.05 | 0.74 | 0.06 | 0.46 | 0.14 |

**Supplementary table S22.** **The correlation analysis between the resting-state EEG absolute power ratio and the mean RD values of impaired WM tracts in patients with NMOSD (P value and r value).** Pearson correlation analysis or Spearman correlation analysis were used according to the normality distributions of the data, with age as covariant. P<0.05 with false discovery rate corrected was considered significant, marked with **bold font**.

|  | ATR_L | | ATR_R | | CST_L | | CGH_R | | Fmaj | | Fmin | | IFO_L | | IFO_R | | ILF_L | | ILF_R | |
| --- | --- | --- | --- | --- | --- | --- | --- | --- | --- | --- | --- | --- | --- | --- | --- | --- | --- | --- | --- | --- |
|  | P | r | P | r | P | r | P | r | P | r | P | r | P | r | P | r | P | r | P | r |
| Alpha/Theta_Fz | 0.47 | 0.14 | 0.52 | 0.12 | 0.72 | 0.07 | 0.63 | -0.09 | 0.19 | 0.24 | 0.95 | -0.01 | 0.97 | -0.01 | 0.96 | -0.01 | 0.99 | 0.00 | 0.88 | -0.03 |
| Alpha/Theta_C3 | 0.39 | -0.16 | 0.46 | -0.14 | 0.46 | -0.14 | 0.51 | -0.12 | 0.67 | 0.08 | 0.31 | -0.19 | 0.29 | -0.20 | 0.20 | -0.24 | 0.43 | -0.15 | 0.24 | -0.22 |
| Alpha/Theta_C4 | 0.97 | -0.01 | 0.98 | 0.00 | 0.84 | -0.04 | 1.00 | 0.00 | 0.28 | 0.20 | 0.81 | -0.04 | 0.87 | -0.03 | 0.74 | -0.06 | 0.92 | -0.02 | 0.65 | -0.08 |
| Alpha/Theta_Cz | 0.94 | -0.01 | 0.99 | 0.00 | 0.88 | -0.03 | 0.95 | -0.01 | 0.30 | 0.19 | 0.69 | -0.07 | 0.74 | -0.06 | 0.74 | -0.06 | 0.66 | -0.08 | 0.61 | -0.09 |
| Alpha/Theta_Pz | 0.77 | -0.05 | 0.84 | -0.04 | 0.94 | -0.01 | 0.49 | -0.13 | 0.73 | 0.06 | 0.54 | -0.11 | 0.34 | -0.18 | 0.33 | -0.18 | 0.61 | -0.09 | 0.33 | -0.18 |
| Alpha/Theta_Oz | 0.56 | -0.11 | 0.60 | -0.10 | 0.62 | -0.09 | 0.92 | 0.02 | 0.55 | 0.11 | 0.36 | -0.17 | 0.31 | -0.19 | 0.26 | -0.21 | 0.40 | -0.16 | 0.36 | -0.17 |
| Alpha/Delta_Fz | 0.68 | 0.08 | 0.76 | 0.06 | 0.93 | 0.02 | 0.55 | -0.11 | 0.77 | 0.05 | 0.78 | -0.05 | 0.57 | -0.11 | 0.56 | -0.11 | 0.50 | -0.13 | 0.38 | -0.16 |
| Alpha/Delta_C3 | 0.51 | -0.12 | 0.63 | -0.09 | 0.53 | -0.12 | 0.26 | -0.21 | 0.73 | 0.07 | 0.45 | -0.14 | 0.36 | -0.17 | 0.30 | -0.19 | 0.29 | -0.20 | 0.13 | -0.28 |
| Alpha/Delta_C4 | 0.88 | 0.03 | 0.94 | 0.01 | 0.80 | 0.05 | 0.62 | -0.09 | 0.47 | 0.14 | 0.90 | -0.02 | 0.87 | -0.03 | 0.88 | -0.03 | 0.85 | -0.03 | 0.73 | -0.07 |
| Alpha/Delta_Cz | 0.68 | -0.08 | 0.68 | -0.08 | 0.51 | -0.12 | 0.45 | -0.14 | 0.75 | 0.06 | 0.41 | -0.15 | 0.38 | -0.16 | 0.41 | -0.15 | 0.25 | -0.21 | 0.20 | -0.24 |
| Alpha/Delta_Pz | 0.80 | 0.05 | 0.79 | 0.05 | 0.68 | 0.08 | 0.42 | -0.15 | 0.92 | 0.02 | 0.97 | -0.01 | 0.58 | -0.10 | 0.60 | -0.10 | 0.53 | -0.12 | 0.25 | -0.21 |
| Alpha/Delta_Oz | 0.67 | 0.08 | 0.64 | 0.09 | 0.59 | 0.10 | 0.64 | 0.09 | 0.54 | 0.11 | 0.87 | 0.03 | 0.63 | -0.09 | 0.60 | -0.10 | 0.43 | -0.15 | 0.44 | -0.14 |
| Delta/Theta_Fz | 0.44 | 0.14 | 0.26 | 0.21 | 0.69 | 0.07 | 0.37 | 0.17 | 0.15 | 0.26 | 0.56 | 0.11 | 0.25 | 0.21 | 0.17 | 0.25 | 0.25 | 0.21 | 0.19 | 0.24 |
| Delta/Theta_C3 | 0.75 | 0.06 | 0.61 | 0.10 | 0.98 | 0.01 | 0.11 | 0.29 | 0.80 | 0.05 | 0.68 | 0.08 | 0.63 | 0.09 | 0.56 | 0.11 | 0.49 | 0.13 | 0.34 | 0.18 |
| Delta/Theta_C4 | 0.73 | -0.06 | 0.95 | -0.01 | 0.43 | -0.15 | 0.22 | 0.22 | 0.65 | 0.08 | 0.88 | -0.03 | 0.87 | 0.03 | 0.94 | 0.01 | 0.87 | 0.03 | 0.91 | -0.02 |
| Delta/Theta_Cz | 0.55 | 0.11 | 0.55 | 0.11 | 0.30 | 0.19 | 0.09 | 0.31 | 0.27 | 0.20 | 0.57 | 0.11 | 0.43 | 0.15 | 0.37 | 0.17 | 0.13 | 0.28 | 0.10 | 0.30 |
| Delta/Theta_Pz | 0.35 | -0.18 | 0.40 | -0.16 | 0.17 | -0.25 | 0.46 | 0.14 | 0.97 | -0.01 | 0.51 | -0.12 | 0.91 | -0.02 | 0.78 | -0.05 | 0.66 | 0.08 | 0.66 | 0.08 |
| Delta/Theta_Oz | 0.23 | -0.22 | 0.20 | -0.24 | 0.11 | -0.29 | 0.55 | -0.11 | 0.97 | 0.01 | 0.20 | -0.24 | 0.65 | -0.08 | 0.56 | -0.11 | 0.96 | -0.01 | 0.82 | 0.04 |
| Theta/Beta_Fz | 0.02 | -0.40 | 0.09 | -0.31 | 0.13 | -0.28 | 0.35 | -0.17 | 0.09 | -0.31 | 0.58 | -0.10 | 0.22 | -0.23 | 0.13 | -0.28 | 0.14 | -0.27 | 0.04 | -0.36 |
| Theta/Beta_C3 | 0.15 | -0.26 | 0.33 | -0.18 | 0.51 | -0.12 | 0.23 | -0.22 | 0.14 | -0.27 | 0.93 | 0.02 | 0.39 | -0.16 | 0.29 | -0.20 | 0.24 | -0.22 | 0.28 | -0.20 |
| Theta/Beta_C4 | 0.06 | -0.34 | 0.20 | -0.24 | 0.54 | -0.12 | 0.12 | -0.28 | 0.08 | -0.32 | 0.71 | -0.07 | 0.32 | -0.19 | 0.26 | -0.21 | 0.33 | -0.18 | 0.19 | -0.24 |
| Theta/Beta_Cz | **0.01** | **-0.48** | 0.05 | -0.36 | 0.10 | -0.30 | 0.19 | -0.24 | 0.08 | -0.32 | 0.56 | -0.11 | 0.25 | -0.21 | 0.10 | -0.30 | 0.15 | -0.27 | 0.07 | -0.33 |
| Theta/Beta_Pz | 0.11 | -0.29 | 0.28 | -0.20 | 0.52 | -0.12 | 0.30 | -0.19 | 0.13 | -0.28 | 0.97 | 0.01 | 0.40 | -0.16 | 0.26 | -0.21 | 0.21 | -0.23 | 0.15 | -0.26 |
| Theta/Beta_Oz | 0.33 | -0.18 | 0.59 | -0.10 | 0.67 | -0.08 | 0.62 | -0.09 | 0.32 | -0.18 | 0.65 | 0.09 | 0.95 | 0.01 | 0.66 | -0.08 | 0.90 | -0.02 | 0.45 | -0.14 |
| Alpha/Beta_Fz | 0.46 | -0.14 | 0.65 | -0.08 | 0.50 | -0.13 | 0.32 | -0.18 | 0.99 | 0.00 | 0.92 | -0.02 | 0.53 | -0.12 | 0.53 | -0.12 | 0.32 | -0.19 | 0.09 | -0.31 |
| Alpha/Beta_C3 | 0.09 | -0.31 | 0.19 | -0.24 | 0.23 | -0.22 | 0.08 | -0.31 | 0.61 | -0.10 | 0.64 | -0.09 | 0.28 | -0.20 | 0.16 | -0.26 | 0.23 | -0.22 | 0.06 | -0.35 |
| Alpha/Beta_C4 | 0.37 | -0.17 | 0.58 | -0.10 | 0.98 | -0.01 | 0.35 | -0.17 | 1.00 | 0.00 | 0.90 | 0.02 | 0.84 | -0.04 | 0.72 | -0.07 | 0.46 | -0.14 | 0.34 | -0.18 |
| Alpha/Beta_Cz | 0.05 | -0.35 | 0.13 | -0.28 | 0.21 | -0.23 | 0.14 | -0.27 | 0.74 | -0.06 | 0.43 | -0.15 | 0.29 | -0.20 | 0.17 | -0.25 | 0.17 | -0.25 | 0.06 | -0.34 |
| Alpha/Beta_Pz | 0.46 | -0.14 | 0.77 | -0.06 | 0.76 | -0.06 | 0.20 | -0.24 | 0.52 | -0.12 | 0.92 | 0.02 | 0.40 | -0.16 | 0.33 | -0.18 | 0.29 | -0.19 | 0.06 | -0.34 |
| Alpha/Beta_Oz | 0.80 | -0.05 | 0.96 | -0.01 | 0.75 | -0.06 | 0.77 | -0.05 | 0.94 | 0.01 | 0.82 | 0.04 | 0.67 | -0.08 | 0.47 | -0.13 | 0.38 | -0.16 | 0.15 | -0.26 |

**Supplementary table S23.** **The correlation analysis between the resting-state EEG COH strengths and the mean RD values of impaired WM tracts in patients with NMOSD (P value and r value).** Pearson correlation analysis or Spearman correlation analysis were used according to the normality distributions of the data, with age as covariant. P<0.05 with false discovery rate corrected was considered significant, marked with **bold font**.

|  | ATR_L | | ATR_R | | CST_L | | CGH_R | | Fmaj | | Fmin | | IFO_L | | IFO_R | | ILF_L | | ILF_R | |
| --- | --- | --- | --- | --- | --- | --- | --- | --- | --- | --- | --- | --- | --- | --- | --- | --- | --- | --- | --- | --- |
|  | P | r | P | r | P | r | P | r | P | r | P | r | P | r | P | r | P | r | P | r |
| Delta_Fz | 0.37 | -0.17 | 0.46 | -0.14 | 0.54 | -0.12 | 0.34 | -0.18 | 0.04 | -0.36 | 0.12 | -0.29 | 0.07 | -0.32 | 0.26 | -0.21 | 0.25 | -0.21 | 0.25 | -0.21 |
| Delta_C3 | 0.13 | 0.28 | 0.17 | 0.25 | 0.10 | 0.30 | 0.74 | 0.06 | 0.19 | 0.24 | 0.11 | 0.29 | 0.19 | 0.24 | 0.08 | 0.32 | 0.26 | 0.21 | 0.39 | 0.16 |
| Delta_C4 | 0.31 | 0.19 | 0.34 | 0.18 | 0.48 | 0.13 | 0.92 | 0.02 | 0.83 | 0.04 | 0.50 | 0.13 | 0.99 | 0.00 | 0.61 | 0.09 | 0.99 | 0.00 | 0.80 | -0.05 |
| Delta_Cz | 0.17 | -0.25 | 0.14 | -0.27 | 0.23 | -0.22 | 0.85 | -0.03 | 0.35 | -0.17 | 0.11 | -0.29 | 0.13 | -0.28 | 0.36 | -0.17 | 0.26 | -0.21 | 0.29 | -0.20 |
| Delta_Pz | 0.26 | -0.21 | 0.07 | -0.33 | 0.13 | -0.28 | 0.30 | -0.19 | 0.54 | -0.11 | 0.33 | -0.18 | 0.30 | -0.19 | 0.22 | -0.23 | 0.22 | -0.23 | 0.08 | -0.32 |
| Delta_Oz | 0.21 | -0.23 | 0.13 | -0.28 | 0.34 | -0.18 | 0.05 | -0.35 | 0.11 | -0.29 | 0.07 | -0.33 | 0.03 | -0.40 | 0.12 | -0.29 | 0.01 | -0.44 | 0.05 | -0.36 |
| Theta_Fz | 0.20 | -0.24 | 0.17 | -0.25 | 0.26 | -0.21 | 0.19 | -0.24 | 0.02 | -0.42 | 0.06 | -0.35 | 0.02 | -0.42 | 0.04 | -0.36 | 0.02 | -0.41 | 0.01 | -0.45 |
| Theta_C3 | 0.82 | -0.04 | 0.81 | -0.05 | 0.94 | -0.01 | 0.60 | 0.10 | 0.76 | -0.06 | 0.75 | -0.06 | 0.42 | -0.15 | 0.56 | -0.11 | 0.26 | -0.21 | 0.04 | -0.38 |
| Theta_C4 | 0.57 | -0.11 | 0.56 | -0.11 | 0.83 | -0.04 | 0.66 | -0.08 | 0.14 | -0.27 | 0.37 | -0.17 | 0.09 | -0.31 | 0.22 | -0.23 | 0.19 | -0.24 | 0.13 | -0.28 |
| Theta_Cz | 0.03 | -0.38 | 0.04 | -0.37 | 0.05 | -0.36 | 0.37 | -0.17 | 0.23 | -0.22 | 0.02 | -0.42 | 0.02 | -0.43 | 0.04 | -0.38 | 0.02 | -0.42 | 0.01 | -0.49 |
| Theta_Pz | 0.88 | -0.03 | 0.43 | -0.15 | 0.55 | -0.11 | 0.54 | -0.11 | 0.28 | -0.20 | 0.69 | -0.07 | 0.37 | -0.17 | 0.40 | -0.16 | 0.23 | -0.22 | 0.13 | -0.28 |
| Theta_Oz | 0.25 | -0.21 | 0.24 | -0.22 | 0.58 | -0.10 | 0.11 | -0.29 | 0.04 | -0.38 | 0.10 | -0.31 | 0.01 | -0.45 | 0.05 | -0.35 | 0.02 | -0.42 | 0.03 | -0.38 |
| Alpha_Fz | 0.66 | -0.08 | 0.53 | -0.12 | 0.70 | -0.07 | 0.07 | -0.33 | 0.53 | -0.12 | 0.21 | -0.23 | 0.11 | -0.29 | 0.09 | -0.31 | 0.23 | -0.22 | 0.11 | -0.30 |
| Alpha_C3 | 0.51 | -0.12 | 0.79 | -0.05 | 0.29 | -0.20 | 0.93 | -0.02 | 0.84 | 0.04 | 0.67 | -0.08 | 0.19 | -0.24 | 0.21 | -0.23 | 0.11 | -0.29 | 0.04 | -0.37 |
| Alpha_C4 | 0.72 | -0.07 | 0.52 | -0.12 | 0.76 | -0.06 | 0.84 | -0.04 | 0.97 | 0.01 | 0.43 | -0.15 | 0.31 | -0.19 | 0.34 | -0.18 | 0.28 | -0.20 | 0.18 | -0.25 |
| Alpha_Cz | 0.38 | -0.16 | 0.43 | -0.15 | 0.46 | -0.14 | 0.79 | -0.05 | 0.78 | 0.05 | 0.36 | -0.17 | 0.33 | -0.18 | 0.36 | -0.17 | 0.17 | -0.25 | 0.15 | -0.26 |
| Alpha_Pz | 0.95 | -0.01 | 0.59 | -0.10 | 0.93 | -0.02 | 0.24 | -0.22 | 0.40 | -0.16 | 0.58 | -0.10 | 0.26 | -0.21 | 0.29 | -0.20 | 0.42 | -0.15 | 0.17 | -0.25 |
| Alpha_Oz | 0.85 | -0.04 | 0.55 | -0.11 | 0.74 | -0.06 | 0.21 | -0.23 | 0.78 | -0.05 | 0.17 | -0.25 | 0.19 | -0.24 | 0.19 | -0.24 | 0.28 | -0.20 | 0.18 | -0.25 |
| Beta_Fz | 0.49 | -0.13 | 0.38 | -0.16 | 0.68 | -0.08 | 0.33 | -0.18 | 0.33 | -0.18 | 0.06 | -0.34 | 0.07 | -0.33 | 0.14 | -0.27 | 0.23 | -0.22 | 0.09 | -0.31 |
| Beta_C3 | 0.44 | -0.14 | 0.74 | -0.06 | 0.27 | -0.21 | 0.92 | -0.02 | 0.68 | -0.08 | 0.42 | -0.15 | 0.36 | -0.17 | 0.63 | -0.09 | 0.58 | -0.10 | 0.23 | -0.22 |
| Beta_C4 | 0.93 | -0.02 | 0.76 | 0.06 | 0.75 | -0.06 | 0.52 | 0.12 | 0.29 | -0.20 | 0.59 | -0.10 | 0.18 | -0.25 | 0.57 | -0.11 | 0.17 | -0.25 | 0.23 | -0.22 |
| Beta_Cz | 0.11 | -0.30 | 0.20 | -0.24 | 0.09 | -0.31 | 0.78 | 0.05 | 0.34 | -0.18 | 0.02 | -0.41 | 0.05 | -0.35 | 0.13 | -0.28 | 0.25 | -0.21 | 0.28 | -0.20 |
| Beta_Pz | 0.33 | 0.18 | 0.73 | 0.07 | 0.44 | 0.14 | 0.98 | 0.00 | 0.63 | 0.09 | 0.66 | 0.08 | 0.90 | 0.02 | 0.72 | 0.07 | 0.94 | -0.01 | 0.95 | 0.01 |
| Beta_Oz | 0.64 | -0.09 | 0.94 | -0.01 | 0.89 | -0.02 | 0.51 | -0.12 | 0.89 | -0.03 | 0.41 | -0.15 | 0.31 | -0.19 | 0.52 | -0.12 | 0.63 | -0.09 | 0.90 | -0.02 |
| Gamma_Fz | 0.85 | -0.04 | 0.98 | 0.00 | 0.31 | 0.19 | 0.26 | 0.21 | 0.37 | -0.17 | 0.58 | -0.10 | 0.70 | -0.07 | 0.66 | -0.08 | 0.87 | -0.03 | 0.44 | -0.14 |
| Gamma_C3 | 0.83 | -0.04 | 0.81 | 0.05 | 0.63 | -0.09 | 0.78 | -0.05 | 0.84 | 0.04 | 0.98 | 0.00 | 0.86 | -0.03 | 0.95 | -0.01 | 0.67 | -0.08 | 0.32 | -0.18 |
| Gamma_C4 | 0.52 | -0.12 | 0.99 | 0.00 | 0.47 | -0.14 | 0.81 | 0.05 | 0.47 | -0.13 | 0.84 | -0.04 | 0.48 | -0.13 | 0.62 | -0.09 | 0.79 | -0.05 | 0.56 | -0.11 |
| Gamma_Cz | 0.00 | -0.52 | 0.02 | -0.42 | 0.00 | -0.51 | 0.42 | -0.15 | 0.07 | -0.33 | 0.00 | -0.51 | 0.00 | -0.55 | 0.01 | -0.49 | 0.02 | -0.42 | 0.04 | -0.37 |
| Gamma_Pz | 0.36 | 0.17 | 0.74 | 0.06 | 0.53 | 0.12 | 0.96 | -0.01 | 0.57 | 0.11 | 0.42 | 0.15 | 0.65 | 0.08 | 0.56 | 0.11 | 0.96 | 0.01 | 0.94 | -0.01 |
| Gamma_Oz | 0.29 | -0.20 | 0.99 | 0.00 | 0.73 | 0.07 | 0.42 | -0.15 | 0.38 | -0.16 | 0.93 | -0.02 | 0.40 | -0.16 | 0.35 | -0.17 | 0.38 | -0.16 | 0.34 | -0.18 |

**Supplementary table S24.** **The correlation analysis between the resting-state EEG iCOH strengths and the mean RD values of impaired WM tracts in patients with NMOSD (P value and r value).** Pearson correlation analysis or Spearman correlation analysis were used according to the normality distributions of the data, with age as covariant. P<0.05 with false discovery rate corrected was considered significant, marked with **bold font**.

|  | ATR_L | | ATR_R | | CST_L | | CGH_R | | Fmaj | | Fmin | | IFO_L | | IFO_R | | ILF_L | | ILF_R | |
| --- | --- | --- | --- | --- | --- | --- | --- | --- | --- | --- | --- | --- | --- | --- | --- | --- | --- | --- | --- | --- |
|  | P | r | P | r | P | r | P | r | P | r | P | r | P | r | P | r | P | r | P | r |
| Delta_Fz | 1.00 | 0.00 | 0.77 | -0.05 | 0.87 | 0.03 | 0.71 | 0.07 | 0.49 | 0.13 | 0.66 | -0.08 | 0.70 | 0.07 | 0.39 | 0.16 | 1.00 | 0.00 | 0.91 | 0.02 |
| Delta_C3 | 0.17 | 0.25 | 0.25 | 0.21 | 0.39 | 0.16 | 0.67 | 0.08 | 0.07 | 0.33 | 0.28 | 0.20 | 0.27 | 0.20 | 0.16 | 0.26 | 0.62 | 0.09 | 0.34 | 0.18 |
| Delta_C4 | 0.98 | 0.00 | 0.83 | -0.04 | 0.92 | 0.02 | 0.45 | -0.14 | 0.56 | 0.11 | 1.00 | 0.00 | 0.55 | 0.11 | 0.57 | 0.11 | 0.97 | 0.01 | 0.77 | 0.06 |
| Delta_Cz | 0.84 | -0.04 | 0.91 | -0.02 | 0.90 | 0.02 | 0.97 | -0.01 | 0.65 | 0.09 | 0.88 | -0.03 | 0.75 | 0.06 | 0.36 | 0.17 | 0.93 | 0.02 | 0.46 | 0.14 |
| Delta_Pz | 0.51 | -0.12 | 0.32 | -0.18 | 0.27 | -0.20 | 0.45 | -0.14 | 0.59 | -0.10 | 0.44 | -0.15 | 0.30 | -0.19 | 0.41 | -0.15 | 0.06 | -0.35 | 0.24 | -0.22 |
| Delta_Oz | 0.45 | -0.14 | 0.49 | -0.13 | 0.55 | -0.11 | 0.09 | -0.31 | 0.35 | -0.17 | 0.20 | -0.24 | 0.32 | -0.19 | 0.50 | -0.13 | 0.23 | -0.22 | 0.35 | -0.17 |
| Theta_Fz | 0.30 | 0.19 | 0.33 | 0.18 | 0.36 | 0.17 | 0.36 | 0.17 | 0.65 | 0.09 | 0.11 | 0.29 | 0.45 | 0.14 | 0.49 | 0.13 | 0.93 | -0.02 | 0.44 | -0.14 |
| Theta_C3 | 0.54 | 0.12 | 0.61 | 0.10 | 0.34 | 0.18 | 0.99 | 0.00 | 0.65 | 0.08 | 0.39 | 0.16 | 0.85 | 0.04 | 0.83 | 0.04 | 0.43 | -0.15 | 0.36 | -0.17 |
| Theta_C4 | 0.29 | 0.20 | 0.13 | 0.28 | 0.26 | 0.21 | 0.65 | -0.09 | 0.79 | 0.05 | 0.04 | 0.36 | 0.58 | 0.10 | 0.68 | 0.08 | 0.73 | -0.07 | 0.62 | -0.09 |
| Theta_Cz | 0.56 | 0.11 | 0.83 | 0.04 | 0.84 | -0.04 | 0.98 | 0.00 | 0.89 | 0.03 | 0.50 | 0.13 | 0.95 | -0.01 | 0.99 | 0.00 | 0.29 | -0.20 | 0.16 | -0.26 |
| Theta_Pz | 0.40 | 0.16 | 0.44 | 0.14 | 0.42 | 0.15 | 0.89 | 0.03 | 0.78 | -0.05 | 0.48 | 0.13 | 0.80 | -0.05 | 0.83 | -0.04 | 0.38 | -0.16 | 0.11 | -0.30 |
| Theta_Oz | 0.70 | 0.07 | 0.30 | 0.19 | 0.58 | 0.10 | 0.66 | 0.08 | 0.72 | -0.07 | 0.70 | 0.07 | 0.34 | -0.18 | 0.52 | -0.12 | 0.18 | -0.25 | 0.13 | -0.28 |
| Alpha_Fz | 0.17 | 0.25 | 0.19 | 0.24 | 0.12 | 0.29 | 0.73 | 0.07 | 0.41 | 0.15 | 0.40 | 0.16 | 0.89 | 0.03 | 0.80 | 0.05 | 0.85 | -0.04 | 0.61 | -0.10 |
| Alpha_C3 | 0.69 | 0.07 | 0.67 | 0.08 | 0.75 | -0.06 | 0.86 | 0.03 | 0.48 | 0.13 | 0.87 | -0.03 | 0.48 | -0.13 | 0.65 | -0.08 | 0.42 | -0.15 | 0.23 | -0.22 |
| Alpha_C4 | 0.37 | -0.17 | 0.11 | -0.29 | 0.43 | -0.15 | 0.08 | -0.32 | 0.88 | -0.03 | 0.11 | -0.29 | 0.19 | -0.24 | 0.15 | -0.27 | 0.17 | -0.25 | 0.07 | -0.33 |
| Alpha_Cz | 0.51 | 0.12 | 0.74 | 0.06 | 0.79 | 0.05 | 0.82 | -0.04 | 0.19 | 0.24 | 0.78 | 0.05 | 0.67 | 0.08 | 0.55 | 0.11 | 0.85 | 0.04 | 0.88 | -0.03 |
| Alpha_Pz | 0.04 | 0.37 | 0.15 | 0.27 | 0.07 | 0.33 | 0.69 | -0.07 | 0.24 | 0.22 | 0.20 | 0.24 | 0.46 | 0.14 | 0.40 | 0.16 | 0.60 | 0.10 | 0.89 | -0.03 |
| Alpha_Oz | 0.55 | 0.11 | 0.34 | 0.18 | 0.65 | 0.09 | 0.49 | 0.13 | 0.87 | 0.03 | 0.91 | 0.02 | 0.47 | -0.14 | 0.63 | -0.09 | 0.35 | -0.17 | 0.34 | -0.18 |
| Beta_Fz | 0.66 | 0.08 | 0.90 | 0.02 | 0.63 | -0.09 | 0.99 | 0.00 | 0.96 | -0.01 | 0.39 | -0.16 | 0.74 | -0.06 | 0.98 | 0.00 | 0.80 | 0.05 | 0.87 | -0.03 |
| Beta_C3 | 0.97 | 0.01 | 0.88 | -0.03 | 0.51 | -0.12 | 0.31 | -0.19 | 0.78 | 0.05 | 0.31 | -0.19 | 0.57 | -0.11 | 0.89 | -0.02 | 0.95 | -0.01 | 0.54 | 0.11 |
| Beta_C4 | 0.85 | 0.04 | 0.98 | 0.00 | 0.56 | -0.11 | 0.40 | -0.16 | 0.90 | 0.02 | 0.33 | -0.18 | 0.76 | -0.06 | 0.91 | 0.02 | 0.76 | 0.06 | 0.69 | 0.07 |
| Beta_Cz | 0.74 | 0.06 | 0.85 | 0.03 | 0.46 | -0.14 | 0.84 | -0.04 | 0.73 | 0.06 | 0.44 | -0.14 | 0.81 | -0.04 | 0.95 | 0.01 | 0.80 | 0.05 | 0.56 | 0.11 |
| Beta_Pz | 0.94 | -0.01 | 0.86 | -0.03 | 0.35 | -0.17 | 0.61 | -0.10 | 0.65 | -0.09 | 0.24 | -0.22 | 0.52 | -0.12 | 0.89 | -0.03 | 1.00 | 0.00 | 0.93 | 0.02 |
| Beta_Oz | 0.15 | -0.27 | 0.13 | -0.28 | 0.06 | -0.35 | 0.29 | -0.19 | 0.18 | -0.24 | 0.01 | -0.44 | 0.04 | -0.37 | 0.18 | -0.25 | 0.09 | -0.31 | 0.34 | -0.18 |
| Gamma_Fz | 0.57 | 0.11 | 0.75 | 0.06 | 0.22 | 0.23 | 0.99 | 0.00 | 0.98 | 0.00 | 0.85 | 0.04 | 0.38 | 0.16 | 0.52 | 0.12 | 0.29 | 0.19 | 0.42 | 0.15 |
| Gamma_C3 | 0.29 | 0.20 | 0.94 | -0.01 | 0.27 | 0.20 | 0.40 | -0.16 | 0.67 | 0.08 | 0.83 | -0.04 | 0.92 | 0.02 | 0.92 | 0.02 | 0.96 | 0.01 | 0.98 | -0.01 |
| Gamma_C4 | 0.33 | 0.18 | 0.40 | 0.16 | 0.26 | 0.21 | 0.65 | 0.08 | 0.80 | 0.05 | 0.48 | 0.13 | 0.50 | 0.13 | 0.48 | 0.13 | 0.63 | 0.09 | 0.65 | 0.09 |
| Gamma_Cz | 0.86 | 0.03 | 0.91 | -0.02 | 0.46 | 0.14 | 0.44 | -0.14 | 0.84 | 0.04 | 0.97 | 0.01 | 0.75 | 0.06 | 0.83 | 0.04 | 0.87 | 0.03 | 0.57 | 0.11 |
| Gamma_Pz | 0.22 | 0.23 | 0.29 | 0.20 | 0.10 | 0.30 | 0.67 | 0.08 | 0.49 | 0.13 | 0.49 | 0.13 | 0.45 | 0.14 | 0.36 | 0.17 | 0.35 | 0.17 | 0.16 | 0.26 |
| Gamma_Oz | 0.80 | 0.05 | 0.62 | -0.09 | 0.28 | 0.20 | 0.44 | -0.14 | 0.71 | -0.07 | 0.69 | -0.07 | 0.65 | -0.08 | 0.47 | -0.13 | 0.64 | -0.09 | 0.40 | -0.16 |

**Supplementary table S25.** **The correlation analysis between the resting-state EEG PLV strengths and the mean RD values of impaired WM tracts in patients with NMOSD (P value and r value).** Pearson correlation analysis or Spearman correlation analysis were used according to the normality distributions of the data, with age as covariant. P<0.05 with false discovery rate corrected was considered significant, marked with **bold font**.

|  | ATR_L | | ATR_R | | CST_L | | CGH_R | | Fmaj | | Fmin | | IFO_L | | IFO_R | | ILF_L | | ILF_R | |
| --- | --- | --- | --- | --- | --- | --- | --- | --- | --- | --- | --- | --- | --- | --- | --- | --- | --- | --- | --- | --- |
|  | P | r | P | r | P | r | P | r | P | r | P | r | P | r | P | r | P | r | P | r |
| Delta_Fz | 0.47 | -0.13 | 0.59 | -0.10 | 0.50 | -0.13 | 0.16 | -0.26 | 0.08 | -0.32 | 0.17 | -0.25 | 0.14 | -0.27 | 0.37 | -0.17 | 0.35 | -0.17 | 0.21 | -0.23 |
| Delta_C3 | 0.63 | 0.09 | 0.51 | 0.12 | 0.25 | 0.21 | 0.45 | 0.14 | 0.60 | 0.10 | 0.63 | 0.09 | 0.95 | 0.01 | 0.55 | 0.11 | 0.67 | 0.08 | 0.72 | 0.07 |
| Delta_C4 | 0.49 | 0.13 | 0.55 | 0.11 | 0.50 | 0.12 | 0.78 | 0.05 | 0.67 | 0.08 | 0.69 | 0.08 | 0.86 | -0.03 | 0.85 | 0.03 | 0.68 | -0.08 | 0.31 | -0.19 |
| Delta_Cz | 0.39 | -0.16 | 0.56 | -0.11 | 0.34 | -0.18 | 0.43 | 0.15 | 0.43 | -0.15 | 0.23 | -0.22 | 0.14 | -0.27 | 0.48 | -0.13 | 0.27 | -0.20 | 0.40 | -0.16 |
| Delta_Pz | 0.47 | -0.14 | 0.19 | -0.24 | 0.18 | -0.25 | 0.61 | -0.09 | 0.76 | -0.06 | 0.55 | -0.11 | 0.60 | -0.10 | 0.53 | -0.12 | 0.34 | -0.18 | 0.12 | -0.28 |
| Delta_Oz | 0.28 | -0.20 | 0.30 | -0.19 | 0.32 | -0.18 | 0.09 | -0.31 | 0.37 | -0.17 | 0.18 | -0.25 | 0.07 | -0.33 | 0.18 | -0.25 | 0.04 | -0.36 | 0.10 | -0.30 |
| Theta_Fz | 0.12 | -0.28 | 0.13 | -0.28 | 0.23 | -0.22 | 0.10 | -0.30 | 0.02 | -0.43 | 0.03 | -0.39 | 0.02 | -0.43 | 0.04 | -0.37 | 0.02 | -0.41 | 0.01 | -0.46 |
| Theta_C3 | 0.31 | -0.19 | 0.48 | -0.13 | 0.53 | -0.12 | 0.99 | 0.00 | 0.50 | -0.13 | 0.42 | -0.15 | 0.15 | -0.26 | 0.21 | -0.23 | 0.10 | -0.30 | 0.03 | -0.39 |
| Theta_C4 | 0.17 | -0.25 | 0.31 | -0.19 | 0.72 | -0.07 | 0.43 | -0.15 | 0.16 | -0.26 | 0.22 | -0.23 | 0.03 | -0.39 | 0.08 | -0.32 | 0.01 | -0.44 | 0.01 | -0.46 |
| Theta_Cz | 0.01 | -0.47 | 0.05 | -0.36 | 0.04 | -0.37 | 0.49 | -0.13 | 0.06 | -0.34 | 0.01 | -0.46 | 0.00 | -0.50 | 0.02 | -0.43 | 0.02 | -0.41 | 0.01 | -0.48 |
| Theta_Pz | 0.79 | 0.05 | 0.81 | -0.04 | 0.91 | -0.02 | 0.95 | -0.01 | 0.42 | -0.15 | 0.91 | 0.02 | 0.72 | -0.07 | 0.81 | -0.05 | 0.32 | -0.18 | 0.28 | -0.20 |
| Theta_Oz | 0.22 | -0.23 | 0.31 | -0.19 | 0.47 | -0.14 | 0.09 | -0.31 | 0.03 | -0.38 | 0.11 | -0.29 | 0.01 | -0.43 | 0.08 | -0.32 | 0.02 | -0.43 | 0.03 | -0.40 |
| Alpha_Fz | 0.66 | -0.08 | 0.69 | -0.08 | 0.62 | -0.09 | 0.20 | -0.24 | 0.84 | -0.04 | 0.39 | -0.16 | 0.20 | -0.24 | 0.23 | -0.22 | 0.20 | -0.23 | 0.23 | -0.22 |
| Alpha_C3 | 0.35 | -0.17 | 0.83 | -0.04 | 0.26 | -0.21 | 0.98 | 0.01 | 0.92 | -0.02 | 0.76 | -0.06 | 0.15 | -0.26 | 0.14 | -0.27 | 0.10 | -0.30 | 0.11 | -0.29 |
| Alpha_C4 | 0.36 | -0.17 | 0.30 | -0.19 | 0.45 | -0.14 | 0.45 | -0.14 | 0.79 | 0.05 | 0.35 | -0.17 | 0.19 | -0.24 | 0.14 | -0.27 | 0.18 | -0.25 | 0.14 | -0.27 |
| Alpha_Cz | 0.29 | -0.19 | 0.47 | -0.13 | 0.39 | -0.16 | 0.78 | -0.05 | 0.80 | 0.05 | 0.38 | -0.16 | 0.22 | -0.23 | 0.29 | -0.20 | 0.12 | -0.29 | 0.24 | -0.22 |
| Alpha_Pz | 0.50 | 0.13 | 0.69 | 0.07 | 0.76 | 0.06 | 0.49 | -0.13 | 0.98 | 0.00 | 0.85 | 0.04 | 0.62 | -0.09 | 0.75 | -0.06 | 0.70 | -0.07 | 0.35 | -0.17 |
| Alpha_Oz | 0.96 | -0.01 | 0.91 | -0.02 | 0.73 | -0.07 | 0.50 | -0.12 | 0.91 | -0.02 | 0.41 | -0.15 | 0.32 | -0.19 | 0.29 | -0.19 | 0.32 | -0.19 | 0.32 | -0.18 |
| Beta_Fz | 0.41 | -0.15 | 0.30 | -0.19 | 0.25 | -0.21 | 0.46 | -0.14 | 0.53 | -0.12 | 0.04 | -0.37 | 0.07 | -0.33 | 0.12 | -0.29 | 0.23 | -0.22 | 0.16 | -0.26 |
| Beta_C3 | 0.21 | -0.23 | 0.53 | -0.12 | 0.12 | -0.28 | 0.90 | -0.02 | 0.50 | -0.12 | 0.21 | -0.23 | 0.14 | -0.27 | 0.29 | -0.20 | 0.45 | -0.14 | 0.21 | -0.23 |
| Beta_C4 | 0.96 | -0.01 | 0.59 | 0.10 | 0.79 | -0.05 | 0.16 | 0.26 | 0.40 | -0.16 | 0.80 | -0.05 | 0.40 | -0.16 | 0.97 | -0.01 | 0.40 | -0.16 | 0.60 | -0.10 |
| Beta_Cz | 0.14 | -0.27 | 0.28 | -0.20 | 0.09 | -0.31 | 0.70 | 0.07 | 0.57 | -0.10 | 0.04 | -0.37 | 0.10 | -0.30 | 0.22 | -0.23 | 0.40 | -0.16 | 0.44 | -0.14 |
| Beta_Pz | 0.37 | 0.17 | 0.67 | 0.08 | 0.50 | 0.13 | 0.74 | -0.06 | 0.90 | 0.02 | 0.86 | 0.03 | 0.96 | -0.01 | 0.84 | 0.04 | 1.00 | 0.00 | 0.78 | 0.05 |
| Beta_Oz | 0.64 | -0.09 | 0.76 | -0.06 | 0.50 | -0.13 | 0.65 | -0.08 | 0.98 | 0.00 | 0.28 | -0.20 | 0.42 | -0.15 | 0.57 | -0.11 | 0.92 | -0.02 | 0.86 | 0.03 |
| Gamma_Fz | 0.58 | -0.10 | 0.89 | -0.03 | 0.46 | 0.14 | 0.22 | 0.23 | 0.24 | -0.22 | 0.55 | -0.11 | 0.63 | -0.09 | 0.61 | -0.10 | 0.76 | -0.06 | 0.43 | -0.15 |
| Gamma_C3 | 0.51 | -0.12 | 0.99 | 0.00 | 0.34 | -0.18 | 0.65 | -0.08 | 0.82 | -0.04 | 0.75 | -0.06 | 0.68 | -0.08 | 0.64 | -0.09 | 0.54 | -0.12 | 0.21 | -0.23 |
| Gamma_C4 | 0.35 | -0.17 | 0.68 | -0.08 | 0.45 | -0.14 | 0.76 | 0.06 | 0.33 | -0.18 | 0.84 | -0.04 | 0.75 | -0.06 | 0.69 | -0.07 | 0.75 | 0.06 | 0.73 | -0.06 |
| Gamma_Cz | **0.00** | **-0.54** | 0.01 | -0.45 | 0.01 | -0.49 | 0.29 | -0.20 | 0.01 | -0.44 | **0.00** | **-0.55** | **0.00** | **-0.57** | **0.00** | **-0.53** | 0.03 | -0.39 | 0.08 | -0.32 |
| Gamma_Pz | 0.31 | 0.19 | 0.68 | 0.08 | 0.38 | 0.16 | 0.88 | -0.03 | 0.48 | 0.13 | 0.42 | 0.15 | 0.57 | 0.11 | 0.52 | 0.12 | 0.94 | 0.01 | 0.92 | -0.02 |
| Gamma_Oz | 0.13 | -0.28 | 0.58 | -0.10 | 0.82 | -0.04 | 0.43 | -0.15 | 0.30 | -0.19 | 0.56 | -0.11 | 0.25 | -0.21 | 0.17 | -0.25 | 0.22 | -0.23 | 0.22 | -0.23 |

**Supplementary table S26.** **The correlation analysis between the resting-state EEG PLI strengths and the mean RD values of impaired WM tracts in patients with NMOSD (P value and r value).** Pearson correlation analysis or Spearman correlation analysis were used according to the normality distributions of the data, with age as covariant. P<0.05 with false discovery rate corrected was considered significant, marked with **bold font**.

|  | ATR_L | | ATR_R | | CST_L | | CGH_R | | Fmaj | | Fmin | | IFO_L | | IFO_R | | ILF_L | | ILF_R | |
| --- | --- | --- | --- | --- | --- | --- | --- | --- | --- | --- | --- | --- | --- | --- | --- | --- | --- | --- | --- | --- |
|  | P | r | P | r | P | r | P | r | P | r | P | r | P | r | P | r | P | r | P | r |
| Delta_Fz | 0.90 | -0.02 | 0.87 | -0.03 | 0.78 | -0.05 | 0.83 | 0.04 | 0.77 | 0.06 | 0.97 | 0.01 | 0.93 | 0.02 | 0.80 | 0.05 | 0.64 | -0.09 | 0.75 | -0.06 |
| Delta_C3 | 0.70 | 0.07 | 0.29 | 0.20 | 0.84 | -0.04 | 0.97 | 0.01 | 0.40 | 0.16 | 0.33 | 0.18 | 0.64 | 0.09 | 0.55 | 0.11 | 0.96 | -0.01 | 0.88 | 0.03 |
| Delta_C4 | 0.34 | -0.18 | 0.25 | -0.21 | 0.25 | -0.21 | 0.07 | -0.33 | 0.79 | -0.05 | 0.26 | -0.21 | 0.60 | -0.10 | 0.66 | -0.08 | 0.39 | -0.16 | 0.62 | -0.09 |
| Delta_Cz | 0.14 | -0.27 | 0.49 | -0.13 | 0.24 | -0.22 | 0.37 | -0.17 | 0.57 | -0.11 | 0.67 | -0.08 | 0.64 | -0.09 | 0.64 | -0.09 | 0.46 | -0.14 | 0.85 | -0.03 |
| Delta_Pz | 0.29 | -0.20 | 0.41 | -0.15 | 0.15 | -0.26 | 0.56 | -0.11 | 0.58 | -0.10 | 0.70 | -0.07 | 0.41 | -0.15 | 0.48 | -0.13 | 0.10 | -0.30 | 0.29 | -0.19 |
| Delta_Oz | 0.52 | -0.12 | 0.31 | -0.19 | 0.16 | -0.26 | 0.19 | -0.24 | 0.42 | -0.15 | 0.31 | -0.19 | 0.27 | -0.20 | 0.32 | -0.18 | 0.09 | -0.31 | 0.19 | -0.24 |
| Theta_Fz | 0.30 | 0.28 | 0.33 | 0.27 | 0.36 | 0.31 | 0.36 | 0.06 | 0.65 | -0.04 | 0.11 | 0.24 | 0.45 | 0.11 | 0.49 | 0.11 | 0.93 | 0.05 | 0.44 | -0.01 |
| Theta_C3 | 0.54 | 0.22 | 0.61 | 0.22 | 0.34 | 0.25 | 0.99 | -0.12 | 0.65 | 0.06 | 0.39 | 0.21 | 0.85 | 0.03 | 0.83 | 0.08 | 0.43 | -0.06 | 0.36 | -0.01 |
| Theta_C4 | 0.29 | 0.05 | 0.13 | 0.17 | 0.26 | 0.13 | 0.65 | -0.25 | 0.79 | -0.06 | 0.04 | 0.16 | 0.58 | -0.13 | 0.68 | -0.12 | 0.73 | -0.24 | 0.62 | -0.20 |
| Theta_Cz | 0.56 | -0.03 | 0.83 | 0.00 | 0.84 | -0.02 | 0.98 | -0.20 | 0.89 | -0.23 | 0.50 | -0.04 | 0.95 | -0.20 | 0.99 | -0.21 | 0.29 | -0.25 | 0.16 | -0.27 |
| Theta_Pz | 0.40 | 0.21 | 0.44 | 0.21 | 0.42 | 0.24 | 0.89 | -0.04 | 0.78 | -0.12 | 0.48 | 0.13 | 0.80 | -0.08 | 0.83 | -0.02 | 0.38 | -0.18 | 0.11 | -0.21 |
| Theta_Oz | 0.70 | 0.01 | 0.30 | 0.17 | 0.58 | 0.07 | 0.66 | 0.11 | 0.72 | -0.13 | 0.70 | 0.07 | 0.34 | -0.13 | 0.52 | -0.13 | 0.18 | -0.14 | 0.13 | -0.18 |
| Alpha_Fz | 0.51 | 0.12 | 0.35 | 0.17 | 0.37 | 0.17 | 0.85 | 0.03 | 0.66 | 0.08 | 0.57 | 0.11 | 0.69 | -0.07 | 0.83 | -0.04 | 0.82 | -0.04 | 0.96 | -0.01 |
| Alpha_C3 | 0.83 | 0.04 | 0.45 | 0.14 | 0.64 | -0.09 | 0.61 | -0.09 | 0.68 | 0.08 | 0.88 | 0.03 | 0.40 | -0.16 | 0.54 | -0.12 | 0.37 | -0.17 | 0.31 | -0.19 |
| Alpha_C4 | 0.23 | -0.22 | 0.13 | -0.28 | 0.28 | -0.20 | 0.09 | -0.31 | 0.46 | -0.14 | 0.04 | -0.37 | 0.08 | -0.31 | 0.09 | -0.31 | 0.11 | -0.29 | 0.09 | -0.31 |
| Alpha_Cz | 0.93 | 0.02 | 0.73 | 0.06 | 0.92 | -0.02 | 0.92 | -0.02 | 0.59 | 0.10 | 0.83 | -0.04 | 0.77 | -0.05 | 0.91 | 0.02 | 0.68 | -0.08 | 0.62 | -0.09 |
| Alpha_Pz | 0.16 | 0.26 | 0.25 | 0.21 | 0.17 | 0.26 | 0.49 | -0.13 | 0.54 | 0.12 | 0.33 | 0.18 | 0.58 | 0.10 | 0.49 | 0.13 | 0.88 | 0.03 | 0.81 | 0.04 |
| Alpha_Oz | 0.77 | 0.06 | 0.41 | 0.15 | 0.86 | 0.03 | 0.49 | 0.13 | 0.83 | -0.04 | 0.99 | 0.00 | 0.47 | -0.14 | 0.56 | -0.11 | 0.44 | -0.14 | 0.80 | -0.05 |
| Beta_Fz | 0.55 | 0.11 | 0.89 | 0.03 | 0.93 | -0.02 | 0.62 | 0.09 | 0.86 | 0.03 | 0.47 | -0.14 | 0.57 | -0.11 | 0.97 | -0.01 | 0.66 | -0.08 | 0.88 | -0.03 |
| Beta_C3 | 0.79 | 0.05 | 0.89 | 0.03 | 0.92 | -0.02 | 0.35 | -0.17 | 0.39 | 0.16 | 0.61 | -0.10 | 0.72 | -0.07 | 0.96 | 0.01 | 0.97 | -0.01 | 0.57 | 0.11 |
| Beta_C4 | 0.75 | 0.06 | 1.00 | 0.00 | 0.86 | -0.03 | 0.52 | -0.12 | 0.92 | 0.02 | 0.36 | -0.17 | 0.91 | -0.02 | 0.53 | 0.12 | 0.74 | 0.06 | 0.55 | 0.11 |
| Beta_Cz | 0.46 | 0.14 | 0.55 | 0.11 | 0.99 | 0.00 | 0.94 | 0.01 | 0.54 | 0.11 | 0.57 | -0.11 | 0.59 | -0.10 | 0.82 | 0.04 | 0.71 | -0.07 | 0.78 | 0.05 |
| Beta_Pz | 0.25 | 0.21 | 0.29 | 0.20 | 0.74 | 0.06 | 0.46 | 0.14 | 0.54 | 0.12 | 0.87 | 0.03 | 0.93 | -0.02 | 0.58 | 0.10 | 0.87 | -0.03 | 0.60 | 0.10 |
| Beta_Oz | 0.40 | -0.16 | 0.79 | -0.05 | 0.30 | -0.19 | 0.45 | -0.14 | 0.83 | -0.04 | 0.33 | -0.18 | 0.48 | -0.13 | 0.89 | -0.03 | 0.57 | -0.11 | 0.58 | 0.10 |
| Gamma_Fz | 0.69 | -0.08 | 0.91 | -0.02 | 0.92 | 0.02 | 0.85 | 0.04 | 0.36 | -0.17 | 0.81 | -0.04 | 0.85 | 0.04 | 0.94 | 0.01 | 0.41 | 0.15 | 0.36 | 0.17 |
| Gamma_C3 | 0.65 | 0.09 | 0.52 | -0.12 | 0.43 | 0.15 | 0.24 | -0.22 | 0.92 | -0.02 | 0.51 | -0.12 | 0.94 | -0.01 | 0.86 | -0.03 | 0.96 | 0.01 | 0.54 | -0.11 |
| Gamma_C4 | 0.57 | 0.11 | 0.22 | 0.23 | 0.40 | 0.16 | 0.19 | 0.24 | 1.00 | 0.00 | 0.20 | 0.24 | 0.41 | 0.15 | 0.47 | 0.13 | 0.44 | 0.14 | 0.42 | 0.15 |
| Gamma_Cz | 0.66 | 0.08 | 0.41 | 0.15 | 0.29 | 0.20 | 0.63 | -0.09 | 0.79 | -0.05 | 0.46 | 0.14 | 0.35 | 0.17 | 0.55 | 0.11 | 0.15 | 0.26 | 0.17 | 0.25 |
| Gamma_Pz | 0.10 | 0.30 | 0.11 | 0.29 | 0.07 | 0.33 | 0.73 | 0.06 | 0.49 | 0.13 | 0.33 | 0.18 | 0.26 | 0.21 | 0.17 | 0.25 | 0.13 | 0.28 | 0.08 | 0.32 |
| Gamma_Oz | 0.83 | 0.04 | 0.80 | -0.05 | 0.52 | 0.12 | 0.42 | -0.15 | 0.58 | -0.10 | 0.71 | -0.07 | 0.55 | -0.11 | 0.58 | -0.10 | 0.82 | -0.04 | 0.90 | -0.02 |

**Supplementary table S27.** **The correlation analysis between the resting-state EEG wPLI strengths and the mean RD values of impaired WM tracts in patients with NMOSD (P value and r value).** Pearson correlation analysis or Spearman correlation analysis were used according to the normality distributions of the data, with age as covariant. P<0.05 with false discovery rate corrected was considered significant, marked with **bold font**.

|  | ATR_L | | ATR_R | | CST_L | | CGH_R | | Fmaj | | Fmin | | IFO_L | | IFO_R | | ILF_L | | ILF_R | |
| --- | --- | --- | --- | --- | --- | --- | --- | --- | --- | --- | --- | --- | --- | --- | --- | --- | --- | --- | --- | --- |
|  | P | r | P | r | P | r | P | r | P | r | P | r | P | r | P | r | P | r | P | r |
| Delta_Fz | 0.47 | 0.13 | 0.21 | 0.23 | 0.12 | 0.29 | 0.40 | 0.16 | 0.78 | 0.05 | 0.55 | 0.11 | 0.32 | 0.18 | 0.28 | 0.20 | 0.17 | 0.25 | 0.05 | 0.35 |
| Delta_C3 | 0.66 | 0.08 | 0.25 | 0.21 | 0.32 | 0.18 | 0.00 | 0.52 | 0.16 | 0.26 | 0.14 | 0.27 | 0.08 | 0.32 | 0.10 | 0.30 | 0.24 | 0.22 | 0.32 | 0.19 |
| Delta_C4 | 0.80 | 0.05 | 0.37 | 0.17 | 0.30 | 0.19 | 0.32 | 0.18 | 0.43 | 0.15 | 0.35 | 0.18 | 0.25 | 0.21 | 0.31 | 0.19 | 0.20 | 0.24 | 0.35 | 0.17 |
| Delta_Cz | 0.67 | -0.08 | 0.94 | 0.01 | 0.47 | 0.14 | 0.02 | 0.42 | 0.29 | 0.20 | 0.65 | 0.09 | 0.31 | 0.19 | 0.48 | 0.13 | 0.29 | 0.20 | 0.57 | 0.11 |
| Delta_Pz | 0.47 | 0.14 | 0.30 | 0.19 | 0.25 | 0.21 | 0.04 | 0.38 | 0.18 | 0.25 | 0.18 | 0.25 | 0.05 | 0.36 | 0.05 | 0.35 | 0.03 | 0.38 | 0.06 | 0.34 |
| Delta_Oz | 0.41 | 0.15 | 0.21 | 0.23 | 0.25 | 0.21 | 0.29 | 0.20 | 0.40 | 0.16 | 0.37 | 0.17 | 0.37 | 0.17 | 0.43 | 0.15 | 0.31 | 0.19 | 0.51 | 0.12 |
| Theta_Fz | 0.64 | 0.09 | 0.79 | 0.05 | 0.31 | 0.19 | 0.13 | 0.28 | 0.78 | 0.05 | 0.33 | 0.18 | 0.37 | 0.17 | 0.40 | 0.16 | 0.52 | 0.12 | 0.41 | 0.15 |
| Theta_C3 | 0.53 | 0.12 | 0.70 | 0.07 | 0.44 | 0.14 | 0.25 | 0.21 | 0.95 | -0.01 | 0.40 | 0.16 | 0.44 | 0.14 | 0.32 | 0.18 | 0.76 | 0.06 | 0.39 | 0.16 |
| Theta_C4 | 0.59 | 0.10 | 0.74 | 0.06 | 0.53 | 0.12 | 0.54 | 0.11 | 0.86 | 0.03 | 0.37 | 0.17 | 0.46 | 0.14 | 0.33 | 0.18 | 0.90 | 0.02 | 0.53 | 0.12 |
| Theta_Cz | 0.49 | 0.13 | 0.54 | 0.11 | 0.47 | 0.13 | 0.12 | 0.28 | 0.60 | 0.10 | 0.17 | 0.25 | 0.23 | 0.22 | 0.18 | 0.25 | 0.55 | 0.11 | 0.30 | 0.19 |
| Theta_Pz | 0.15 | 0.26 | 0.18 | 0.25 | 0.20 | 0.24 | 0.19 | 0.24 | 0.50 | 0.13 | 0.05 | 0.36 | 0.05 | 0.35 | 0.03 | 0.40 | 0.38 | 0.16 | 0.24 | 0.22 |
| Theta_Oz | 0.67 | 0.08 | 0.65 | 0.09 | 0.46 | 0.14 | 0.65 | 0.09 | 0.78 | -0.05 | 0.43 | 0.15 | 0.30 | 0.19 | 0.25 | 0.21 | 0.45 | 0.14 | 0.56 | 0.11 |
| Alpha_Fz | 0.68 | 0.08 | 0.46 | 0.14 | 0.98 | 0.00 | 0.85 | -0.04 | 0.74 | 0.06 | 0.71 | 0.07 | 0.58 | -0.10 | 0.74 | -0.06 | 0.46 | -0.14 | 0.54 | -0.11 |
| Alpha_C3 | 0.48 | -0.13 | 0.86 | -0.03 | 0.36 | -0.17 | 0.56 | -0.11 | 0.85 | 0.04 | 0.94 | -0.01 | 0.43 | -0.15 | 0.32 | -0.18 | 0.43 | -0.15 | 0.34 | -0.18 |
| Alpha_C4 | 0.18 | -0.25 | 0.36 | -0.17 | 0.22 | -0.23 | 0.23 | -0.22 | 0.59 | -0.10 | 0.28 | -0.20 | 0.12 | -0.28 | 0.10 | -0.30 | 0.10 | -0.30 | 0.15 | -0.27 |
| Alpha_Cz | 0.52 | -0.12 | 0.96 | -0.01 | 0.45 | -0.14 | 0.19 | -0.24 | 0.69 | 0.07 | 0.85 | -0.03 | 0.45 | -0.14 | 0.45 | -0.14 | 0.36 | -0.17 | 0.44 | -0.14 |
| Alpha_Pz | 0.89 | 0.03 | 0.67 | 0.08 | 0.96 | -0.01 | 0.43 | -0.15 | 0.89 | 0.02 | 0.86 | 0.03 | 0.49 | -0.13 | 0.62 | -0.09 | 0.41 | -0.15 | 0.41 | -0.15 |
| Alpha_Oz | 0.54 | -0.11 | 0.96 | 0.01 | 0.37 | -0.17 | 0.80 | -0.05 | 0.98 | -0.01 | 0.84 | -0.04 | 0.24 | -0.22 | 0.24 | -0.22 | 0.21 | -0.23 | 0.24 | -0.22 |
| Beta_Fz | 0.24 | 0.22 | 0.10 | 0.30 | 0.81 | 0.04 | 0.59 | 0.10 | 0.51 | 0.12 | 0.41 | 0.15 | 0.58 | 0.10 | 0.55 | 0.11 | 0.52 | 0.12 | 0.38 | 0.16 |
| Beta_C3 | 0.09 | 0.31 | 0.05 | 0.35 | 0.39 | 0.16 | 0.99 | 0.00 | 0.36 | 0.17 | 0.31 | 0.19 | 0.36 | 0.17 | 0.36 | 0.17 | 0.33 | 0.18 | 0.17 | 0.25 |
| Beta_C4 | 0.42 | 0.15 | 0.12 | 0.29 | 0.94 | 0.01 | 0.88 | 0.03 | 0.57 | 0.11 | 0.56 | 0.11 | 0.64 | 0.09 | 0.57 | 0.11 | 0.52 | 0.12 | 0.35 | 0.17 |
| Beta_Cz | 0.40 | 0.16 | 0.26 | 0.21 | 0.88 | -0.03 | 0.42 | 0.15 | 0.47 | 0.14 | 0.85 | 0.04 | 0.87 | 0.03 | 0.86 | 0.03 | 0.94 | 0.01 | 0.66 | 0.08 |
| Beta_Pz | 0.79 | 0.05 | 0.48 | 0.13 | 0.61 | -0.10 | 0.37 | 0.17 | 0.88 | -0.03 | 0.81 | -0.05 | 0.60 | -0.10 | 0.68 | -0.08 | 0.50 | -0.13 | 0.91 | -0.02 |
| Beta_Oz | 0.50 | -0.12 | 0.80 | -0.05 | 0.13 | -0.28 | 0.80 | -0.05 | 0.62 | -0.09 | 0.27 | -0.20 | 0.31 | -0.19 | 0.39 | -0.16 | 0.47 | -0.14 | 0.66 | -0.08 |
| Gamma_Fz | 0.97 | -0.01 | 0.84 | 0.04 | 0.90 | -0.02 | 0.57 | 0.11 | 0.75 | -0.06 | 0.96 | -0.01 | 0.58 | 0.10 | 0.60 | 0.10 | 0.19 | 0.24 | 0.20 | 0.24 |
| Gamma_C3 | 0.59 | -0.10 | 0.18 | -0.25 | 0.83 | -0.04 | 0.14 | -0.27 | 0.51 | -0.12 | 0.46 | -0.14 | 0.77 | 0.06 | 1.00 | 0.00 | 0.54 | 0.11 | 0.76 | 0.06 |
| Gamma_C4 | 0.24 | -0.22 | 0.26 | -0.21 | 0.51 | -0.12 | 0.88 | 0.03 | 0.09 | -0.31 | 0.27 | -0.20 | 0.50 | -0.12 | 0.26 | -0.21 | 0.58 | -0.10 | 0.69 | 0.07 |
| Gamma_Cz | 0.66 | -0.08 | 0.67 | -0.08 | 0.61 | -0.10 | 0.61 | 0.10 | 0.60 | -0.10 | 0.90 | 0.02 | 0.74 | 0.06 | 0.88 | 0.03 | 0.65 | 0.09 | 0.40 | 0.16 |
| Gamma_Pz | 0.37 | 0.17 | 0.20 | 0.24 | 0.37 | 0.17 | 0.27 | 0.21 | 0.20 | 0.24 | 0.28 | 0.20 | 0.18 | 0.25 | 0.11 | 0.29 | 0.06 | 0.34 | 0.01 | 0.45 |
| Gamma_Oz | 0.61 | 0.10 | 0.54 | 0.11 | 0.59 | 0.10 | 0.59 | 0.10 | 0.78 | -0.05 | 0.50 | 0.12 | 0.42 | 0.15 | 0.31 | 0.19 | 0.79 | 0.05 | 0.14 | 0.27 |
